# New and poorly known Palaearctic fungus gnats (Diptera, Sciaroidea)

**DOI:** 10.3897/BDJ.5.e11760

**Published:** 2017-03-06

**Authors:** Jukka Salmela, Levente-Péter Kolcsár

**Affiliations:** 1 Parks & Wildlife Finland (Metsähallitus), Rovaniemi, Finland; 2 Biodiversity Unit, University of Turku, Turku, Finland; 3 Babes-Bolyai University, Cluj-Napoca, Romania

**Keywords:** boreal zone, boreo-alpine species, DNA barcoding, biodiversity, taxonomy

## Abstract

**Background:**

Fungus gnats (Sciaroidea) are a globally species rich group of lower Diptera. In Europe, Fennoscandian peninsula in particular holds a notable diversity, ca. 1000 species, of which 10 % are still unnamed. Fungus gnats are predominantly terrestrial insects, but some species dwell in wetland habitats.

**New information:**

Eight new fungus gnat species, belonging to the families Keroplatidae (*Orfelia boreoalpina* Salmela sp.n.) and Mycetophilidae (*Sciophila holopaineni* Salmela sp.n., *S. curvata* Salmela sp.n., *Boletina sasakawai* Salmela & Kolcsár sp.n., *B. norokorpii* Salmela & Kolcsár sp.n., *Phronia sompio* Salmela sp.n., *P. reducta* Salmela sp.n., *P. prolongata* Salmela sp.n.), are described. Four of the species are known from Fennoscandia only whilst two are supposed to have boreo-alpine disjunct ranges, i.e. having populations in Fennoscandia and the Central European Alps. One of the species probably has a boreal range (Finnish Lapland and Central Siberia). Type material of *Boletina curta* Sasakawa & Kimura from Japan was found to consist of two species, and a further species close to these taxa is described from Finland. *Phronia elegantula* Hackman is redescribed and reported for the first time from Norway. DNA barcodes are provided for the first time for five species.

## Introduction

Sciaroidea are lower Diptera traditionally classified to the infraorder Nematocera, thread-horned flies (Amorim and Yeates 2006). Nematocera, however, is a paraphyletic group, and Sciaroidea are currently treated within Neodiptera, a monophyletic clade including Bibionomorpha and all brachyceran fly families (Wiegmann et al. 2011). Sciaroidea includes fungus gnats in the broadest sense (Bolitophilidae, Diadocidiidae, Ditomyiidae, Keroplatidae, Lygistorrhinidae, Mycetophilidae), black-winged fungus gnats (Sciaridae) and gall midges (Cecidomyiidae) (Ševčík et al. 2016b). In this paper only fungus gnats are included. Fungus gnats are mostly associated with forests, and their larvae dwell in fungal fruiting bodies, dead wood and soil; some species are associated with wetlands such as fens (Økland 1999, Søli et al. 2000, Jakovlev 2011, Salmela and Suuronen 2014).

Fungus gnats are a highly diverse group of flies, having over 5000 known species globally (Pape et al. 2009), and but this number is expected to rise (see e.g. Borkent and Wheeler 2012, Kurina and Hippa 2015). In Europe fungus gnats probably display an anomalous gradient of species richness, that is, the number of species correlates positively with latitude (Salmela et al. 2016). The northern boreal zone is probably the hot-spot of fungus gnat species richness (Kjærandsen et al. 2007), and the Fennoscandian peninsula (Norway, Sweden, Finland, Kola peninsula and Russian Karelia) in total harbours about 1000 species, of which ca. 100 still await formal description (Kjærandsen 2016).

DNA barcoding has become a standard procedure in biodiversity surveys and taxonomic studies (e.g. Stur and Borkent 2014, Nielsen et al. 2015, Hebert et al. 2016). The method is based on the observation that a fragment of the mtDNA gene COI in animals possess variation that is suitable for separating species (Hebert et al. 2003). One of the advantages of DNA barcoding has been the detection of cryptic species, that mostly consists of taxa that were previously overlooked (Nielsen et al. 2015). DNA barcoding has been used successfully in fungus gnat taxonomy (Kurina et al. 2011, Jürgenstein et al. 2015, Kurina et al. 2015, Ševčík et al. 2016a), and in the vast majority of cases the sequence variation in the COI is in accordance with morphological variation. In order to advance fungus gnat taxonomy, researchers in Norway, Finland and Russian Karelia have recently assembled a reference library including almost 1600 sequences belonging to 540 species or operational taxonomic units (J. Kjærandsen, et al. in prep.). In the present paper however, DNA barcodes are not analysed in detail and are instead used for comparative purposes. Nevertheless, the barcodes provided here should be helpful in observing these rare taxa in the future.

The new species described here belong to the genera *Orfelia* Costa, *Sciophila* Meigen, *Boletina* Staeger and *Phronia* Winnertz. *Orfelia* is a keroplatid genus with 36 Holarctic species, of which 25 are known from the Palaearctic region (Evenhuis 2006, Kurina and Jürgenstein 2013). The genus has not been revised and keys have been produced only for species present in Great Britain (Hutson et al. 1980) and Russia (Zaitzev 1994). Larvae of the genus are saproxylic (Zaitzev 1994). *Sciophila* is a mycetophilid genus with 98 Holarctic species, of which 70 are Palaearctic (Zaitzev 1982, Pape and Thompson 2013). The genus was revised by Zaitzev 1982, and 18 new species descriptions have since followed (e.g. Zaitzev 1994, Polevoi 2001). *Sciophila* larvae are mostly associated with fungal fruiting bodies, especially polypores, and most likely are spore-feeding (Jakovlev 2011) or may develop internally in fungus tissue (Zaitzev 1982). Three *Boletina* species are figured here and discussed; a review on the taxonomic status and ecology of the Holarctic species of the genus was recently provided (Salmela et al. 2016). The genus *Phronia* is rich in species, having 112 and 97 named taxa in the Holarctic and Palaearctic regions, respectively (Gagné 1975, Jakovlev and Polevoi 2009, Ševčík 2009, Pape and Thompson 2013). The first comprehensive treatment of the European *Phronia* was published by Dziedzicki 1889, a rare example of a 19th century lower Diptera publication that is still useful today. Hackman 1970 published an important paper on the eastern Fennoscandian species and North American species were revised by Gagné 1975. Plassmann 1977 provided a key to the Palaearctic fauna, but the key relies heavily on body coloration characters that are known to vary and are hard to interpret. In addition, figures in Plassmann’s publication were mostly copied from older sources and are of rather poor quality. Later Zaitzev 2003 compiled a key for Russian species and provided illustrations of male genitalia. These illustrations, despite their good quality, depict mostly only one view per species (most often the hypopygium from the ventral view). In the identification of *Phronia*, however, several characters need to be seen, such as the aedeagus and gonostylus from a variety of angles (see e.g. Chandler 1992, Kurina 2008, Jakovlev and Polevoi 2009). Although the genus *Phronia* is in urgent need of revision we describe three new species here. This is because the new taxa can be easily separated from the closely related species based ondifferences in the male hypopygium and DNA barcodes. *Phronia* larvae build cases and live upon various saproxylic substrates, but some species are observed from soil (Jakovlev 2011).

## Materials and methods

Most of the specimens studied were collected from Finland, mainly by using Malaise traps. Ethylene glycol was first used in the traps as a preservative and later the material was stored in 70 % ethanol. The morphological terminology used here follows (Søli 1997) and wing venation (Amorim and Rindal 2007). Terminology of *Sciophila* male hypopygium was adopted and slightly modified from (Kurina 2004). The following acronyms for museums and collections are used: BIOUG – Biodiversity Institute of Ontario, University of Guelph, Guelph, Canada; OSAKA – Osaka Museum of Natural History, Osaka, Japan; ZMUT – Zoological Museum, University of Turku, Turku, Finland; ZSM – Zoologische Staatsammlung München, München, Germany, TSU – Tomsk State University, Department of Invertebrate Zoology, Tomsk, Russia; FRIP – Forest Research Institute, Petrozavodsk, Russia; ZIN – Zoological Institute, Academy of Sciences, St. Petersburg, Russia; NHMO – University of Oslo, Zoological Museum, Oslo, Norway; MZHF – Finnish Museum of Natural History (Zoological Museum), University of Helsinki, Helsinki, Finland JES – Private collection of Jukka Salmela, Rovaniemi, Finland. Descriptions of the species are mostly based on specimens preserved in ethanol; male hypopygia were macerated in KOH and are preserved in separate microvials in glycerol; all specimens bear a unique catalogue number. Measurements and ratios are based on single specimens.

Images of male hypopygia were taken using an Olympus SZ61 stereomicroscope equipped with a Canon 650D camera and a LM Digital SLR Adapter. Habitus photos of the new *Orfelia* species were taken by using Olympus E520 digital camera, attached to an Olympus SZX16 stereomicroscope. Digital photos were captured using the programmes Deep Focus 3.1 and Quick PHOTO CAMERA 2.3. Extended depth of field photos were reconstructed using the software Combine ZP and were finalized with the use of Adobe Photoshop CS4. The maps were drawn by using SimppleMappr program (http://www.simplemappr.net/).

A 658 bp fragment of mitochondrial protein-encoding cytochrome c oxidase subunit I (COI) was sequenced from a total of 10 Sciaroidea specimens. Legs or 2–3 abdominal segments of the specimens were placed in 96% ethanol in a 96-well lysis microplate and dispatched to the Canadian Centre for DNA Barcoding, Biodiversity Institute of Ontario where DNA was extracted and sequenced using standard protocols and primers (deWaard et al. 2008). The fragment was successfully amplified for five taxa. The new sequences are deposited in GenBank under accession numbers KY062990-KY062993, KY200862-KY200865 and are also available below in the systematic part.

Barcodes of the Finnish specimens (all 658 bp in length, with no unambiguous bases) were submitted to the BOLD (Ratnasingham and Hebert 2007) identification engine (http://v4.boldsystems.org/index.php/IDS_OpenIdEngine) in order to search for conspecific taxa and to assess the COI divergence between the new species and the taxa available in the BOLD database. We used “current database” and “All Barcode Records on BOLD” as options in the queries. The queries were made during October 2016 and at that time the BOLD database held 4,708,558 sequences (with a minimum sequence length of 500 bp), of which 46206 belonged to the family Mycetophilidae and 3665 to the Keroplatidae. Genetic similarities presented here are based on K2P distances and were calculated by the BOLD identification engine.

## Taxon treatments

### Orfelia boreoalpina

Salmela sp. n.

urn:lsid:zoobank.org:act:B5A81F63-3F71-4A8F-B23B-ADF5CA4211D3

#### Materials

- **Type status:** Holotype. **Occurrence:** catalogNumber: DIPT-JS-2014-0233; recordedBy: M. Mäkilä; individualCount: 1; sex: M; lifeStage: adult; **Taxon:** phylum: Arthropoda; class: Insecta; order: Diptera; **Location:** country: Finland; stateProvince: Lapponia kemensis pars orientalis; municipality: Savukoski; locality: Törmäoja Conservation Area; decimalLatitude: 67.823; decimalLongitude: 29.439; **Identification:** identifiedBy: Jukka E. Salmela; **Event:** eventDate: 2014-08-07; **Record Level:** institutionCode: ZMUT
- **Type status:** Paratype. **Occurrence:** catalogNumber: BIOUG08366-D12; recordNumber: bayw.17; recordedBy: G. Sellmayer; individualCount: 1; sex: female; **Location:** country: Germany; stateProvince: Bavaria; locality: Nationalpark Bayerischer Wald, 11.3 km N of Grafenau; decimalLatitude: 48.9509; decimalLongitude: 13.422; **Event:** eventDate: 2012-09-13/22; **Record Level:** institutionCode: ZSM

#### Description

Male. Head bicolored, vertex with a triangular dark area, laterally yellowish brown (Fig. 1a, b, c). Three ocelli in shallow triangular arrangement, median ocellus smaller than laterals. Vertex covered by short black setae. Clypeus short, yellowish brown. Palpi pale, bearing both light and dark setae. Length ratio of palpal segments 3–5: 3:4=1.2, 4:5=0.67. Penultimate segment 2.25 times as long as wide, last segment 3.86 times as long as wide. Antennae dark brown, flagellomeres bearing dark sensilla that are shorter than width of respective flagellomere. First flagellomere widest apically, its length:width ratio 1.26 (width measured from the apex of the flagellomere). Other flagellomeres quadratic, slightly shorter than wide, except apical one that is elongated and bearing apical papilla; length:width ratios of fourth and last flagellomeres 0.9 and 1.91, respectively (Fig. 1b).

Scutum yellowish with three longitudinal brown stripes; median stripe consisting of two stripes that are largely merged, a narrow anterior gap between the stripes is present (Fig. 1b, c). Dark setae on scutum are present. Pleural sclerites of thorax light brown in colour, all bare except scutellum that has a dense row of setae along posterior margin. Halter light brown with dark setae.

Wings yellowish, with a faint subapical dark band extending from C to M2. Veins dark brown except bm–cu and bRs that are lighter. Veins R1 and bCuA with dorsal setae, R5 setose both ventrally and dorsally. Sc ending in C before bRs. R4 very short, about 0.12 times longer than apical portion of R5. Wing length 4.1 mm.

Coxae yellowish brown - brown, bearing short dark setae, legs yellowish. Ratio of femur to tibia for fore, mid and hind legs: 0.79, 0.68, 0.63. Ratio of tibia to basitarsus for fore, mid and hind legs: 1.67, 1.0, 1.0. Anterior spur of mid-tarsus about 0.5 times longer than posterior spur.

Abdominal tergites and sternites brown, bearing dark setae. Hypopygium brown. 9th tergite widest medially, apex rounded. Gonocoxites dorsally with an outgrowth, bearing a few long apical setae and having a mesial protrusion (Fig. 2a). Cerci prominent, club-like, apically setose, extending to the level of apices of gonostyli (Fig. 2a). Ventral lobe of gonostylus curved, its apical half mostly bare (Fig. 2a, b). Dorsal lobe of gonostylus elongated, pointed in dorsal view, bearing two black subapical long setae (Fig. 2a, b). Aedeagus curved ventrad in lateral view, apex blunt and medially widest in dorsal view (Fig. 2a, b, d). Parameres rod-like, apically dentate (Fig. 2c).

Female.The paratype female is lacking all legs except right fore leg and right hind femur. The specimen is slightly paler than the holotype male. The specimen may be somewhat teneral or it has bleached in the Malaise trap or later in the ethanol. Otherwise the specimen is very similar to the holotype. Antennal flagellomeres, except first and last, are wider than long (length:width ratio of 4th segment is 0.78). Cerci short, apically truncated, gonocoxite 8 short and rounded. Wing length 3.9 mm.

#### Diagnosis

The new species is characterised by the short and dark antennae, a yellow scutum with contrasting scutellar stripes, brown pleural sclerites of the thorax, brown, unicolorous abdomen and a short R4 vein. The dorsal lobe of gonostylus is strongly curved. The ventral lobe of gonostylus has only two black apical setae, while *O. nigricornis* (Fabricius) and *O. subnigricornis* Zaitzev & Menzel have a bunch of setae.

#### Etymology

The name of the new species refers to its putative boreo-alpine, disjunct range in Europe. The name is a noun in apposition.

#### Distribution

**Figure 3.**
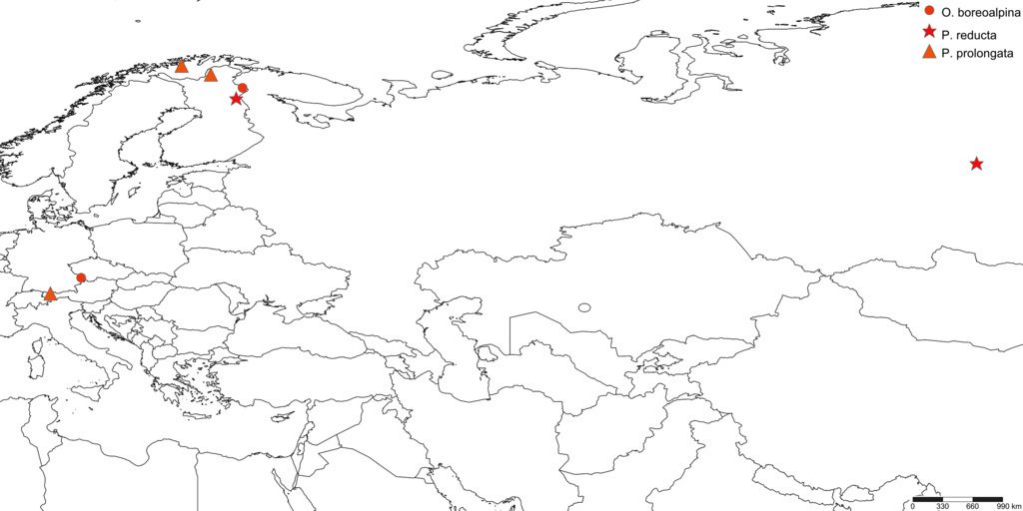
Collecting sites of the new fungus gnat species *Orfelia boreoalpina* Salmela sp.n., *Phronia reducta* Salmela sp.n. and *P. prolongata* Salmela sp.n.

The new species has been observed from eastern Finnish Lapland, the north boreal ecoregion, and from Germany, Bavaria (see Geiger et al. 2016). It is likely that *O. boreoalpina* sp.n. has a disjunct European range, having populations in the northern Fennoscandia and the Central European mountains (Fig. 3).

#### Ecology

The Finnish sampling site was a herb-rich meadow, harbouring vascular plants such as *Bistorta vivipara* and *Trollius europaeus*, and is probably flooded during snowmelt in spring. The meadow is surrounded by pine (*Pinus sylvestris*) dominated boreal forest. Bavarian site is a conifer-dominated mountain forest (Geiger et al. 2016)

#### Taxon discussion

The new species is rather distant to all other Holarctic species, but it may be closest to *O. nigricornis* and *O. subnigricornis* (see below). If using the key provided by Hutson et al. 1980, (species known from Great Britain), the species should either have a largely black or orange thorax, including the pleura, but *O. boreoalpina* sp.n. has an orange scutum and brown pleura, thus dropping out already in the first couplet. In the key provided by Zaitzev 1994 (Russian species), the new species keys in the first couplet (mesonotum yellow with broad longitudinal stripes). In the couplets 2–5 there are three options, and the new species comes closest to *O. nigricornis*, that has elongated palpal segments and one pointed outgrowth on the dorsal side of gonocoxites. *Orfelia nigricornis*, however, has a tuft of setae on the apex of dorsal lobe of the gonostylus while *O. boreoalpina* has only two dark setae. Other more or less similar species are 1) *O. subnigricornis*, that is characterized by the yellow scape and pedicel and median flagellomeres that are 1.4 times longer than wide (Zaitzev and Menzel 1996) (scape and pedicel dark and median flagellomeres about as long as wide in *O. boreoalpina* sp.n.; in addition dorsal lobe of the gonostylus in *O. subnigricornis* has a tuft of setae, only two setae are present in the new species), 2) *O. sachalinensis* (Matsumura) has a yellow abdomen and indistinct scutal stripes (see Okada 1938, as *Zelmira sachalinensis*) (*O. boreoalpina* sp.n. has a brown abdomen and strong scutal stripes) and 3) *O. minima* (Giglio-Tos) that has a yellowish scape, pedicel and a yellowish abdomen (Giglio-Tos 1890) (all dark in *O. boreoalpina* sp.n.).

#### DNA barcoding

Holotype male: BOLD Sample ID: DIPT-JS-2014-0233. BOLD Process ID: SCFI064-15. GenBank accession number: KY062990.

AACATTATATTTTATTTTAGGGACATGGTCAGGAATACTAGGAACATCAATAAGAATTTTAATTCGAGCAGAATTAGGATATCCGGGAGCATTAATTGGAAACGACCAAATTTATAATGTTGTAGTCACAGCTCATGCTTTTGTAATAATTTTTTTTATAGTTATACCTACTATAATTGGAGGTTTCGGAAATTGATTAGTACCTTTAATATTAGGGGCCCCAGATATGGCTTTTCCTCGAATAAATAACATAAGATTTTGACTTCTCCCTCCTTCACTTTCTTTACTATTAATAAGAAGAATAGTAGAAAGTGGTTCTGGAACAGGATGAACTGTATATCCTCCCCTATCTTCTACTTTATCTCATTCTGGTAGATCAGTTGACTTAACTATTTTTTCTCTTCATTTAGCAGGAATTTCTTCAATTCTTGGGGCAGTCAATTTTATTACTACAATTATCAACATACGATCACCTGGGATAAACATAGACATAATACCTTTATTTGTATGATCAGTTTTTATTACAGCCATTCTTCTTCTTTTATCATTACCTGTACTAGCGGGAGCAATTACAATACTTTTAACAGATCGTAATTTAAATACATCATTTTTTGATCCAGCAGGTGGGGGTGACCCAATTCTATATCAACATTTATTT

The DNA barcode of the paratype specimen is almost identical to the holotype, their similarity is 99.54 %. The type specimens belong to the same BIN (BOLD:ACJ7389) shared by no other members. The nearest specimens are rather distant: 97 closest sequences have similarity values between 88.25 and 86.35, being assigned to *O. nemoralis* (Meigen) (54 specimens), *O. nigricornis* (2), Keroplatidae (40) and Mycetophilidae (1). DNA barcode and associated data of the paratype is available from the BOLD Public data portal.

### Sciophila holopaineni

Salmela sp. n.

urn:lsid:zoobank.org:act:0E24E6EF-8C5A-4729-904C-2CB309E149C4

#### Materials

- **Type status:** Holotype. **Occurrence:** catalogNumber: DIPT-JS-2015-0075; recordedBy: J. Salmela; individualCount: 1; sex: male; **Location:** country: Finland; stateProvince: Lapponia kemensis pars orientalis; verbatimLocality: Törmäoja Conservation Area, Hannu Ollin vaara; verbatimLatitude: 67.843; verbatimLongitude: 29.468; verbatimCoordinateSystem: decimal degrees; verbatimSRS: WGS84; **Identification:** identifiedBy: J. Salmela; **Event:** samplingProtocol: Malaise trap; eventDate: 2013-7-8/9-19; habitat: old-growth boreal forest, dominated by birch (*Betula* sp.); **Record Level:** institutionCode: ZMUT
- **Type status:** Paratype. **Occurrence:** recordedBy: A. Polevoi; individualCount: 1; sex: male; **Location:** country: Russia; stateProvince: Karelia; verbatimLocality: 2 km NW of Syrovatka island; verbatimLatitude: 65.528; verbatimLongitude: 34.729; verbatimCoordinateSystem: decimal degrees; verbatimSRS: WGS84; **Identification:** identifiedBy: J. Salmela; **Event:** samplingProtocol: Malaise trap; eventDate: 2003-7-20/22; habitat: sea-shore meadow, close to a forest margin; **Record Level:** institutionCode: ZIN

#### Description

Male. Head black. Ocelli arranged in a row, on the posterior part of vertex; ratio of distance of lateral ocellus from median ocellus: distance of lateral ocelli from eye = 0.52. Vertex, anterior part of face and clypeus covered by dark setae. Eyes pubescent. Palpi infuscated, with dark setae. Length ratio of palpal segments 3–5: 3:4=0.94, 4:5=0.43. Penultimate segment 3.4 times as long as wide, last segment 9.3 times as long as wide. Antennae 16-segmented (scape, pedicel and 14 flagellomeres), black. Scape:pedicel length ratio 1.30; scape with a rounded, a bit depressed sensory field in its lateral base, having 7 minute setae. Flagellomeres cylindrical, length:width ratio of 1st flagellomere 1.51, 4th flagellomere 1.76 and apical flagellomere 3.13. Flagellomeres covered by dense light setosity, setae slightly curved, their length shorter than width of respective flagellomere; polygon-like (reticulate) pattern present, especially so in apical flagellomeres.

Thorax black. Scutum covered by pale setae. Anepimeron bare, other sclerites setose. Scutellum with eight setae in a curved row. Halteres light brown with pale setae; apical part of stem and base of knob infuscated.

Wings hyaline, lamina covered by both macro and microtrichia. Base of Rs, R4 and r-m bare, other veins setose, veins light brown to dark brown. C exceeding tip of R5 25 % of the distance between R5 and M1. Sc2 situated between base of Rs and R4. Furcation point of median fork at the level of bRs. M1+M2 very short. Length ratio of M1+2:r-m = 0.53. Wing length 3.2 mm.

Fore coxae light brown, mid and hind coxae dark brown, with pale setae, trochanters dark-brown. Legs yellowish brown, femora basoventrally darkened; apices of mid and hind coxae infuscated, the latter more clearly so. Setae on femora mostly dark, tibial and tarsal setae dark. Length ratio of femur to tibia for fore, mid and hind legs: 0.93, 1.03, 0.89. Length ratio of tibia to basitarsus for fore, mid and hind legs: 1.36, 1.57, 1.89. Anteroapical depressed area of the fore tibia with two rows of pale setae, proximal row curved with ca. 17 setae and distal row almost straight with ca. 20 setae. Ratio of apical width of tibia:length of longest tibial spur for fore, mid and hind legs: 0.52, 0.33, 0.33.

Abdominal tergites and sternites dark brown - almost black, covered by dark setae. Distal margin of 9th tergite rounded (Fig. 4a). Gonocoxal apodemes shallow Y-shaped; apex of mesial branch rounded, and apex of proximal branch weakly pointed (Fig. 4b). Large median appendage of gonostylus with ca. 19 comb-like megasetae (Fig. 4d). Small median appendage of gonostylus with two or rarely three long setae (Fig. 4e). Ventral lobe of gonostylus with a highly prominent, elongated outgrowth; basally with two long setae (Fig. 4c, d, e). Aedeagus apically blunt, about as long as parameres. Parameres rather thin, apices contorted (Fig. 4b, f).

#### Diagnosis

This is a very dark species with the head, antennae, thorax, and abdomen black or dark brown. The 9th tergite is apically rounded. The ventral lobe of the gonostylus has a prominent apical outgrowth. The aedeagus is about as long as the parameres with the apex truncated. The parameres are rather thin with their apices contorted.

#### Etymology

The new species is named after Mr. Tuomas Holopainen, the founder, songwriter and keyboardist of a Finnish metal band, "Nightwish". The name is a genitive.

#### Distribution

**Figure 5.**
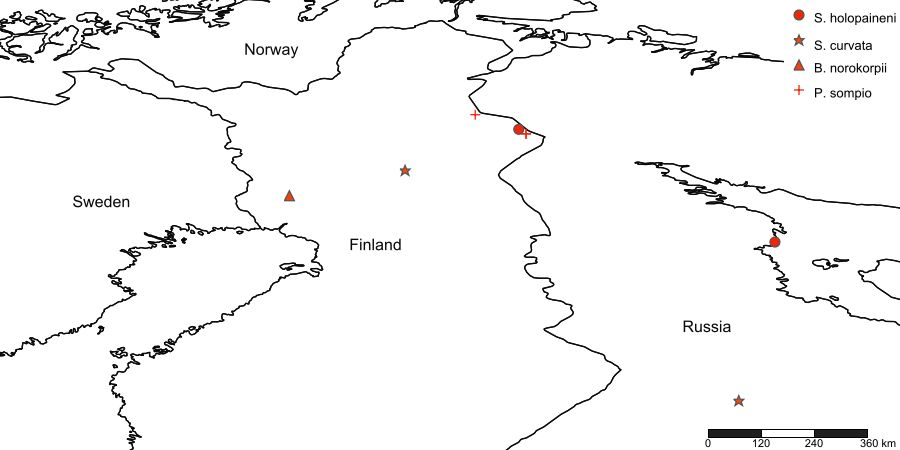
Collecting sites of new fungus gnat species *Sciophila holopaineni* Salmela sp.n., *Sciophila curvata* Salmela sp.n., *Boletina norokorpii* Salmela & Kolcsár sp.n. and *Phronia sompio* Salmela sp.n.

The new species is so far known only from eastern Finnish Lapland, the north boreal ecoregion (Fig. 5).

#### Ecology

The type locality in Törmäoja Conservation Area was a sloping birch forest in a river canyon, close to a spring brook.

#### Taxon discussion

The new species seems to be rather distant from the known Holarctic species of *Sciophila*. The number of large setae on the small median appendage of gonostylus is varying, it may be two or three, thus making the use of Zaitzev's (Zaitzev 1982) key problematic. If two setae, the new species comes closest to *S. impar* Johannsen, a species that shares some traits with the new species (e.g. smoothly rounded distal edge of 9th tergite, aedeagus with a blunt apex), but is otherwise very different, having e.g. a high number of comb-like megasetae on the large median lobe of the gonostylus (63–65 vs. 19 in *S. holopaineni* sp.n.). If three setae, the species runs to the couplet 79 and thereafter to 95, 99, 109 and finally to 114, but the new species does not fit either *S. kashmirensis* Zaitzev or *S. stackelbergi* Zaitzev. Holotype male had two and three setae, paratype male had two in both gonostyli.

We were not able to find any notes in the literature on the presence of a sensory field at the base of scape among *Sciophila*. JS checked a few specimens in his collection (JES), and the character was present in *S. buxtoni* Freeman, *S. curvata* sp.n., *Leptomorphus forcipatus* Landrock, *Polylepta borealis* Lundström and *Allocotocera pulchella* (Curtis), but it was absent among *Anaclileia dziedzickii* (Landrock). In *Acnemia trifida* Zaitzev there was an ventroapical sensory field at the scape, with hyaline cover. It is possible, that this trait is symplesiomorphic (an ancestral character or trait state shared by two or more taxa) amongst Sciophilinae and is lost in some genera.

### Sciophila curvata

Salmela sp. n.

urn:lsid:zoobank.org:act:1B97183D-C325-46CA-9E12-AA1E98678CFE

#### Materials

- **Type status:** Holotype. **Occurrence:** catalogNumber: DIPT-JS-2015-0252; recordedBy: J. Salmela; individualCount: 1; sex: male; **Location:** country: Finland; stateProvince: Ostrobothnia borealis pars borealis; verbatimLocality: Kemijärvi, Pyhä-Luosto National Park, Karhunotko; verbatimLatitude: 67.001; verbatimLongitude: 27.133; verbatimCoordinateSystem: decimal degrees; verbatimSRS: WGS84; **Identification:** identifiedBy: J. Salmela; **Event:** samplingProtocol: Malaise trap; eventDate: 2014-6-10/7-11; habitat: old-growth boreal forest with an intermittent brook; **Record Level:** institutionCode: ZMUT
- **Type status:** Other material. **Occurrence:** recordedBy: A. Polevoi; individualCount: 1; sex: male; **Location:** country: Russia; stateProvince: Karelia; verbatimLocality: Kivach Nature Reserve; verbatimLatitude: 62.272; verbatimLongitude: 33.986; verbatimCoordinateSystem: decimal degrees; verbatimSRS: WGS84; **Identification:** identifiedBy: J. Salmela; **Event:** samplingProtocol: Malaise trap; eventDate: 1990-8-13/9-11; habitat: Myrtillus pine forest; **Record Level:** institutionCode: FRIP

#### Description

Male. Head dark, almost black. Ocelli arranged in a shallow triangle, approximately on the median part of vertex; ratio of distance of lateral ocellus from median ocellus: distance of lateral ocelli from eye = 0.59. Vertex covered by dark setae, face covered by small setae and clypeus by longer setae. Eyes pubescent. Palpi pale, covered by pale setae. Length ratio of palpal segments 3–5: 3:4=0.81, 4:5=0.52. Penultimate segment 4.3 times as long as wide, last segment 11.0 times as long as wide. Antennae 16-segmented (scape, pedicel and 14 flagellomeres), brown, first flagellomere light brown. Scape:pedicel length ratio 1.38. Scape with a slightly depressed sensory field in its base, having 5-6 minute setae. Flagellomeres cylindrical, length:width ratio of 1st flagellomere 1.54, 4th flagellomere 1.3 and apical flagellomere 2.90. Flagellomeres covered by dense light setosity, setae slightly curved, their length shorter than width of respective flagellomere. Polygonal (reticulate) pattern not present in basal and median flagellomeres, and either unclearly present or absent on the apical flagellomeres; apical flagellomeres of the holotype are slightly wrinkled.

Thorax dark brown. Scutum covered by pale setae. Anepimeron bare, other sclerites setose. Scutellum with eight setae in a curved row. Halteres light brown with pale setae.

Wings hyaline, both macro and microtrichia present on lamina. Base of Rs and R4 bare, other veins setose, veins brown to dark brown. C exceeding tip of R5 22 % of the distance between R5 and M1. Sc2 situated above R4. Furcation point of median fork slightly before the level of R4. Length ratio of M1+2:r-m = 0.71. Wing length 2.6 mm

Coxae yellow, with pale setae, trochanters infuscated. Legs yellow, femora ventrobasally darkened, setae on femora pale, tibial and tarsal setae darker. Length ratio of femur to tibia for fore, mid and hind legs: 0.98, 0.92, 0.83. Length ratio of tibia to basitarsus for fore, mid and hind legs: 1.81, 1.64, 2.20. Anteroapical depressed area of the fore tibia with ca. 16 pale setae in a row. Ratio of apical width of tibia:length of longest tibial spur for fore, mid and hind legs: 0.65, 0.27, 0.26.

Abdominal tergites and sternites dark brown, covered by pale setae. 9th tergite triangular, apex pointed (Fig. 6a). Gonocoxal apodeme not prominent. Dorsal lobe of gonostylus narrow, finger-like, with a strong apical spine (Fig. 6b, c, e); large median appendage of gonostylus with 18 comb-like megasetae arranged in a two-serial row (Fig. 6d). Small median appendage of gonostylus with three long setae (Fig. 6d). Ventral lobe of gonostylus prominent, hump-backed in shape; two long basal setae not well separated from other setae of the lobe (Fig. 6c, d, e). Aedeagus bifid, about as long as parameres; parameres strongly curved (Fig. 6b, f).

#### Diagnosis

The new species is characterised by the presence of three setae on the small median lobe of the gonostylus, very narrow dorsal lobe of the gonostylus and strongly curved parameres. The new species is closest to *S. californiensis* Zaitzev; the 9th tergite of the latter species is medially constricted, in the former the outline of the 9th tergite is triangular.

#### Etymology

The name of the new species (*curvata* Latin, curved, an adjective) refers to the curved parameres of the male hypopygium.

#### Distribution

The type locality of the new species is from the Pyhä-Luosto National Park in central Finnish Lapland.

#### Ecology

The trapping site was a herb-rich bed of an intermittent brook, surrounded by an old-growth boreal forest.

#### Taxon discussion

The new species is most likely close to *S. californiensis*, because they share the following characters: the small median lobe of the gonostylus has three prominent setae and a narrow dorsal lobe. The new species, however, differs from *S. californiensis* by having a triangular 9th tergite (with a median constriction in *S. californiensis*) and having 18 comb-like megasetae (48 in *S. californiensis*). Although not mentioned in the description and improperly figured (Zaitzev 1982), parameres of *S. californiensis* seem to be rather long, while parameres of *S. curvata* sp.n. are strongly curved, not exceeding the apex of aedeagus.

### Boletina curta

Sasakawa & Kimura, 1974

urn:lsid:zoobank.org:act:1B97183D-C325-46CA-9E12-AA1E98678CFE

Boletina curta Sasakawa & Kimura, 1974: 60 (fig. 15a,b,c)
Boletina curta Zaitzev 1994: 209 (fig. 69,7)

#### Materials

- **Type status:** Paratype. **Occurrence:** recordedBy: M. Sasakawa; individualCount: 1; sex: male; **Location:** country: Japan; stateProvince: Honsu; verbatimLocality: Otsu, Mt. Hiei; verbatimLatitude: 35.06; verbatimLongitude: 135.83; verbatimCoordinateSystem: decimal degrees; verbatimSRS: WGS84; **Identification:** identifiedBy: J. Salmela; **Event:** eventDate: 1974-5-3; **Record Level:** institutionCode: OSAKA

#### Distribution

*Boletina curta* is a poorly known East Palaearctic species, hitherto recorded from Japan, Honshu (Sasakawa and Kimura 1974) and Russia, Sakhalin (Zaitzev 1994). *Boletina curta* is probably on the wing early in the season: collecting dates of the holotype and all paratypes (except Yoshino, the holotype of. *B. sasakawai* sp.n., see below) range between April 27 and May 3. However, Russian specimens reported by Zaitzev (1994) were collected in autumn (September 21).

#### Taxon discussion

*Boletina curta* was described from Honshu, the main island of Japan (Sasakawa and Kimura 1974). The type material consists of the holotype male, collected from Mt. Hiei, and paratypes collected from four other localities (in addition, an allotype female from a further site). Two of these paratypes were studied by the authors, and the paratype from the type locality was found to be conspecific with *B. curta*. This paratype, despite its explicit labeling, was not mentioned in the original description (Sasakawa and Kimura 1974). The second paratype, collected from Yoshino, does not fit to the concept of *B. curta*, and is here described as a new species (see below *B. sasakawai* sp.n.).

*Boletina curta* can be separated from the closely related *B. sasakawai* sp.n. and *B. norokorpii* sp.n. based on the following characters: 1) two stout setae present on the ventral lobe of the gonostylus (in the other taxa the ventral lobes of the gonostyli are bare, Fig. 7c), 2) the mid and hind femora are ventrobasally yellowish (infuscated in *B. sasakawai* sp.n.) and 3) the proximal row of stout setae on cerci (comb-like rows) is wider than apical row (Fig. 7b) (in the other taxa the rows are approximately equally wide, see Figs 8b, 9b). Furthermore, tibial spurs of *B. curta* are very dark (yellowish in other species) and the first flagellomere of *B. curta* is yellowish (becoming apically infuscated in *B. sasakawai* sp.n.); pedicel:first flagellomere length ratio of *B. curta* is 0.32 (0.24 in *B. sasakawai* sp.n. and 0.36 in *B. norokorpii* sp.n.). For the details of the male hypopygium of *B. curta*, please see Fig. 7.

Some *Boletina* species, such as *B. trivittata* Staeger, occur in both early and late season (J. Salmela, pers.obs.). Hence, it might be possible in theory that *B. sasakawai* sp.n. is just a late summer/autumn morph of *B. curta*, likewise the butterfly species *Araschnia levana* (Linnaeus), that has two distinct colour morphs within a season (see e.g. Ihalainen and Lindstedt 2012). *Boletina trivittata*, however, has overwintering adults, and so it is not truly bivoltine such as *A. levana*, that produces two adult generations within a summer. We assume that *B. sasakawai* sp.n. is not a late season morph of *B. curta*, because we found notable differences in the structure on male genitalia.

### Boletina sasakawai

Salmela & Kolcsár sp. n.

urn:lsid:zoobank.org:act:B5B58B8A-4527-41EB-BEAF-5290BEAE85D5

#### Materials

- **Type status:** Holotype. **Occurrence:** recordedBy: M. Sasakawa; individualCount: 1; sex: male; preparations: pinned specimen, glued to a card; **Location:** country: Japan; stateProvince: Honsu; verbatimLocality: Yoshino, Nara; verbatimLatitude: 34.68; verbatimLongitude: 135.83; verbatimCoordinateSystem: decimal degrees; verbatimSRS: WGS84; **Identification:** identifiedBy: J. Salmela; **Event:** eventDate: 1960-10-29; **Record Level:** institutionCode: OSAKA

#### Description

Male. Head black, vertex covered by pale setae, frons glabrous and face with scattered apical setae; face basally, close to scape, yellowish. Ocelli in a shallow triangle, median ocellus smallest. Clypeus with microtrichosity (pruinosity), elongated (about 1.7 times longer than basally wide). Scape yellowish and brownish, pedicel yellow and first flagellomere basally yellowish. Length ratio of pedicel:first flagellomere 0.24. Flagellomeres dark, palpus yellow.

Thorax dark-brown with pale setosity. Scutum shining, pleural sclerites with weak microtrichosity. Antepronotum yellow. Halter yellow. Femora yellow, bearing pale setae. Trochanters infuscated. Femora yellow, but mid and hind femora ventrobasally infuscated. Legs gradually darkening toward tarsi. Tibial spurs brownish. Length ratio of femur to tibia for fore, mid and hind legs: 0.77, 0.66, 0.66. Length ratio of tibia to basitarsus for fore, mid and hind legs: 1.06, 1.68, 1.68.

Apex of wing slightly infuscated. Bases of M1 and M2, M1+2, r-m, bM1+2, Rs, A1 and Sc bare, other veins setose. C exceeding tip of R5 36 % of the distance between R5 and M1. Sc ending in C at the level of Rs. Length ratio of M1+2:r-m = 1.19. Cu forking slightly beyond M end of r-m. Wing length 5.0 mm.

Abdomen dark-brown, tergites 2–4 laterodistally yellowish. 9th tergite elongated; cerci bearing two rows of combs, that are about equally wide, having ca. 45 stout setae (Fig. 8b). Ventral lobes of gonocoxites laterally rugose, basally with pale setosity, apices bare (Fig. 8a). Dorsal lobe of gonostylus with dense setosity, apical beak pointed, with minute setulae (Fig. 8c). Ventral lobe of gonostylus with no stout setae, sinuous (Fig. 8c). Apices of parameres horned (Fig. 8d, e).

#### Diagnosis

A large species with a vaguely infuscated wing apex, abdominal tergites 2–4 laterally yellowish and relatively long first flagellar segment (about 4-times the length of the pedicel). The ventral lobe of gonostylus bare, sinuous; in the closely related *B. curta* it is curved and bearing two stout setae. The apices of parameres with a conspicuous pair of horn-like outgrowths.

#### Etymology

The new species is named after Dr. Mitsuhiro Sasakawa, Japanese entomologist and the collector of the holotype. The name is a genitive.

#### Distribution

Known only from the type locality (Yoshino in Japan). The holotype male was collected at the end of October.

#### Taxon discussion

See above *Boletina curta*.

### Boletina norokorpii

Salmela & Kolcsár sp. n.

urn:lsid:zoobank.org:act:D906B0FD-0C14-4FDC-9FF7-EADF472C4476

#### Materials

- **Type status:** Holotype. **Occurrence:** catalogNumber: DIPT-JS-2016-0044; recordedBy: J. Salmela; individualCount: 1; sex: male; **Location:** country: Finland; stateProvince: Ostrobothnia borealis pars borealis; verbatimLocality: Ylitornio, Tuorerommas Mire Conservation Area; verbatimLatitude: 66.479; verbatimLongitude: 24.757; verbatimCoordinateSystem: decimal degrees; verbatimSRS: WGS84; **Identification:** identifiedBy: J. Salmela; **Event:** samplingProtocol: Malaise trap; eventDate: 2012-7-2/8-6; habitat: old-growth boreal forest with a spring brook; **Record Level:** institutionCode: ZMUT

#### Description

Male. Head black, vertex covered by pale setae, frons glabrous and face with scattered setae. Ocelli in a shallow triangle, median ocellus smallest. Clypeus not much longer than wide (about 1.2 times longer than basally wide). Scape and pedicel brownish, first and second flagellomeres yellowish, base of third flagellomere yellowish. Length ratio of pedicel:first flagellomere 0.36. Flagellomeres dark, palpus yellow.

Thorax dark-brown with pale setosity. Antepronotum yellow. Halter yellow. Femora yellow, bearing pale setae. Trochanters infuscated. Femora yellow. Legs gradually darkening toward tarsi. Tibial spurs brownish. Length ratio of femur to tibia for fore and hind legs: 0.93, 0.76. Length ratio of tibia to basitarsus of hind leg: 1.68.

Apex of wing slightly infuscated. Bases of M1 and M2, M1+2, r-m, bM1+2, Rs, A1 and Sc bare, other veins setose. C exceeding tip of R5 16 % of the distance between R5 and M1. Sc ending in C at the level of Rs. Sc2 present. Length ratio of M1+2:r-m = 1.14. Cu forking slightly beyond M end of r-m. Wing length 4.1 mm.

Abdomen dark-brown, tergites 2-4 laterodistally yellowish. 9th tergite elongated; cerci bearing two rows of combs, that are about equally wide, having 18 stout setae (Fig. 9b). Ventral lobes of gonocoxites laterally rugose, basally with pale setosity, apices bare (Fig. 9a). Dorsal lobe of gonostylus with setosity, apical beak relatively strong, pointed and bearing minute setulae (Fig. 9c). Ventral lobe of gonostylus with no stout setae, evenly curved in lateral view (Fig. 9c). Apices of parameres without horns (Fig. 9d, e).

#### Diagnosis

A species very close to *B. curta* and *B. sasakawai* sp.n. The ventral lobe of the gonostylus of *B. norokorpii* sp.n. is curved, having no stout setae (setae present in *B. curta*; ventral lobe of the gonostylus in *B. sasakawai* sp.n. is sinuous). The caudal and proximal combs of the cerci are equally wide, having relatively a small number (18) of stout setae (over 40 in both *B. curta* and *B. sasakawai* sp.n.)

#### Etymology

The new species is named after Dr. Yrjö Norokorpi, Finnish forest researcher and former area manager at Parks & Wildlife Finland. The name is a genitive.

#### Distribution

So far known from SW Finnish Lapland only (Fig. 5).

#### Ecology

The Finnish trapping site was an old-growth boreal forest characterised by vascular plants typical for base-rich soils, such as *Paris quadrifolia* and *Calypso bulbosa*.

#### Taxon discussion

The new species is very close to the eastern Palaearctic species *B. curta* and *B. sasakawai* sp.n. It is likely, however, that the eastern species are more related to each other than to *B. norokorpii* sp.n. For example, presence of Sc2 (absent in other species), shorter basal flagellar segments (1st flagellomere about 2.4 times longer than pedicel; in other species 3.1-4.2) and the small number (18; over 40 in other species) of stout setae of combs in the cerci separate the new species from the eastern Palaearctic taxa. We assume that both eastern Palaearctic species have restricted ranges in Japan, Far East Russia and neighbouring areas, whereas *B. norokorpii* sp.n. might have a widespread boreal range.

#### DNA barcoding

Holotype: BOLD Sample ID: DIPT-JS-2016-0044. BOLD Process ID: SCFI744-16. GenBank accession number: KY062991.

AATATTATATTTTATTTTTGGAGCTTGATCAGGAATAATTGGTACATCATTAAGAATTCTTATTCGTGCTGAATTAGGACACCCTGGAGCATTAATTGGAGATGATCAAATTTATAATGTTATTGTAACAGCTCATGCATTTGTAATAATTTTTTTTATAGTAATACCTATTATAATTGGAGGATTTGGTAATTGATTAATCCCTTTAATATTAGGAGCTCCTGATATAGCATTCCCTCGAATAAATAATATAAGATTTTGACTACTTCCTCCTTCATTAATATTACTTTTATCCAGAAGTTTAGTTGAAACAGGGGCTGGTACAGGTTGAACAGTGTACCCACCATTATCCTCAACAATTGCTCATGCAGGAGCATCTGTTGATTTAGCAATTTTTTCATTACATTTAGCAGGAATTTCTTCTATTTTAGGAGCTGTAAATTTTATTACTACAATTATTAATATACGAGCTCCTGGAATTACTTTTGAACGAATACCTCTTTTTGTATGATCAGTTTTAATTACAGCTATTTTATTATTATTATCTCTCCCAGTTTTAGCTGGAGCTATTACTATACTTTTAACAGACCGTAATTTAAATACATCATTTTTTGATCCTGCTGGAGGAGGAGATCCTATTTTATATCAACACTTATTC

The new species is assigned to the BIN BOLD:ADD1952, shared by no other specimens. In BOLD database the closest matches to this specimen are three *Boletina lundbecki* Lundström and four *Boletina* unassigned to taxonomic species (93,43 - 93,02 similarity).

### Phronia sompio

Salmela sp. n.

urn:lsid:zoobank.org:act:B3FBE538-6CFA-451B-AB4F-F62698CC3E27

#### Materials

- **Type status:** Holotype. **Occurrence:** catalogNumber: DIPT-JS-2014-0011; recordedBy: J. Salmela; individualCount: 1; sex: male; **Location:** country: Finland; stateProvince: Regio kuusamoensis; verbatimLocality: Salla, Värriö Strict Nature Reserve, Kuntasjoki; verbatimLatitude: 67.749; verbatimLongitude: 29.616; verbatimCoordinateSystem: decimal degrees; verbatimSRS: WGS84; **Identification:** identifiedBy: J. Salmela; **Event:** samplingProtocol: Malaise trap; eventDate: 2013-6-4/29; habitat: old-growth boreal riparian forest with seepages; **Record Level:** institutionCode: ZMUT
- **Type status:** Paratype. **Occurrence:** catalogNumber: DIPT-JS-2015-0101; recordedBy: J. Salmela; individualCount: 1; sex: male; **Location:** country: Finland; stateProvince: Regio kuusamoensis; verbatimLocality: Salla, Värriö Strict Nature Reserve, Kuntasjoki; verbatimLatitude: 67.750; verbatimLongitude: 29.620; verbatimCoordinateSystem: decimal degrees; verbatimSRS: WGS84; **Identification:** identifiedBy: J. Salmela; **Event:** samplingProtocol: Malaise trap; eventDate: 2013-6-29/7-29; habitat: riparian forest with lush vegetation and large amount of decaying trees,; **Record Level:** institutionCode: JES
- **Type status:** Paratype. **Occurrence:** catalogNumber: DIPT-JS- 2014-0404; recordedBy: J. Salmela; individualCount: 1; sex: male; **Location:** country: Finland; stateProvince: Lapponia kemensis pars orientalis; verbatimLocality: Savukoski, Urho Kekkonen National Park, Tyyroja; verbatimLatitude: 68.143; verbatimLongitude: 28.574; verbatimCoordinateSystem: decimal degrees; verbatimSRS: WGS84; **Identification:** identifiedBy: J. Salmela; **Event:** samplingProtocol: Malaise trap; eventDate: 2014-7-1/8-5; habitat: riparian meadow, spring brook with abundant Palustriella mosses; **Record Level:** institutionCode: JES

#### Description

Male. Head brown, vertex covered by pale setae, frons glabrous. Ocelli in a line, central ocellus smallest, lateral ocelli close to eyes, their distance from eye less than their own width. Eyes pubescent. Palpi brown, bearing light setae. Length ratio of palpal segments 3-5: 3:4=0.88, 4:5=0.61. Penultimate segment 2.62 times as long as wide, last segment 4.67 times as long as wide. Third palpomere with a sensory pit in its base. Antennae brown, 16-segmented (scape, pedicel and 14 flagellomeres), pedicel and base of first flagellomere yellowish brown. Scape:pedicel length ratio 1.47. Flagellomeres cylindrical, length:width ratio of 1st flagellomere 2.27, 4th flagellomere 1.67 and apical flagellomere 1.90. Flagellomeres covered by dense light setosity, setae slightly curved, their length shorter than width of respective flagellomere.

Thorax generally brown, except scutum that has yellowish brown anterior corners. Scutum with mainly pale setosity, two stout and long posterodorsal setae are present just above scutellum. Mediotergite bare, other sclerites bearing setae. Scutellum with four stout marginal setae. Halteres pale, bearing weak light setae and setulae.

Wings hyaline, veins light brown. Bases of M1 and M2, M1+2, base of r-m, bM1+2, bRs and Sc bare, other veins setose. C slightly exceeding tip of R5. Sc ending free. Length ratio of M1+2:r-m = 1.03. Wing length 1.8 mm.

Coxae yellow, bearing dark setae, legs yellowish. Length ratio of femur to tibia for fore, mid and hind legs: 1.02, 1.0, 0.84. Length ratio of tibia to basitarsus for fore, mid and hind legs: 1.28, 1.6, 1.63. Anteroapical depressed area of the fore tibia ovate, having ca. 20 light setae arranged in a curved row. Ratio of apical width of tibia:length of longest tibial spur for fore, mid and hind legs: 0.37, 0.30, 0.29.

Abdominal tergites and sternites brown, bearing light setae. 9th tergite and cerci without peculiar characteristics. Ventroapical margin of gonocoxite with a marked median emargination (Fig. 10b). Ventral lobe of gonostylus short, truncated, bearing two rather long subapical setae (Fig. 10b). Dorsal lobe of gonostylus widest apically, having 10 stout apical setae (Fig. 10a). Mesial portion of gonostylus relatively simple, having no comb-like structures (Fig. 10b, c, d, e); medially with a prominent, finger-like projection (1) and stemming from the same base a shorter projection (3), best visible in outer lateral view; these outgrowths are framed by a rounded, hyaline protrusion (2). Length:width ratio of aedeagal complex 1.14. Caudal margin of aedeagus notched, wide U-shaped, lateral apices, that may be parameres, appear bifurcated in ventral view (this is due to folding of the lateral apices). Median portion of aedeagus infuscated (Fig. 10a, f).

#### Diagnosis

A small species that is different from the known member of the genus. The male hypopygium has the following diagnostic characters: the ventroapical margin of the gonocoxites has a deep notch; the mesial projection of the gonostylus lacks comb-like structures but bears a prominent finger-like projection and a rounded, hyaline protrusion; the aedeagal complex is about as long as broad and is apically notched.

#### Etymology

The name of the new species refers to the old Forest Sami name of the region, Sompio, meaning large area bordered by aapamires. The biogeographical province of *Lapponia kemensis pars orientalis*, abbreviated as Lkor, is in Finnish "Sompion Lappi". The name is a noun in apposition.

#### Distribution

The species has been collected so far from three localities, all of these from eastern Finnish Lapland close to the Russian border. In fact, all of the collecting sites belong to the River Tuuloma catchment area east of the Maanselkä divide, so the waters finally flow to the Barents Sea in Russia.

#### Ecology

Collecting sites are small waterbodies (spring-fed headwater streams, spring brooks) surrounded by old-growth boreal forests.

#### Taxon discussion

The new species stands apart from all other Holarctic members of the genus.

#### DNA barcoding

BOLD Sample ID: DIPT-JS-2014-0011. BOLD Process ID: SCFI001-15.

BOLD Sample ID: DIPT-JS-2015-0101. BOLD Process ID: SCFI164-15.

BOLD Sample ID: DIPT-JS-2014-0404. BOLD Process ID: SCFI102-15.

Barcoding of the holotype and paratypes failed.

### Phronia reducta

Salmela sp. n.

urn:lsid:zoobank.org:act:082AAF64-6B68-438B-991A-9C42736838A3

#### Materials

- **Type status:** Holotype. **Occurrence:** catalogNumber: DIPT-JS-2015-0272; recordedBy: J. Salmela; individualCount: 1; sex: male; **Location:** country: Finland; stateProvince: Regio kuusamoensis; verbatimLocality: Salla, Iso Pyhätunturi; verbatimLatitude: 66.776; verbatimLongitude: 28.810; verbatimCoordinateSystem: decimal degrees; verbatimSRS: WGS84; **Identification:** identifiedBy: J. Salmela; **Event:** samplingProtocol: Malaise trap; eventDate: 2013-7-19/8-8; habitat: poor - intermediate rich sloping fen; **Record Level:** institutionCode: ZMUT
- **Type status:** Paratype. **Occurrence:** catalogNumber: 1386 (3); recordedBy: G.P. Ostroverkhova; individualCount: 1; sex: male; preparations: slide mounted; **Location:** country: Russia; stateProvince: Krasnoyarsk region; verbatimLocality: Tungussko-Chunsky District, village Vanavary; verbatimLatitude: 60.33; verbatimLongitude: 102.30; verbatimCoordinateSystem: decimal degrees; verbatimSRS: WGS84; **Identification:** identifiedBy: J. Salmela; **Event:** samplingProtocol: sweep net; eventDate: 1972-7-26; habitat: swampy forest; **Record Level:** institutionCode: TSU

#### Description

Male. Head dark-brown, vertex covered by pale setae, frons glabrous. Ocelli in a line, central ocellus slightly smaller than laterals; lateral ocelli close to eyes, their distance from eye less than their own width. Eyes pubescent. Palpi brown, bearing light setae. Length ratio of palpal segments 3–5: 3:4=0.83, 4:5=0.69. Penultimate segment 3.6 times as long as wide, last segment 5.3 times as long as wide. Third palpomere with a sensory pit in its base. Antennae brown, 16-segmented (scape, pedicel and 14 flagellomeres), base of pedicel and base of first flagellomere yellowish brown. Scape:pedicel length ratio 1.60. Flagellomeres cylindrical, length:width ratio of 1st flagellomere 2.86, 4th flagellomere 1.75 and apical flagellomere 3.0. Flagellomeres covered by dense light setosity, setae slightly curved, their length shorter than width of respective flagellomere.

Thorax generally dark-brown, except scutum that has yellowish anterior corners. Scutum with mainly pale setosity. Mediotergite bare, other sclerites bearing setae. Scutellum with four stout setae. Halteres pale, bearing weak light setae and setulae.

Wings hyaline, veins brown. Bases of M1 and M2, M1+2, base of r-m, bM1+2, base of Rs and Sc bare, other veins setose. C exceeds tip of R5 very slightly. Sc ending free. Length ratio of M1+2:r-m = 1.18. Wing length 3.1 mm.

Coxae yellow, bearing pale setae, legs yellowish, except femora ventrobasally and apices of hind femora infuscated. Length ratio of femur to tibia for fore, mid and hind legs: 0.95, 0.99, 0.82. Length ratio of tibia to basitarsus for fore, mid and hind legs: 1.03, 1.34, 1.60. Anteroapical depressed area of the fore tibia ovate, having ca. 19 light setae arranged in a slightly curved row. Ratio of apical width of tibia:length of longest tibial spur for fore, mid and hind legs: 0.39, 0.27, 0.24.

Abdominal tergites and sternites brown, bearing light setae. 9th tergite and cerci normal for the genus (Fig. 11a). Ventroapical margin of gonocoxites with a median notch (Fig. 11c). Gonostylus is intricate. Dorsal lobe of gonostylus lingulate, with numerous long setae on ventral margin (Fig. 11d, e). Mesial portion with a plate-like, inward projecting rows of combs (1) (Fig. 11d, e). Internal outgrowth of the ventral lobe of gonostylus is curved and apically notched (2) (Fig. 11e). The basal projection of the ventral lobe of gonostylus is relatively narrow and club-like (3) (Fig. 11d); median projection is the largest, bearing long basal setae and short subapical setae (4) (Fig. 11d, e). Aedeagus short and wide, parameres long, having no long apical setae, only small setulae are present (Fig. 11b, f).

#### Diagnosis

The new species is close of *P. braueri* Dziedzicki but differs in the following features; the apices of the parameres are non-setose (the setae here are long in *P. braueri*), the internal outgrowth of the ventral lobe of the gonostylus is curved and apically notched (not curved or notched in *P. braueri*), and the ventral lobe of the gonostylus also has a narrow club-like basal projection (wedge-shaped and widest basally in *P. braueri*).

#### Etymology

The name of the new species (Latin *reducta*, reduced, an adjective) is referring to the non-setose apices of the male parameres.

#### Distribution

Apparently a boreal species, hitherto known from NE Finnish Lapland and Siberia, Central Russia (Fig. 3).

#### Ecology

The species occurs in sloping fens and swampy forests. The Finnish collecting site (sloping fen) was close to a pine and spruce dominated pristine boreal forest.

#### Taxon discussion

**Figure 12.**
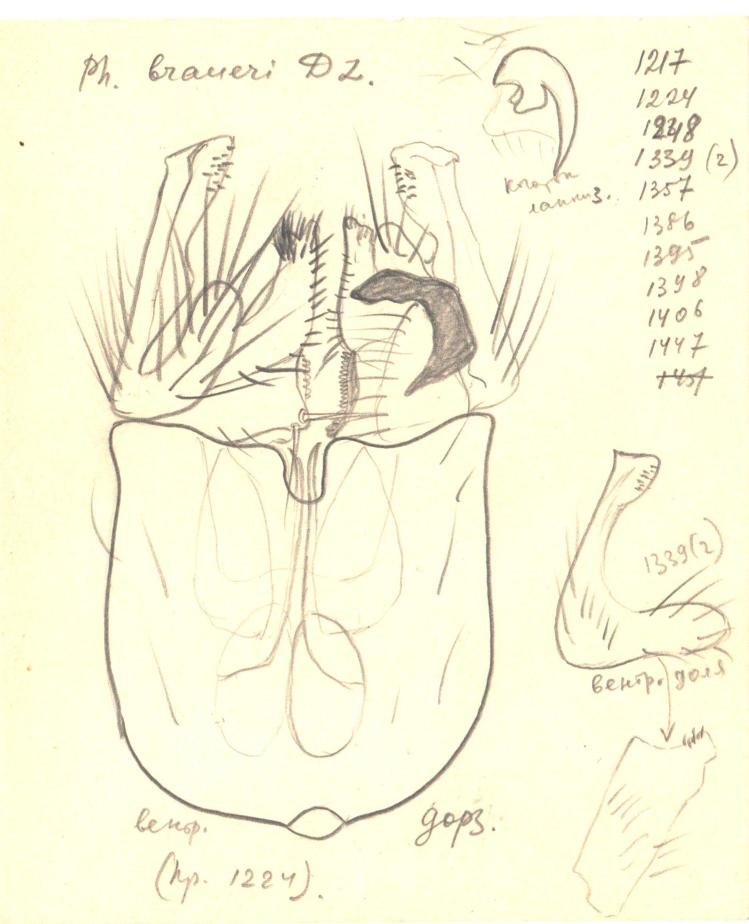
Original illustration of "*Phronia braueri*" (=*P. reducta* sp.n.) by G.P. Ostroverkhova. This illustration was published in Ostroverkhova 1979 and is reproduced here because the original publication is not easily available.

The new species was illustrated for the first time by Ostroverkhova 1979 (the original illustration is reproduced here, Fig. 12), as *P. annulata*, (= *P. braueri*). These two taxa are indeed closely related, but due to differences in the male hypopygia and DNA barcodes are considered as distinct species (see Diagnosis for details; comparative photos of *P. braueri* are provided in Fig. 13). There are a total of 10 slide-mounted “*Phronia braueri*” in TSU that were studied by Ostroverkhova, all of them collected from two close-lying localities, between dates 19.-29.7.1972. Unfortunately these slides are in poor condition making it difficult to identify them to species level; however the slide in the best condition was selected as the paratype.

There are two questionable older names of *P. braueri*, namely *P. annulata* Winnertz and *P. vittata* Winnertz (Winnertz 1863, Hackman et al. 1988), both are considered here as *nomina dubia*. These species are known from holotype females only and females of *P. braueri* are difficult to separate from *P. forcipata* Winnertz (Hackman 1970). It is also likely that the type specimens were destroyed during WWII (Kurina 2004, citing Evenhuis 1997). Furthermore, most likely the type specimens of both *P. annulata* and *P. vittata* were collected from Krefeld, Germany, that is a nemoral lowland area. We consider *P. reducta* sp.n. having a boreal range, being absent from Central Europe. Thus, we find it very unlikely that these *nomina dubia* would be conspecific with the new species.

#### DNA barcoding

Holotype male: BOLD Sample ID: DIPT-JS-2015-0272. BOLD Process ID: SCFI741-16. GenBank accession number: KY062992.

AATTTTATATTTTATTTTTGGAGCTTGATCTGGAATAGTGGGAACTTCTCTTAGAATTATTATTCGGACTGAATTAGGACATCCAGGAGCATTAATTGGTAATGACCAAATTTATAATGTTATTGTTACAGCTCATGCTTTTATTATAATTTTTTTTATAGTTATACCTATTATAATTGGAGGATTTGGAAATTGATTAGTTCCACTAATACTAGGAGCCCCTGATATAGCTTTTCCTCGAATAAATAATATAAGATTTTGGTTATTACCTCCTTCTCTTACATTATTACTTTCTAGAAGTTTAGTAGAAGCAGGGGCTGGAACTGGTTGAACAGTTTACCCTCCCCTTTCTTCAACTATTGCTCATGCTGGCGCATCAGTTGATTTAGCTATTTTTTCTTTACATTTAGCAGGTATTTCATCAATTTTAGGGGCAGTTAATTTTATTACTACCATTATTAATATACGAGCTCCTGGAATCACTTTTGATCGTTTACCTTTATTTGTTTGATCTGTTCTTATTACAGCAGTATTACTATTATTATCTTTACCCGTATTAGCAGGAGCTATTACTATACTATTAACAGACCGAAATCTTAATACTTCATTTTTTGACCCTGCAGGGGGAGGAGATCCTATTTTATACCAACATTTATTT

The holotype male is the only member of the BIN BOLD:ADD3565. This specimen has no very close matches in BOLD database. The closest matches are 44 *Phronia* specimens, whose similarities to the new species range between 96,74 - 96,01. One of these specimens is assigned to *P. braueri*, the sister species of *P. reducta* sp.n. That *P. braueri* specimen is collected from Norway and was identified by J. Kjaerandsen (unpublished record).

### Phronia prolongata

Salmela sp. n.

urn:lsid:zoobank.org:act:3A162039-F1B7-458E-BEBC-0317C31C25B7

#### Materials

- **Type status:** Holotype. **Occurrence:** catalogNumber: DIPT-JS-2015-0215; recordedBy: E. Rundgren; individualCount: 1; sex: male; **Location:** country: Finland; stateProvince: Lapponia inarensis; verbatimLocality: Inari, Muotkatunturi Wilderness Area, Kielajoki; verbatimLatitude: 69.146; verbatimLongitude: 26.292; verbatimCoordinateSystem: decimal degrees; verbatimSRS: WGS84; **Identification:** identifiedBy: J. Salmela; **Event:** samplingProtocol: Malaise trap; eventDate: 2014-6-26/8-5; habitat: lush and swampy riparian birch forest; **Record Level:** institutionCode: ZMUT
- **Type status:** Paratype. **Occurrence:** catalogNumber: MYCFI183-11; recordedBy: Finnmarksprosjektet; individualCount: 1; sex: male; **Location:** country: Norway; stateProvince: Finnmark; verbatimLocality: Alta, Goppaelva; verbatimLatitude: 70.027; verbatimLongitude: 23.394; verbatimCoordinateSystem: decimal degrees; verbatimSRS: WGS84; **Identification:** identifiedBy: J. Salmela, G. Söli; **Event:** samplingProtocol: sweep net; eventDate: 2010-6-13; **Record Level:** institutionCode: NHMO
- **Type status:** Paratype. **Occurrence:** catalogNumber: MYCFI184-11; recordedBy: Finnmarksprosjektet; individualCount: 1; sex: male; **Location:** country: Norway; stateProvince: Finnmark; verbatimLocality: Alta, Goppaelva; verbatimLatitude: 70.027; verbatimLongitude: 23.394; verbatimCoordinateSystem: decimal degrees; verbatimSRS: WGS84; **Identification:** identifiedBy: J. Salmela, G. Söli; **Event:** samplingProtocol: sweep net; eventDate: 2010-6-13; **Record Level:** institutionCode: NHMO
- **Type status:** Paratype. **Occurrence:** catalogNumber: BC-ZSM-DIP-22552-E10; recordedBy: D. Doczkal, S. Schmidt & J. Voith; individualCount: 1; sex: male; **Location:** country: Germany; stateProvince: Bavaria; verbatimLocality: Allgäu, Oberstdorf, Schochen; verbatimElevation: 2032 m; verbatimLatitude: 47.3936; verbatimLongitude: 10.3692; verbatimCoordinateSystem: decimal degrees; verbatimSRS: WGS84; **Identification:** identifiedBy: J. Salmela; **Event:** samplingProtocol: Malaise trap; eventDate: 2014-6-6/21; habitat: Blaugras-Horstseggenrasen; **Record Level:** institutionCode: ZSM
- **Type status:** Other material. **Occurrence:** catalogNumber: BIOUG21868-H06; recordedBy: BIObus; individualCount: 1; sex: female; **Location:** country: Canada; stateProvince: British Columbia; verbatimLocality: Vancouver Island; verbatimLatitude: 49.044; verbatimLongitude: -125.684; verbatimCoordinateSystem: decimal degrees; verbatimSRS: WGS84; **Identification:** identifiedBy: BOLD ID engine; **Event:** samplingProtocol: sweep net; eventDate: 2014-6-30; habitat: old growth temperate rain forest; **Record Level:** institutionCode: BIOUG

#### Description

Male. Head brown, vertex covered by pale setae, frons glabrous. Ocelli arranged in a very shallow triangle, central ocellus slightly smaller than laterals; lateral ocelli close to eyes, their distance from eye less than their own width. Eyes pubescent. Palpi brown, bearing light setae. Length ratio of palpal segments 3–5: 3:4=0.92, 4:5=0.62. Penultimate segment 3.51 times as long as wide, last segment 10.0 times as long as wide. Third palpomere with a sensory pit in its base. Antennae brown, 16-segmented (scape, pedicel and 14 flagellomeres). Scape:pedicel length ratio 1.28. Flagellomeres cylindrical, length:width ratio of 1st flagellomere 3.0, 4th flagellomere 2.58 and apical flagellomere 3.2. Flagellomeres covered by dense light setosity, setae slightly curved, their length shorter than width of respective flagellomere.

Thorax generally brown, scutum dorsally dark-brown. Scutum with mainly pale setosity, including the two stout and long dorso-posterior setae above scutellum. Mediotergite bare, other sclerites bearing setae. Scutellum with four stout marginal setae. Halteres pale, bearing weak light setae and setulae.

Wings hyaline, veins light brown. Bases of M1 and M2, M1+2, r-m, bM1+2, bRs and apex of Sc bare, other veins setose. C slightly exceeding tip of R5. Sc ending free. Length ratio of M1+2:r-m = 1.29. Wing length 3.2-3.5 mm.

Coxae and legs yellowish brown, bearing dark setae. Length ratio of femur to tibia for fore and mid legs (hind legs are missing from the holotype, ratios of that leg are form the German paratype): 0.93, 0.9, 0.76. Length ratio of tibia to basitarsus for fore and mid legs: 0.96, 1.21, 1.5. Anteroapical depressed area of the fore tibia ovate, having numerous light setae over the area. Ratio of apical width of tibia:length of longest tibial spur for fore and mid legs: 0.67, 0.33, 0.23.

Abdomen. 9th tergite and cerci as in Fig. 14a. Ventroapical projection of gonocoxites conspicuous, relatively long and narrow (Fig. 14c). Ventral lobe of gonostylus triangular, with a rather long and pointed ventrobasal outgrowth (Fig. 14d). Dorsal lobe of gonostylus relatively short, about 1.6 times longer than basally wide, bearing numerous setae (Fig. 14b, c, d). Mesial portion of gonostylus bearing 13–14 rows of combs, and a finger-like projection that is mostly bare, having an apical comb-row (Fig. 14d). Aedeagus (in lateral view) is evenly curved along its length and parameres are very long, about as long as aedeagus (Fig. 14e, f).

Female. In general, similar to male. Antennae dark except scape, pedicel and base of 1st flagellomere yellowish brown. Scape:pedicel length ratio 1.54. Length:width ratio of 1st flagellomere 3.1, 4th flagellomere 2.14 (apical flagellomeres broken off). Length ratio of M1+2:r-m = 1.58. Wing length 3.5 mm.

#### Diagnosis

**Figure 16.**
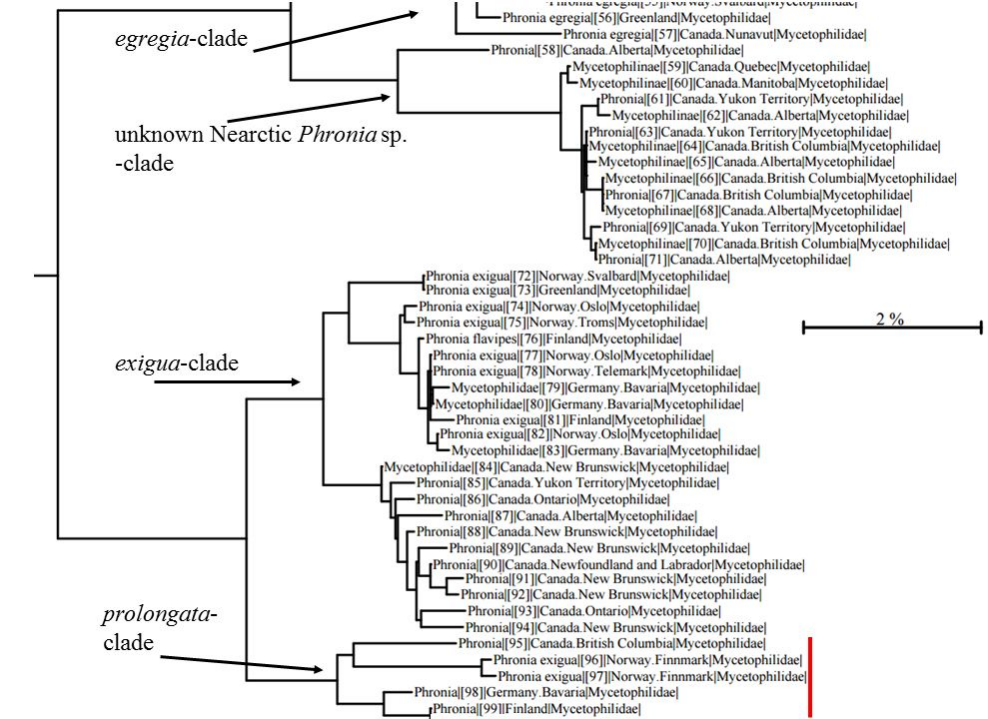
NJ-tree of *Phronia prolongata* sp.n. and related taxa based on COI (mtDNA) sequences and K2P distances (data from BOLD database). 95–98 represent *P. prolongata* sp.n. specimens studied here; the species displays a notable intraspecific variation of the barcoding gene COI, but all specimens are considered conspecific. The Canadian specimen is a female, and its identification is solely based on the COI sequence; Nearctic male specimens should be seen in order to validate the taxonomic assignment proposed here.

*Phronia prolongata* sp.n. is a closely related species of *P. exigua* (Zetterstedt, see Fig. 16). The ventroapical projection of the gonocoxites in the new species is rather long and narrow (shorter and broader in *P. exigua*), the aedeagus (in lateral view) is evenly curved along its length (less curved in *P. exigua*) and the parameres are very long, about as long as the aedeagus (much shorter in *P. exigua*, less than 0.5 times the length of the aedeagus).

#### Etymology

The name (Latin *prolongata*, an adjective) of the new species refers to the elongated, prolonged parameres of the male hypopygium.

#### Distribution

The new species has a Holarctic range, it is known from Canada (British Columbia), Norway, Finland and Germany (Fig. 3). Fennoscandian sites are located in the Arctic-Alpine ecoregion and the collecting site in Germany was at high altitude alpine zone.

#### Ecology

The Finnish collecting site was a swampy riparian birch forest and in Germany the species was collected from an alpine meadow.

#### Taxon discussion

The new species belongs to a group of species clastered with *P. exigua*, all sharing a beaked, setose ventral lobe of the gonostylus (ventrobasal outgrowth of the ventral lobe of gonostylus) and a row of ventral setae on the hind tibia (Gagné 1975). The new species is relatively close to *P. egregia* Dziedzicki, a species having very wide and apically expanded ventroapical lobe of the gonocoxite (see e.g. Gagné 1975, fig. 14 and Zaitzev 2003, fig. 93.2); this lobe in the new species is rather narrow and apically very slightly expanded (Fig. 14c). The closest relative of the new species is apparently *P. exigua*, that has a wide ventroapical lobe of the gonocoxite, the aedeagus is not evenly curved and the parameres are short (see Fig. 15; length of paramere:length of aedeagus 0.46, this ratio is 1.0 in *P. prolongata* sp.n.).

#### DNA barcoding

BOLD Sample ID: DIPT-JS-2015-0215. BOLD Process ID: SCFI251-15. GenBank accession number: KY062993.

AATTTTATACTTTATTTTTGGTGCTTGATCTGGAATAGTAGGAACTTCCCTAAGAATTATTATTCGTGCTGAACTTGGTCATCCAGGAGCATTGATTGGAAATGATCAAATTTATAATGTAATTGTTACTGCTCATGCTTTCATTATAATTTTTTTTATAGTTATGCCCATTATAATTGGTGGGTTTGGTAACTGACTTGTCCCATTGATATTGGGGGCCCCTGATATAGCTTTTCCTCGAATAAATAATATAAGTTTCTGATTATTGCCTCCCTCATTAACACTTCTTCTTTCAAGAAGTTTAGTCGAAGCTGGGGCTGGTACAGGTTGAACTGTTTATCCCCCTCTTTCTTCTACTATTGCTCACGCAGGATCTTCTGTTGATCTAGCTATTTTTTCTCTTCATTTAGCTGGTATTTCTTCAATTTTAGGGGCGGTAAATTTTATCACAACTATTATTAATATACGAGCTCCAGGAATTTCCTTTGATCGTTTACCTTTATTTGTTTGATCTGTTCTTATTACTGCTGTATTGCTTCTTTTATCGCTACCAGTTTTAGCTGGGGCTATTACTATACTTTTAACTGATCGAAATTTAAACACATCTTTCTTTGACCCTGCCGGAGGGGGGGACCCTATTCTTTATCAACATTTATTT

The similarity of COI sequences between the new species and *P. exigua* range between 95.57 and 94.8, and between the new species and *P. egregia* 89.98-87.86. The new species displays a notable intraspecific variation: the Canadian non-type female has 97.06 similarity compared to the holotype and the two Norwegian specimens have 96.6 similarity. Interestingly the similarity of the holotype and German paratype is 98.94, and these two are classified to the same Barcode index number (BIN) by the BOLD (BOLD:ACW2188), shared by no other specimens. The new species is, however, monophyletic (Fig. 16) and despite COI divergences, we find all the studied male specimens conspecific. For example, biting midges (Ceratopogonidae) *Brachypogon sociabilis* (Goetghebuer) and *Bezzia rhynchostylata* Remm in Finnmark, Norway, were characterised by relatively high intraspecific distances (4.0-3.8 %) and were classified to four and three BINs, respectively (Stur and Borkent 2014). Despite this variation, the specimens had no observable morphological differences and were considered conspecific. DNA barcode and associated data of the German paratype and Canadian female specimen are available from the BOLD Public data portal.

### Phronia elegantula

Hackman, 1970

urn:lsid:zoobank.org:act:FA7468D5-C149-46CB-9C0C-E67B51DF828F

Phronia elegantula Hackman 1970: 43 (figs. 10–13), description

#### Materials

- **Type status:** Holotype. **Occurrence:** recordedBy: A.V.V. Mikkola; individualCount: 1; sex: male; **Location:** country: Finland; stateProvince: Ostrobothnia kajanensis; verbatimLocality: Sotkamo, Aarreniemi; **Identification:** identifiedBy: W. Hackman; **Event:** eventDate: 1964-8-11; **Record Level:** institutionCode: MZHF
- **Type status:** Paratype. **Occurrence:** recordedBy: R. Tuomikoski, K. Mikkola; individualCount: 1; sex: male; **Location:** country: Finland; stateProvince: Regio kuusamoensis; verbatimLocality: Kuusamo, Juuma, Jäkälävuoma; **Identification:** identifiedBy: W. Hackman; **Event:** eventDate: 1964-8-21; **Record Level:** institutionCode: MZHF
- **Type status:** Other material. **Occurrence:** catalogNumber: DIPT-JS-2016-0166; recordedBy: J. Salmela; individualCount: 1; sex: male; **Location:** country: Finland; stateProvince: Lapponia kemensis pars orientalis; verbatimLocality: Pelkosenniemi, Luiron suot Mire Conservation Area, Sudenvaaranaapa; verbatimLatitude: 67.1900; verbatimLongitude: 27.6352; verbatimCoordinateSystem: decimal degrees; verbatimSRS: WGS84; **Identification:** identifiedBy: J. Salmela; **Event:** samplingProtocol: Malaise trap; eventDate: 2015-7-31/9-29; habitat: rich birch fen; **Record Level:** institutionCode: JES
- **Type status:** Other material. **Occurrence:** catalogNumber: DIPT-JS-2016-0167; recordedBy: J. Salmela; individualCount: 1; sex: male; **Location:** country: Finland; stateProvince: Lapponia kemensis pars orientalis; verbatimLocality: Pelkosenniemi, Luiron suot Mire Conservation Area, Sudenvaaranaapa; verbatimLatitude: 67.1900; verbatimLongitude: 27.6352; verbatimCoordinateSystem: decimal degrees; verbatimSRS: WGS84; **Identification:** identifiedBy: J. Salmela; **Event:** samplingProtocol: Malaise trap; eventDate: 2015-7-31/9-29; habitat: rich birch fen; **Record Level:** institutionCode: JES
- **Type status:** Other material. **Occurrence:** catalogNumber: NHMO_MYC00025; recordedBy: L.O. Hansen; individualCount: 1; sex: male; **Location:** country: Norway; stateProvince: Buskerud; verbatimLocality: Kongsberg, Skollenborg, Labro; verbatimLatitude: 59.6184; verbatimLongitude: 9.6774; verbatimCoordinateSystem: decimal degrees; verbatimSRS: WGS84; **Identification:** identifiedBy: J. Salmela; **Event:** samplingProtocol: sweep net; eventDate: 2008-9-28; **Record Level:** institutionCode: NHMO
- **Type status:** Other material. **Occurrence:** catalogNumber: NHMO_MYC00026; recordedBy: L.O. Hansen; individualCount: 1; sex: male; **Location:** country: Norway; stateProvince: Buskerud; verbatimLocality: Kongsberg, Skollenborg, Labro; verbatimLatitude: 59.6184; verbatimLongitude: 9.6774; verbatimCoordinateSystem: decimal degrees; verbatimSRS: WGS84; **Identification:** identifiedBy: J. Salmela; **Event:** samplingProtocol: sweep net; eventDate: 2008-9-28; **Record Level:** institutionCode: NHMO
- **Type status:** Other material. **Occurrence:** catalogNumber: BIOUG08254-E11; recordedBy: G. Sellmayer; individualCount: 1; sex: male; **Location:** country: Germany; stateProvince: Bavaria; verbatimLocality: Nationalpark Bayerischer Wald, 11.3 km N of Grafenau; verbatimElevation: 842 m; verbatimLatitude: 48.950; verbatimLongitude: 13.421; verbatimCoordinateSystem: decimal degrees; verbatimSRS: WGS84; **Identification:** identifiedBy: J. Salmela; **Event:** samplingProtocol: Malaise trap; eventDate: 2012-8-25/9-3; habitat: conifer-dominated mountain forest; **Record Level:** institutionCode: ZSM
- **Type status:** Other material. **Occurrence:** catalogNumber: BIOUG08259-G06; recordedBy: G. Sellmayer; individualCount: 1; sex: male; **Location:** country: Germany; stateProvince: Bavaria; verbatimLocality: Nationalpark Bayerischer Wald, 11.3 km N of Grafenau; verbatimElevation: 842 m; verbatimLatitude: 48.950; verbatimLongitude: 13.421; verbatimCoordinateSystem: decimal degrees; verbatimSRS: WGS84; **Identification:** identifiedBy: J. Salmela; **Event:** samplingProtocol: Malaise trap; eventDate: 2012-8-25/9-3; habitat: conifer-dominated mountain forest; **Record Level:** institutionCode: ZSM
- **Type status:** Other material. **Occurrence:** catalogNumber: BIOUG08318-G10; recordedBy: G. Sellmayer; individualCount: 1; sex: female; **Location:** country: Germany; stateProvince: Bavaria; verbatimLocality: Nationalpark Bayerischer Wald, 11.3 km N of Grafenau; verbatimElevation: 842 m; verbatimLatitude: 48.950; verbatimLongitude: 13.421; verbatimCoordinateSystem: decimal degrees; verbatimSRS: WGS84; **Identification:** identifiedBy: J. Salmela; **Event:** samplingProtocol: Malaise trap; eventDate: 2012-8-25/9-3; habitat: conifer-dominated mountain forest; **Record Level:** institutionCode: ZSM
- **Type status:** Other material. **Occurrence:** catalogNumber: BIOUG08251-F07; recordedBy: G. Sellmayer; individualCount: 1; sex: female; **Location:** country: Germany; stateProvince: Bavaria; verbatimLocality: Nationalpark Bayerischer Wald, 11.3 km N of Grafenau; verbatimElevation: 842 m; verbatimLatitude: 48.950; verbatimLongitude: 13.421; verbatimCoordinateSystem: decimal degrees; verbatimSRS: WGS84; **Identification:** identifiedBy: J. Salmela; **Event:** samplingProtocol: Malaise trap; eventDate: 2012-8-25/9-3; habitat: conifer-dominated mountain forest; **Record Level:** institutionCode: ZSM
- **Type status:** Other material. **Occurrence:** catalogNumber: BIOUG08217-B09; recordedBy: G. Sellmayer; individualCount: 1; sex: female; **Location:** country: Germany; stateProvince: Bavaria; verbatimLocality: Nationalpark Bayerischer Wald, 11.3 km N of Grafenau; verbatimElevation: 842 m; verbatimLatitude: 48.950; verbatimLongitude: 13.421; verbatimCoordinateSystem: decimal degrees; verbatimSRS: WGS84; **Identification:** identifiedBy: J. Salmela; **Event:** samplingProtocol: Malaise trap; eventDate: 2012-8-25/9-3; habitat: conifer-dominated mountain forest; **Record Level:** institutionCode: ZSM
- **Type status:** Other material. **Occurrence:** catalogNumber: BIOUG08218-G07; recordedBy: G. Sellmayer; individualCount: 1; sex: female; **Location:** country: Germany; stateProvince: Bavaria; verbatimLocality: Nationalpark Bayerischer Wald, 11.3 km N of Grafenau; verbatimElevation: 842 m; verbatimLatitude: 48.950; verbatimLongitude: 13.421; verbatimCoordinateSystem: decimal degrees; verbatimSRS: WGS84; **Identification:** identifiedBy: J. Salmela; **Event:** samplingProtocol: Malaise trap; eventDate: 2012-8-25/9-3; habitat: conifer-dominated mountain forest; **Record Level:** institutionCode: ZSM
- **Type status:** Other material. **Occurrence:** catalogNumber: BIOUG08217-C03; recordedBy: G. Sellmayer; individualCount: 1; sex: female; **Location:** country: Germany; stateProvince: Bavaria; verbatimLocality: Nationalpark Bayerischer Wald, 11.3 km N of Grafenau; verbatimElevation: 842 m; verbatimLatitude: 48.950; verbatimLongitude: 13.421; verbatimCoordinateSystem: decimal degrees; verbatimSRS: WGS84; **Identification:** identifiedBy: J. Salmela; **Event:** samplingProtocol: Malaise trap; eventDate: 2012-8-25/9-3; habitat: conifer-dominated mountain forest; **Record Level:** institutionCode: ZSM
- **Type status:** Other material. **Occurrence:** catalogNumber: BIOUG08211-A12; recordedBy: G. Sellmayer; individualCount: 1; sex: female; **Location:** country: Germany; stateProvince: Bavaria; verbatimLocality: Nationalpark Bayerischer Wald, 11.3 km N of Grafenau; verbatimElevation: 842 m; verbatimLatitude: 48.950; verbatimLongitude: 13.421; verbatimCoordinateSystem: decimal degrees; verbatimSRS: WGS84; **Identification:** identifiedBy: J. Salmela; **Event:** samplingProtocol: Malaise trap; eventDate: 2012-8-2/12; habitat: conifer-dominated mountain forest; **Record Level:** institutionCode: ZSM

#### Description

Male. Head dark-brown, vertex covered by dark setae, frons glabrous and face anteriorly with small setae. Ocelli arranged in a line, central ocellus smaller than laterals; lateral ocelli close to eyes, their distance from eye less than their own width. Eyes pubescent. Palpi yellowish-brown, bearing light setae. Length ratio of palpal segments 3–5: 3:4=0.98, 4:5=0.59. Penultimate segment 2.94 times as long as wide, last segment 8.2 times as long as wide. Third palpomere with a sensory pit in its base. Antennae brown, 16-segmented (scape, pedicel and 14 flagellomeres); scape, pedicel and basal half of first flagellomere yellowish. Scape with a prominent dorsal seta, about as long as first flagellomere. Scape:pedicel length ratio 1.33. Flagellomeres cylindrical, length:width ratio of 1st flagellomere 2.98, 4th flagellomere 1.75 and apical flagellomere 2.95. Flagellomeres covered by dense light setosity, setae slightly curved, their length shorter than width of respective flagellomere.

Thorax generally brown. Scutum dorsally with three dark stripes, that are almost confluent; the stripes are separated by very narrow yellowish gaps; anterolateral corners yellowish. Scutum with mainly pale setosity. Mediotergite bare, other sclerites bearing setae. Scutellum with four stout setae. Halteres pale, bearing weak light setae and setulae.

Wings hyaline, veins light brown. Bases of M1 and M2, M1+2, r-m, bM1+2, base of Rs and apex of Sc bare, other veins setose. C very slightly exceeding tip of R5. Sc ending free. Length ratio of M1+2:r-m = 1.29. Wing length 2.2 mm.

Coxae and legs yellow, apices of mid and hind femora sligthly infuscated, bearing dark setae. Length ratio of femur to tibia for fore, mid and hind legs: 0.99, 0.97, 0.79. Length ratio of tibia to basitarsus for fore, mid and hind legs: 1.08, 0.97, 0.8. Anteroapical depressed area of the fore tibia ovate, having ca. 20 light in a row. Ratio of apical width of tibia:length of longest tibial spur for fore, mid and hind legs: 0.35, 0.28, 0.25.

Abdomen mostly dark brown, but first, second and third tergites caudolaterally yellowish; these yellow areas are most extensive in second and third tergite. Sternum of second and third segments yellowish. Hypopygium dark brown. Ventroapical margin of gonocoxite with a wide and shallow median emargination, with a moderate median peak (Fig. 17b). Ventral lobe of gonostylus widest basally, rounded (Fig. 17a, c). Dorsal lobe of gonostylus rounded, widest subapically, having ca. 20 stout apical setae and four subapical setae that are thinner that apical setae (Fig. 17a, c). Mesial portion of gonostylus having a transversal, setose basal projection and above that two projections; the other one is simple and elongated, apically rounded, the other one is intricate, terminating into long and narrow projection (Fig. 17c). Inner lamina of the ventral lobe of gonostylus with medial a tuft of ca. eight setae, projecting perpendicularly from the lamina. Inner lamina basally, close to the edge of the stylus, with a larger group of setae. Comb-like structures are absent. Aedeagal complex rounded, length:width ratio 0.96. Aedeagal complex with a longitudinal sclerotised rod, that is basally divided into two apodemes and is apically anchor-shaped (Fig. 17d).

Female. Similar to male. Antennae dark except scape, pedicel and base of 1st flagellomere yellowish brown. Scape:pedicel length ratio 1.32. Length:width ratio of 1st flagellomere 3.9, 4th flagellomere 2.80, apical flagellomere 2.5. Length ratio of M1+2:r-m = 1.84. Wing length 2.2 mm.

#### Diagnosis

A *Phronia* species with a yellowish pattern on the abdominal tergites 1–3. The ventral lobe of the gonostylus is rounded and at its widest basally. The mesial projections are finger-like and the inner lamella of the ventral lobe of the gonostylus bears a tuft of setae. The species is somewhat close to *P. elegans* Dziedzicki and *P. signata* Winnertz, that have similarly shaped ventral lobe of the gonostylus; *P. elegantula* can be distinguished from these due to differences in the structure of the aedeagus, the ventral lobe of gonostylus and the mesial portion of the gonostylus.

#### Distribution

A European species. The species was described from eastern Finland (Ok: Sotkamo and Ks: Kuusamo) and has been later recorded from southern and northern parts of the country (J. Jakovlev, unpublished). The species has been found from Russian Karelia (Polevoi 2000) and Murmansk region (Polevoi 2010). It has a wide range in Sweden (Kjærandsen et al. 2007) and it has been once recorded from Germany, Bavaria (Plassmann 1980). The species is reported here for the first time from Norway; it may have a boreo-alpine disjunct range.

#### Ecology

Sampling sites are coniferous forests, mixed forests and wetlands.

#### Taxon discussion

*Phronia elegantula* is somewhat similar to *P. signata* and *P. elegans*, and has the same yellowish anterolateral corners to the scutum as well as a rotund ventral lobe of the gonostylus. However the abdomen of *P. elegans* is dark brown as opposed to some yellowish colouration on abdominal tergites 1–3 of *P. elegantula*. *Phronia signata* have only moderately emarginated ventroapical margins of the gonocoxites, whereas this character is much more conspicuous in *P. elegantula*. *Phronia signata* has ca. 14 setae on the ventral edge of the ventral lobe of gonostylus (see e.g. Dziedzicki 1889, fig. 8 and Zaitzev 2003, fig. 91.4), in *P. elegantula* these setae are absent.

#### DNA barcoding

BOLD Sample ID: DIPT-JS-2016-0166. BOLD Process ID: SCFI751-16. GenBank accession number: KY200862. BOLD Sample ID: DIPT-JS-2016-0167. BOLD Process ID: SCFI752-16. GenBank accession number: KY200863. The sequence provided here is from DIPT-JS-2016-0166.

TATTTTATATTTCATTTTTGGTGCTTGATCTGGTATAGTAGGTACTTCTTTAAGAATCATTATTCGAACAGAATTAGGACACCCTGGAGCCTTAATTGGAAATGATCAAATTTATAATGTTATTGTTACTGCTCACGCTTTTATTATAATTTTTTTTATAGTTATACCAATTATAATTGGAGGATTCGGTAATTGATTAGTTCCACTAATATTAGGAGCTCCAGATATAGCTTTCCCTCGAATAAATAATATAAGTTTTTGACTTTTACCACCATCTTTAACCTTATTACTTTCTAGTAGCTTAGTAGAAGCAGGGGCTGGAACAGGATGAACTGTTTATCCCCCTTTATCATCTACAATTGCCCATGCAGGAGCCTCAGTTGATTTAGCTATCTTTTCTTTACATTTAGCAGGTATTTCTTCTATTTTAGGAGCAGTAAATTTTATTACAACAATTATTAATATACGGGCCCCAGGAATTACTTTTGACCGAATACCATTATTTGTTTGATCGGTATTAATTACAGCAGTTCTTCTATTACTTTCTCTACCAGTTTTAGCTGGAGCTATTACTATATTATTAACAGATCGAAATTTAAATACCTCATTTTTTGACCCTGCCGGAGGAGGAGATCCCATTTTATATCAACACTTATTT

All studied specimens belong to the BIN BOLD:ACJ2889, and their similarities range between 99.69 and 98.78 (average 99.46). The nearest specimens in BOLD database belong to *P. disgrega* Dziedzicki, being 90.98 % similar to *P. elegantula*. DNA barcode and associated data of the German paratypes and female specimens is available from the BOLD Public data portal.

## Supplementary Material

XML Treatment for Orfelia boreoalpina

XML Treatment for Sciophila holopaineni

XML Treatment for Sciophila curvata

XML Treatment for Boletina curta

XML Treatment for Boletina sasakawai

XML Treatment for Boletina norokorpii

XML Treatment for Phronia sompio

XML Treatment for Phronia reducta

XML Treatment for Phronia prolongata

XML Treatment for Phronia elegantula

## Figures and Tables

**Figure 1a.**
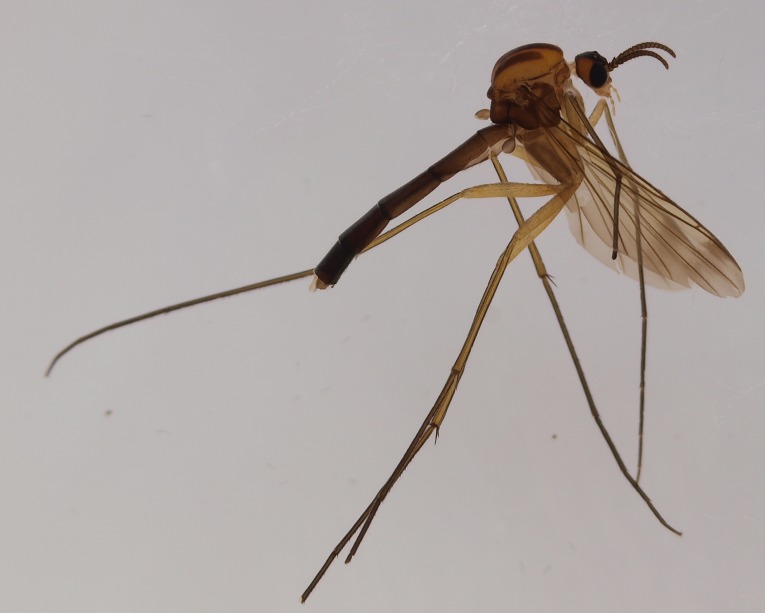
Habitus, lateral view.

**Figure 1b.**
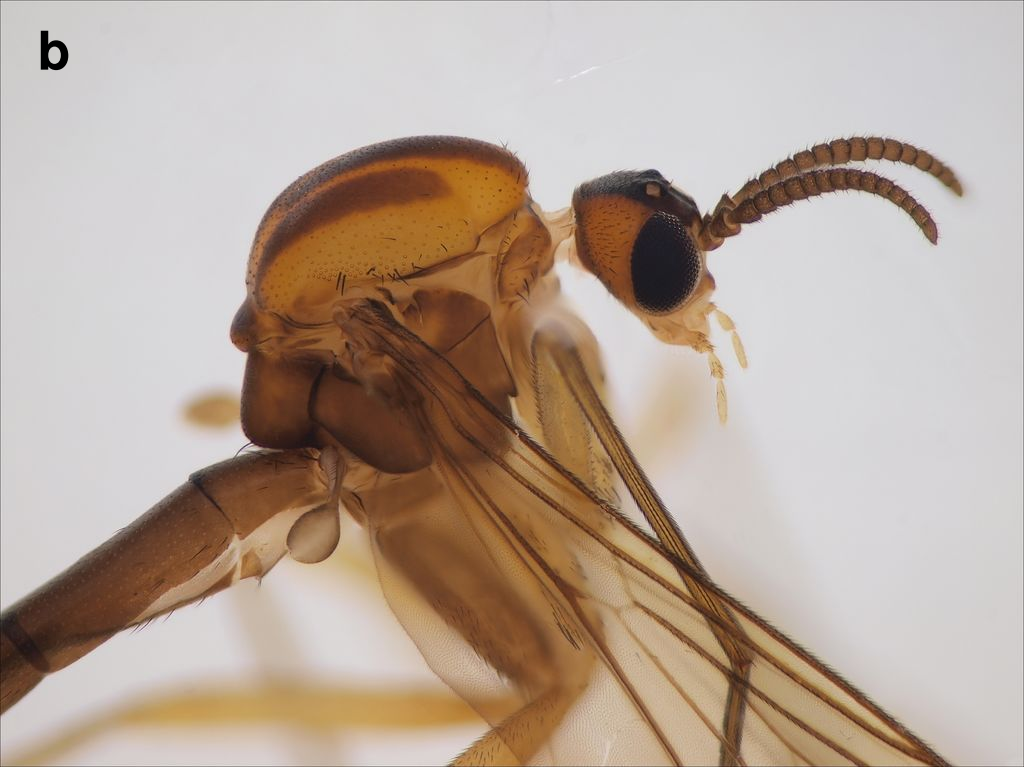
Thorax and head, lateral view.

**Figure 1c.**
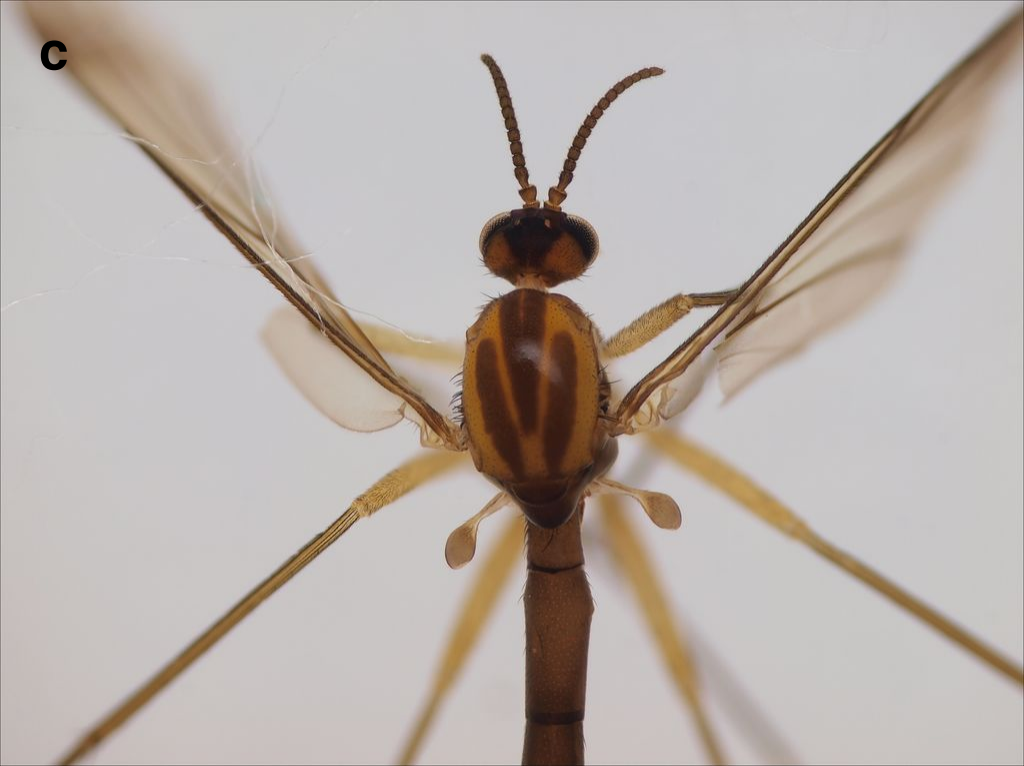
Thorax and head, dorsal view.

**Figure 2a.**
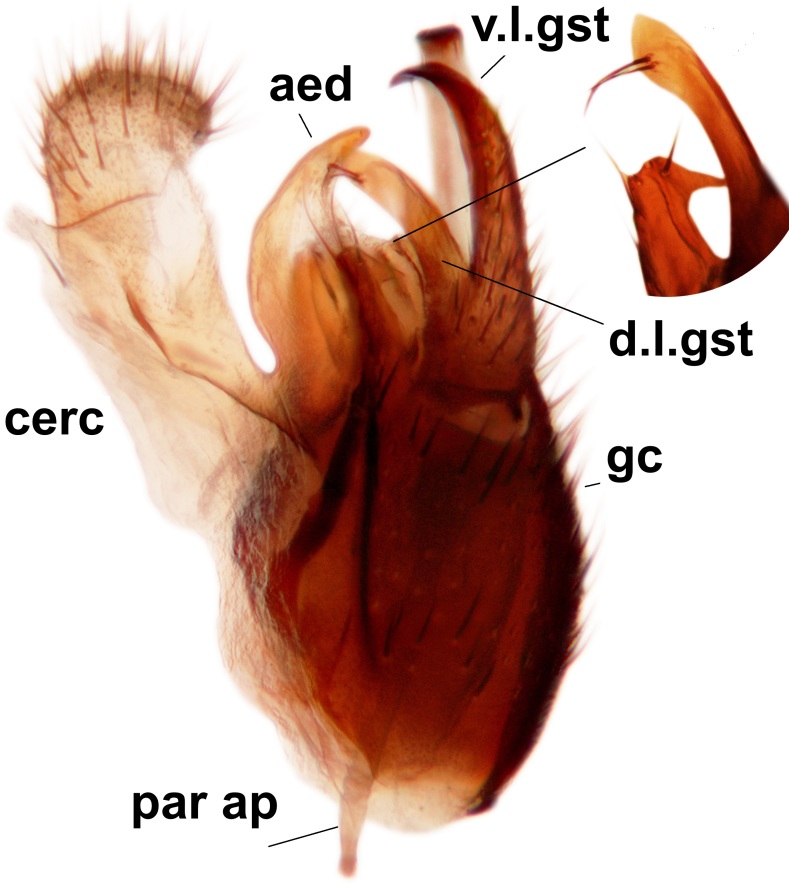
Hypopygium, lateral view. cerc=cerci, aed, aedeagus, gst=gonostylus, v.l.gst=ventral lobe of gst, d.l.gst=dorsal lobe of gst, gc=gonocoxite, par ap=parameral apodeme. Dorsal outgrowth of the gonocoxites is shown in the insert.

**Figure 2b.**
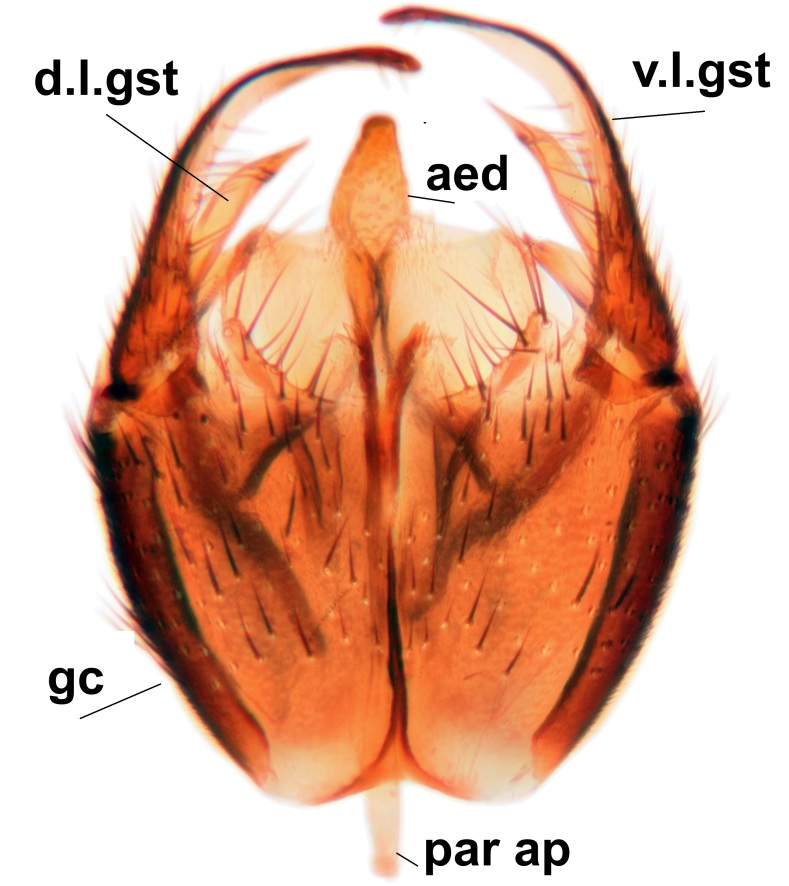
Hypopygium, ventral view.

**Figure 2c.**
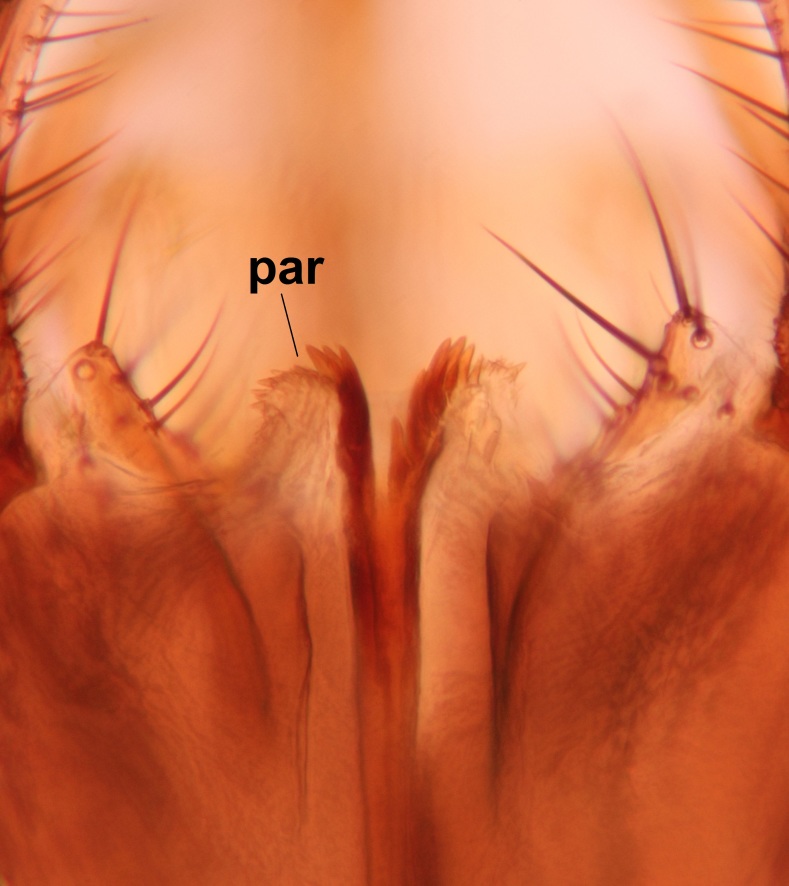
Parameres (par), ventral view.

**Figure 2d.**
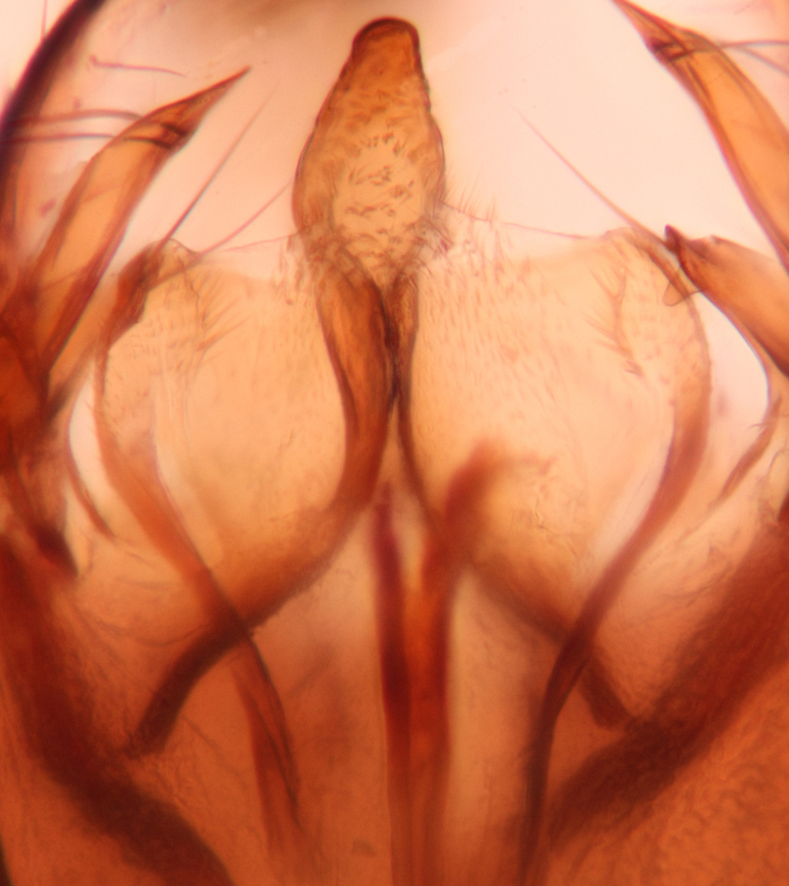
Aedeagus, ventral view.

**Figure 4a.**
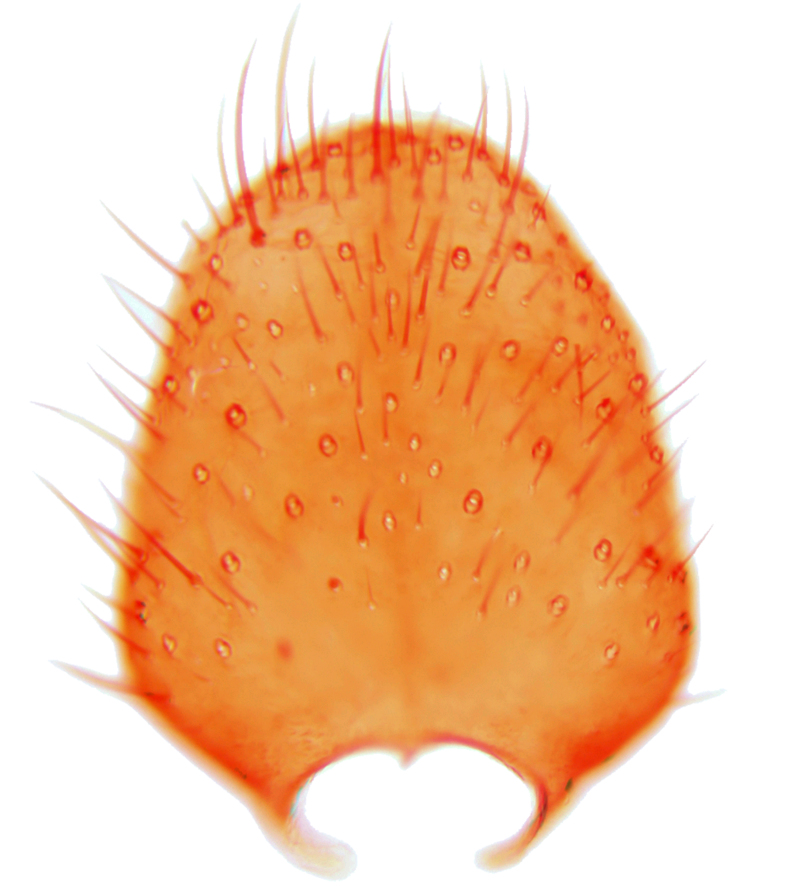
9th tergite, dorsal view.

**Figure 4b.**
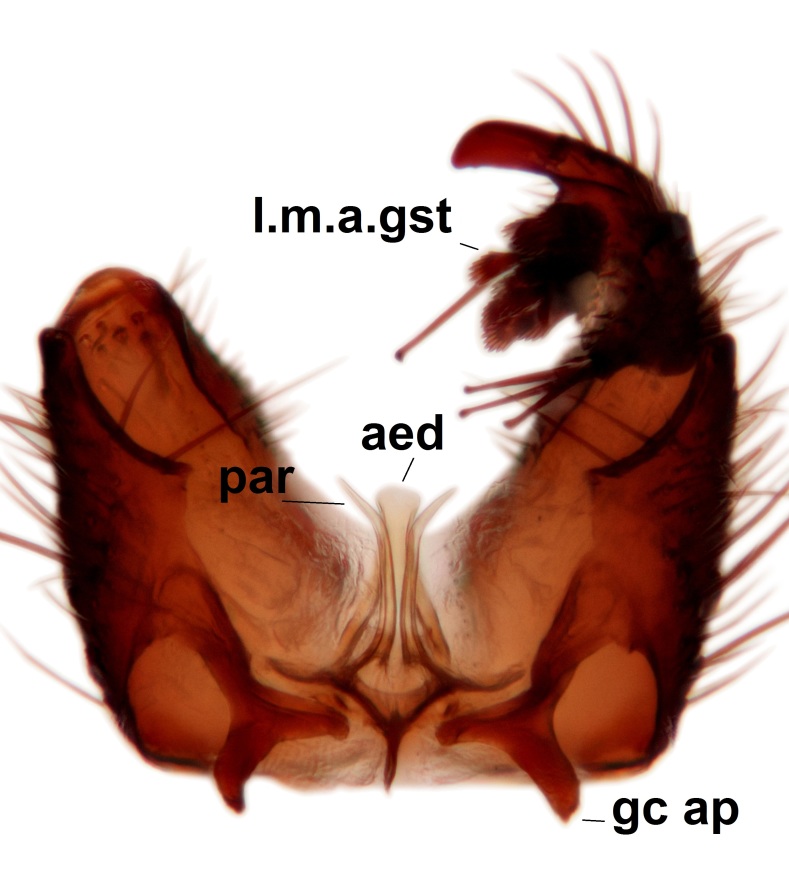
Hypopygium, dorsal view. gc=gonocoxite, gst=gonostylus, l.m.a.gst=large median appendage of gonostylus with comb-like megasetae, par=parameres, aed=aedeagus, gc ap=gonocoxal apodeme.

**Figure 4c.**
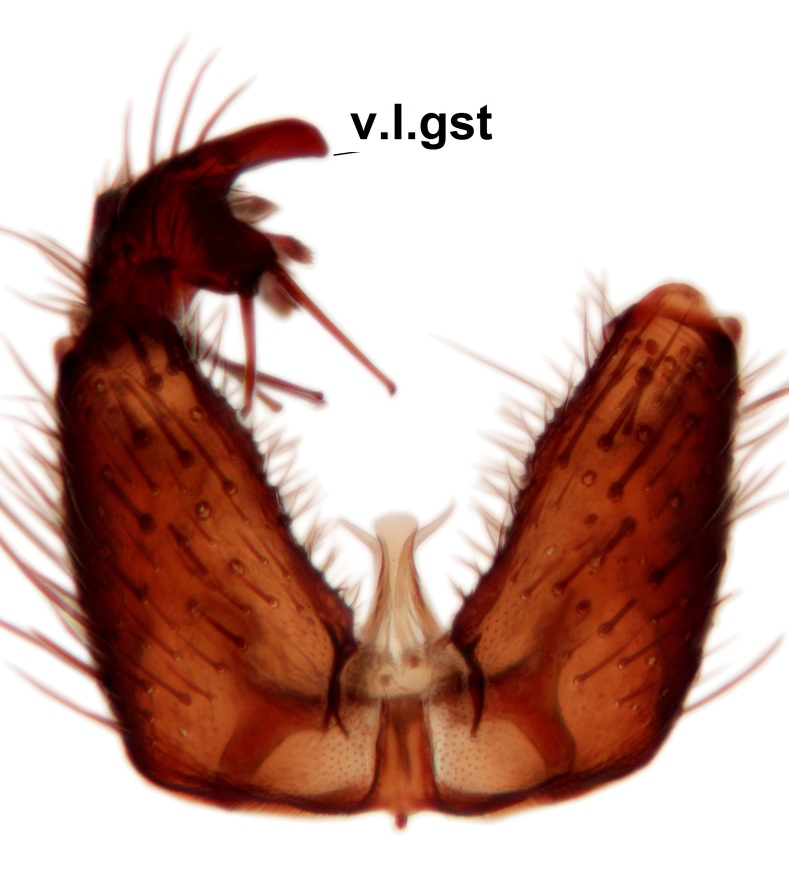
Hypopygium, ventral view. v.l.gst=ventral lobe of gst.

**Figure 4d.**
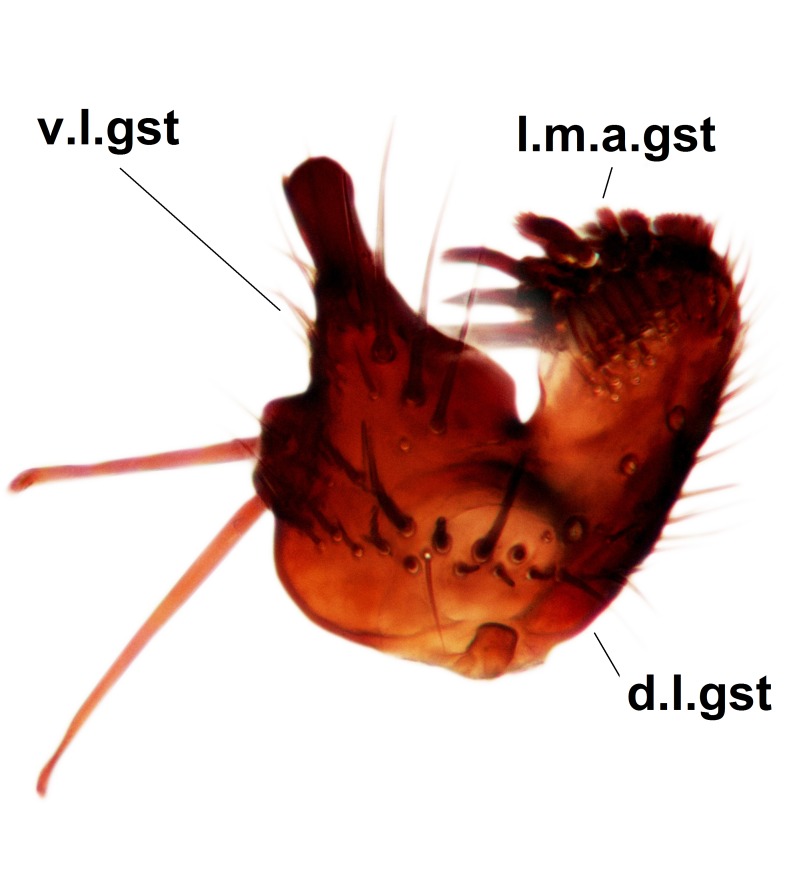
Gonostylus, outer lateral view. d.l.gst=dorsal lobe of gonostylus.

**Figure 4e.**
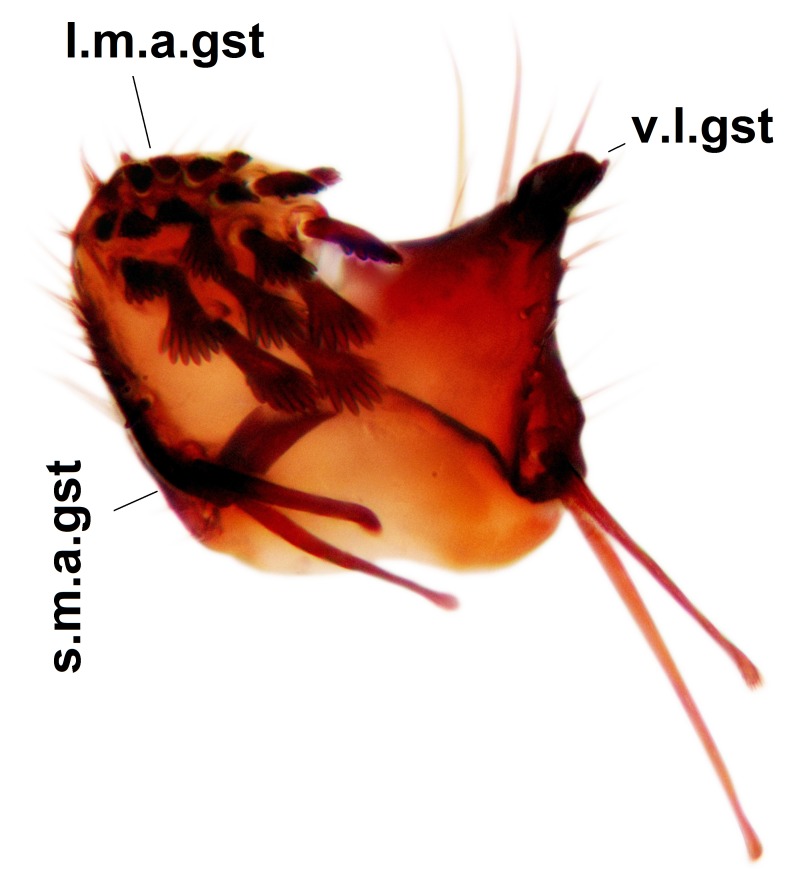
Gonostylus, inner lateral view. s.m.a.gst=small median appendage of gst.

**Figure 4f.**
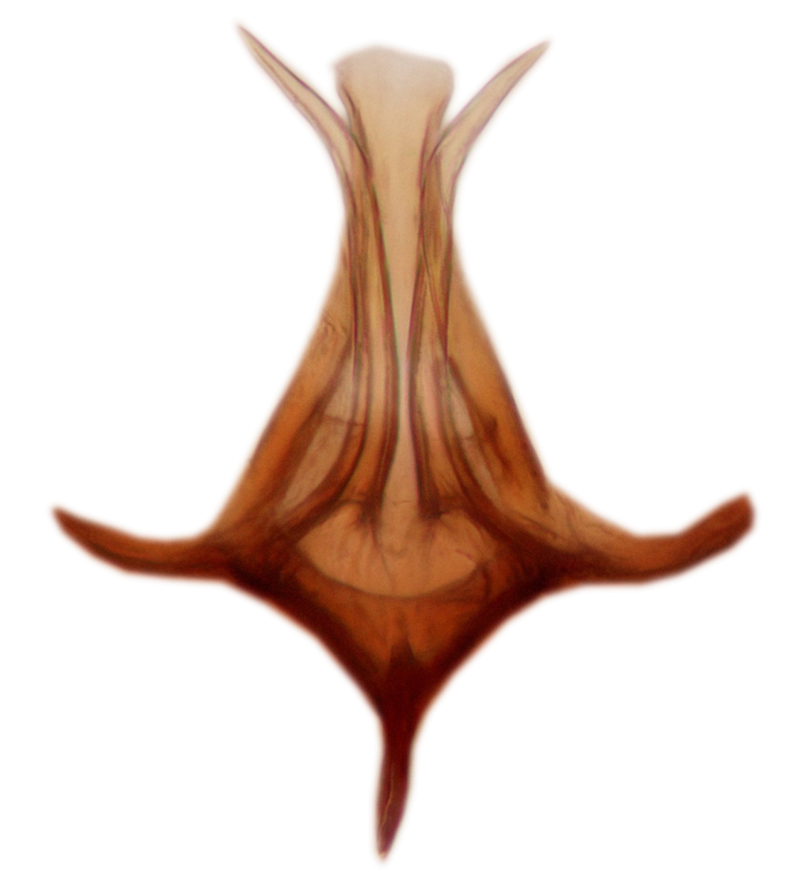
Aedeagus and parameres.

**Figure 6a.**
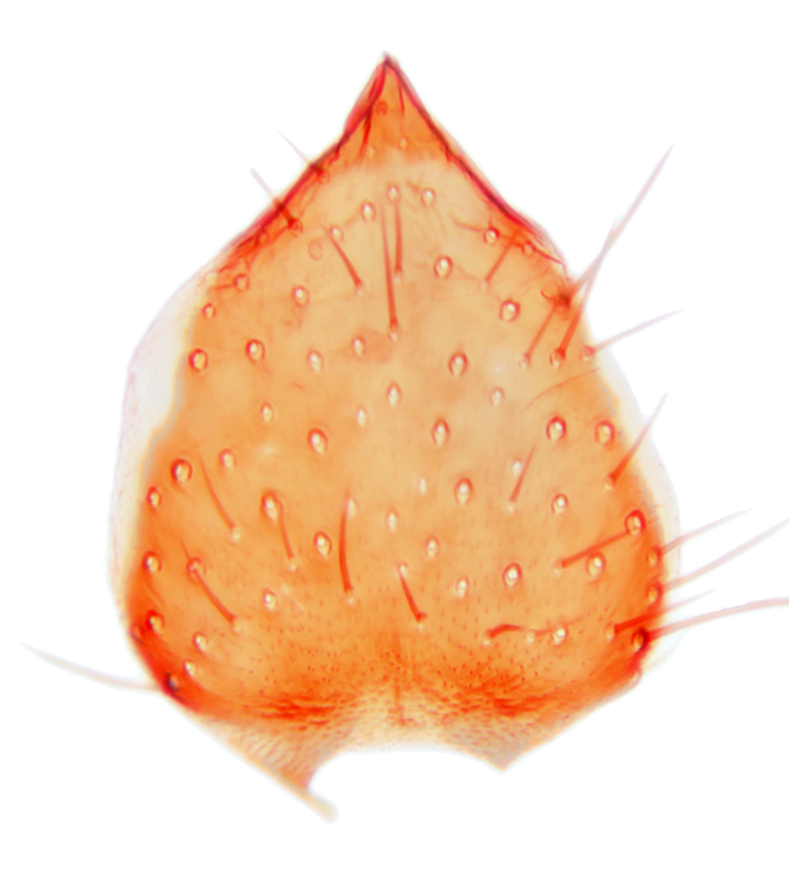
9th tergite, dorsal view.

**Figure 6b.**
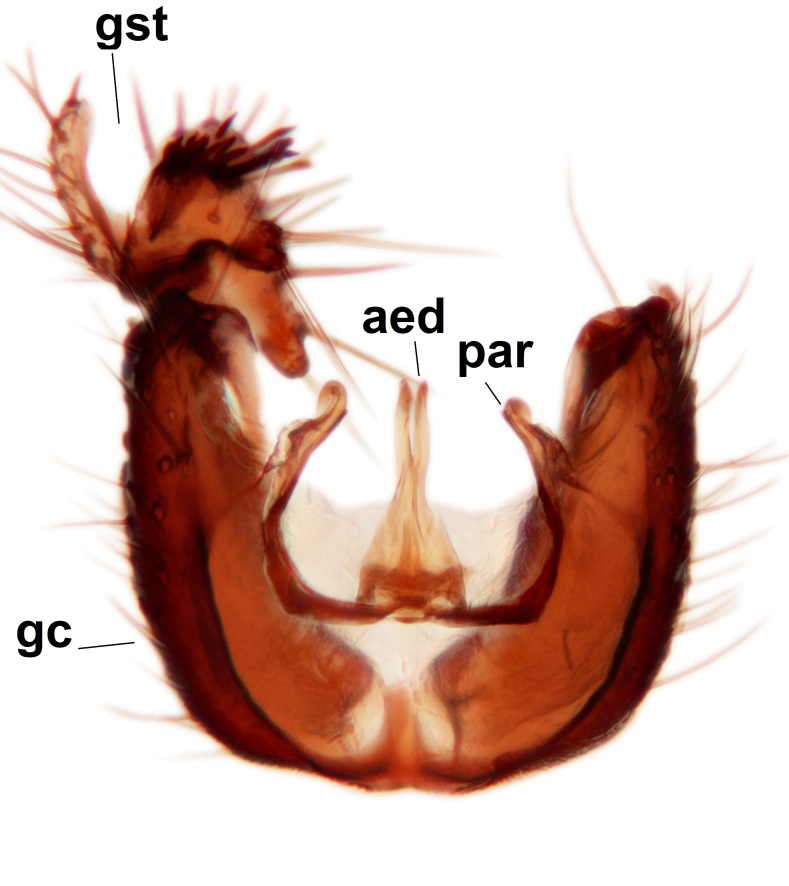
Hypopygium, dorsal view. gc=gonocoxites, gst=gonostylus, aed=aedeagus, par=parameres.

**Figure 6c.**
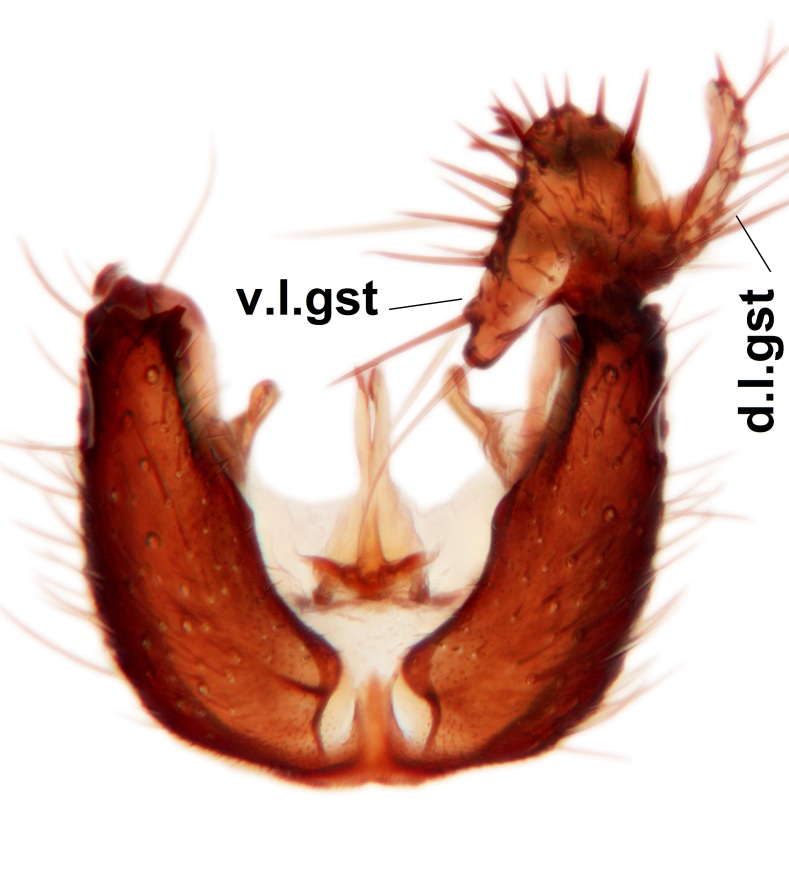
Hypopygium, ventral view. v.l.gst=ventral lobe of gonostylus, d.l.gst=dorsal lobe of gonostylus.

**Figure 6d.**
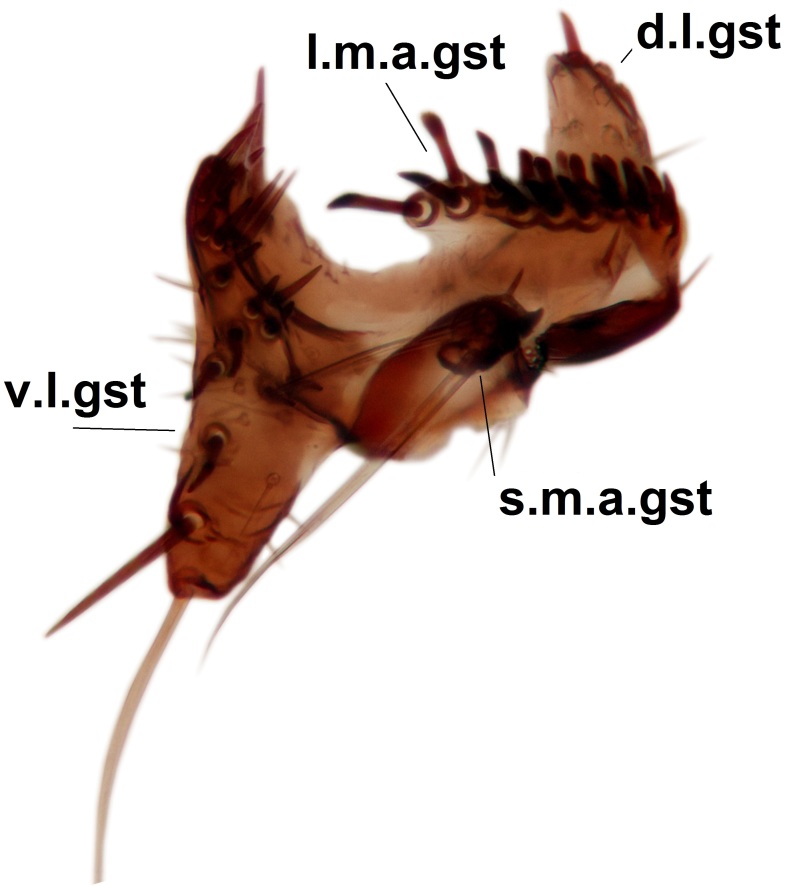
Gonostylus, inner lateral view. l.m.gst=large median appendage of gonostylus, s.m.a.gst=small median appendage of gst.

**Figure 6e.**
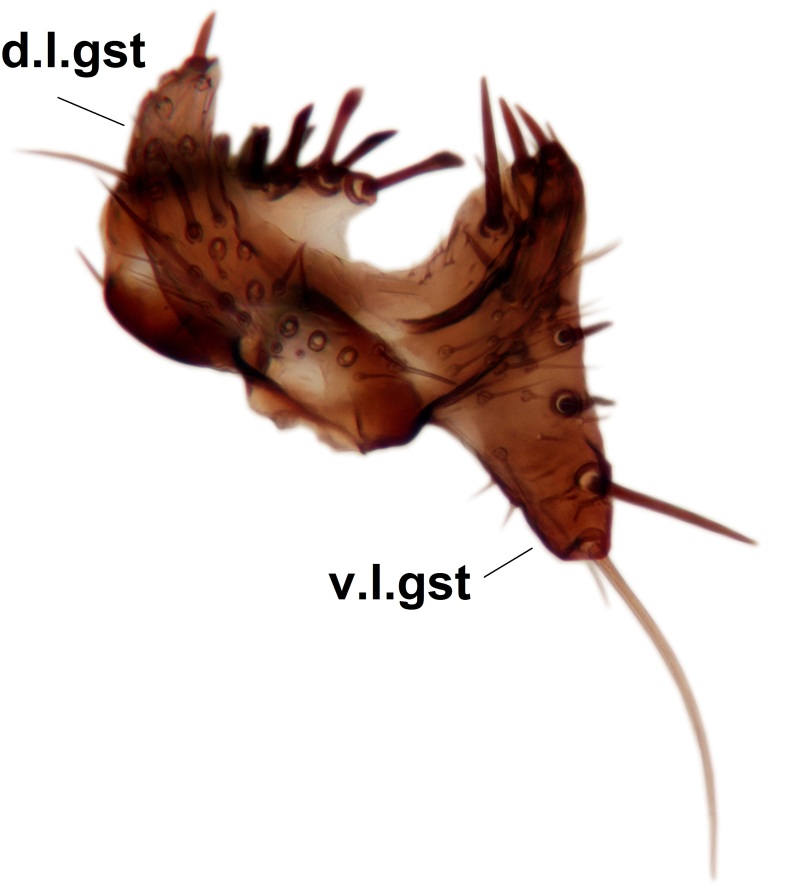
Gonostylus, outer lateral view.

**Figure 6f.**
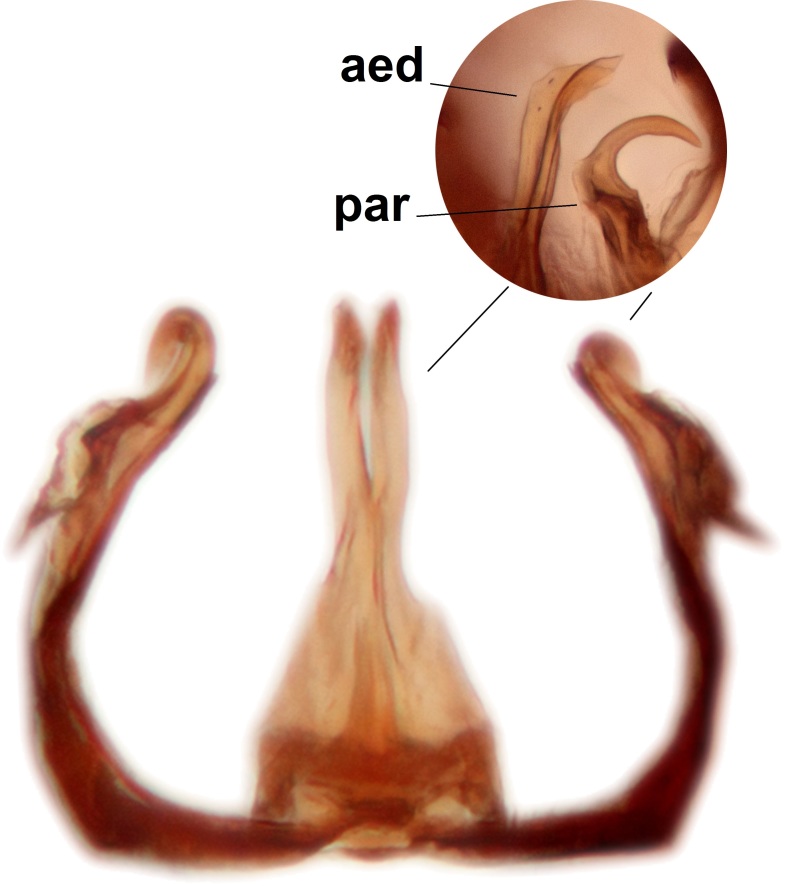
Aedeagus and parameres, dorsal view (insert shows lateral view on the apices of aedeagus and parameres).

**Figure 7a.**
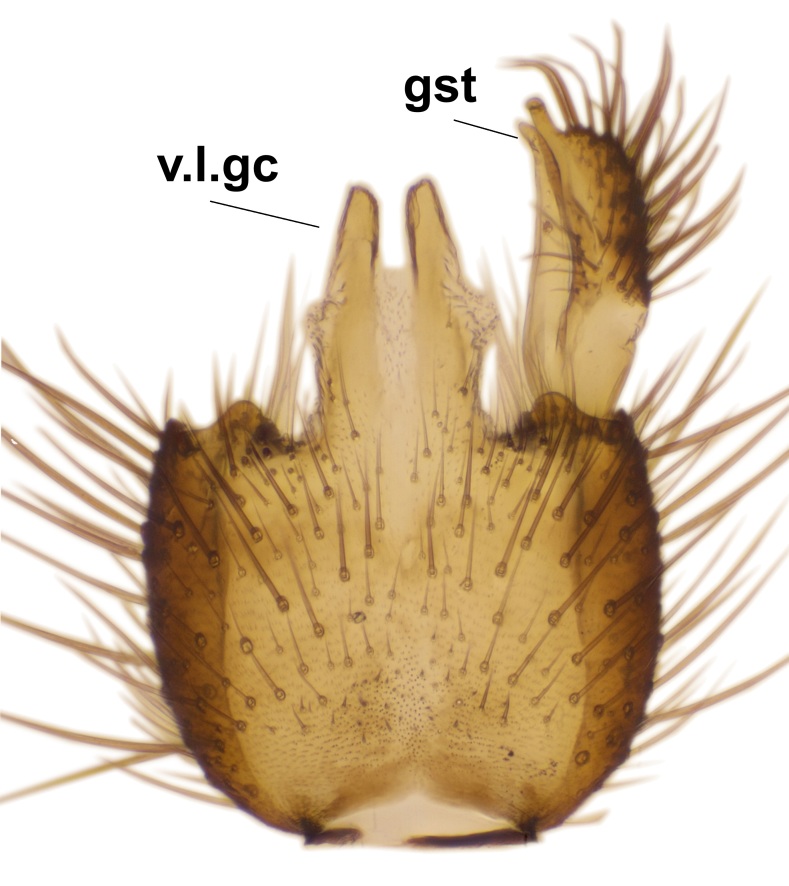
Hypopygium, ventral view. gst=gonostylus, v.l.gc=ventral lobes of gonocoxites.

**Figure 7b.**
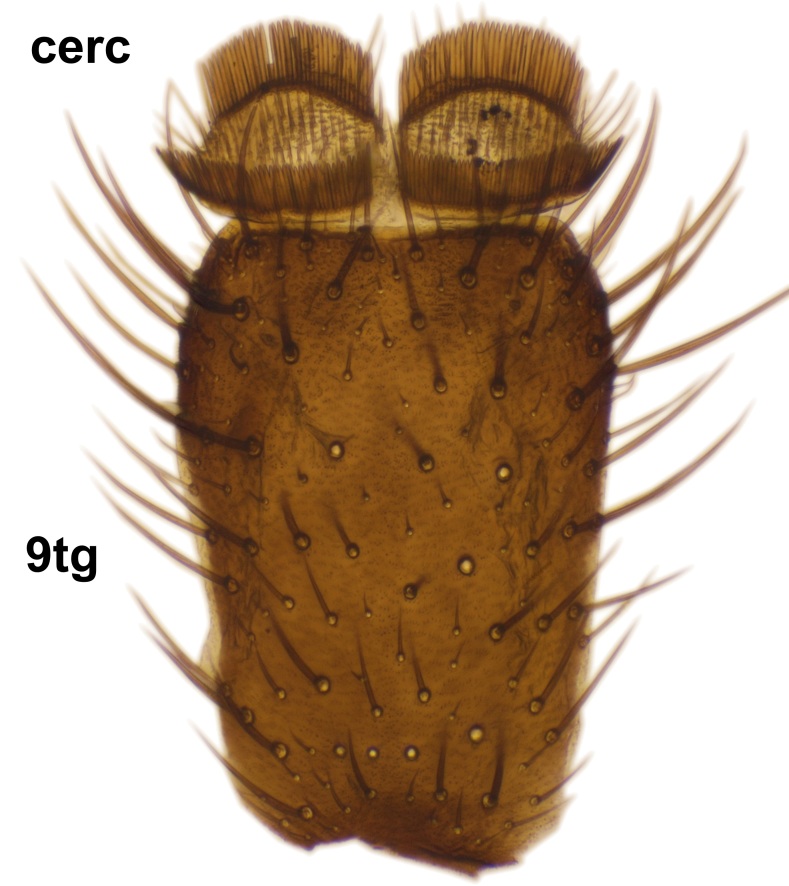
Cerci (cerc) and 9th tergite (9tg), dorsal view.

**Figure 7c.**
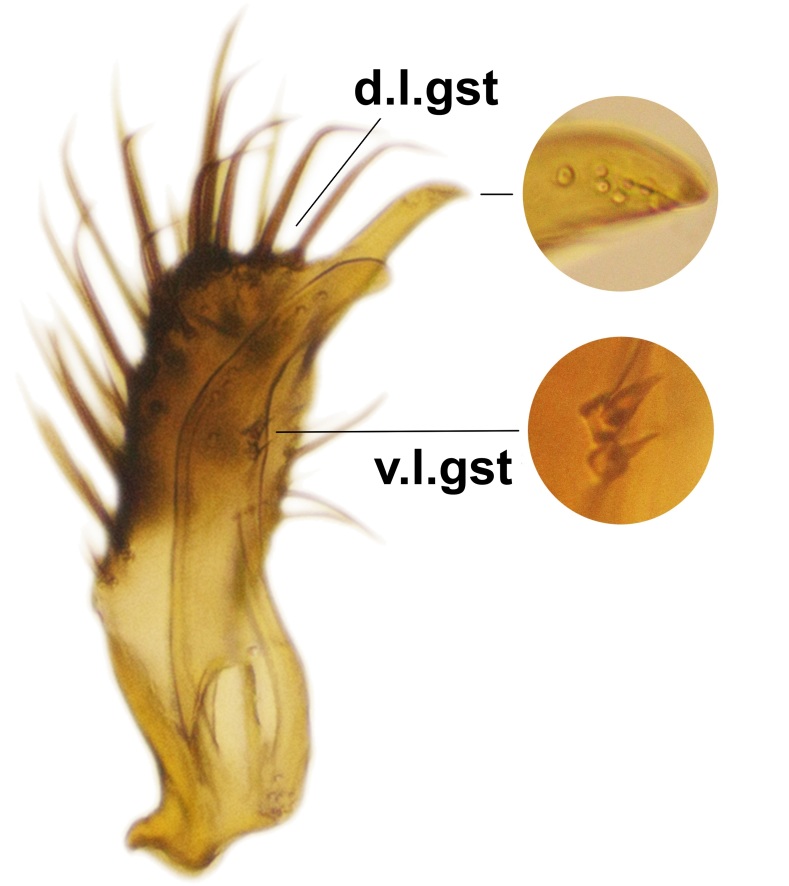
Gonostylus, lateral view. d.l.gst=dorsal lobe of gst, v.l.gst=ventral lobe of gst. Upper insert shows apex of d.l.gst and lower one two spines on the v.l.gst.

**Figure 7d.**
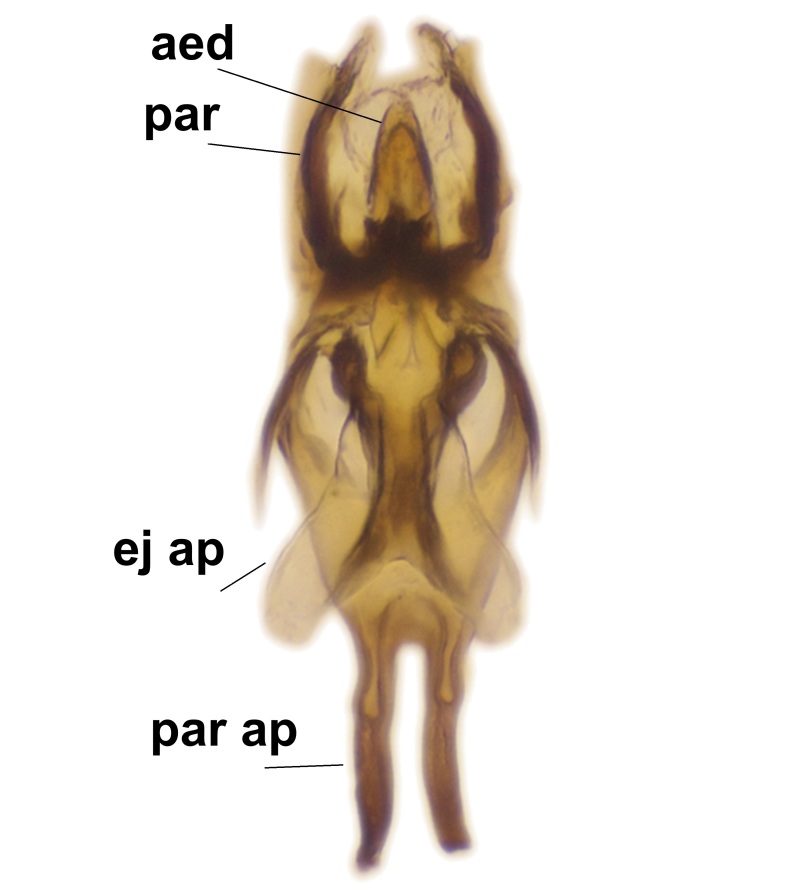
Aedeagal complex, dorsal view. aed=aedeagus, par=paramere, ej ap=ejaculatory apodeme, par ap=parameral apodeme.

**Figure 7e.**
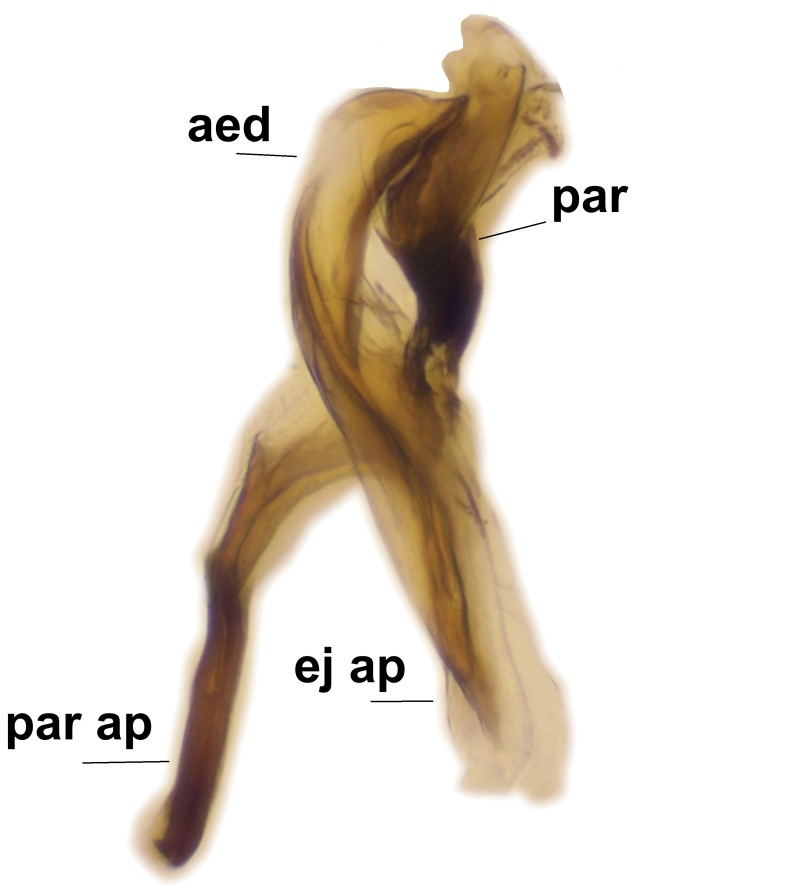
Aedeagal complex, lateral view.

**Figure 8a.**
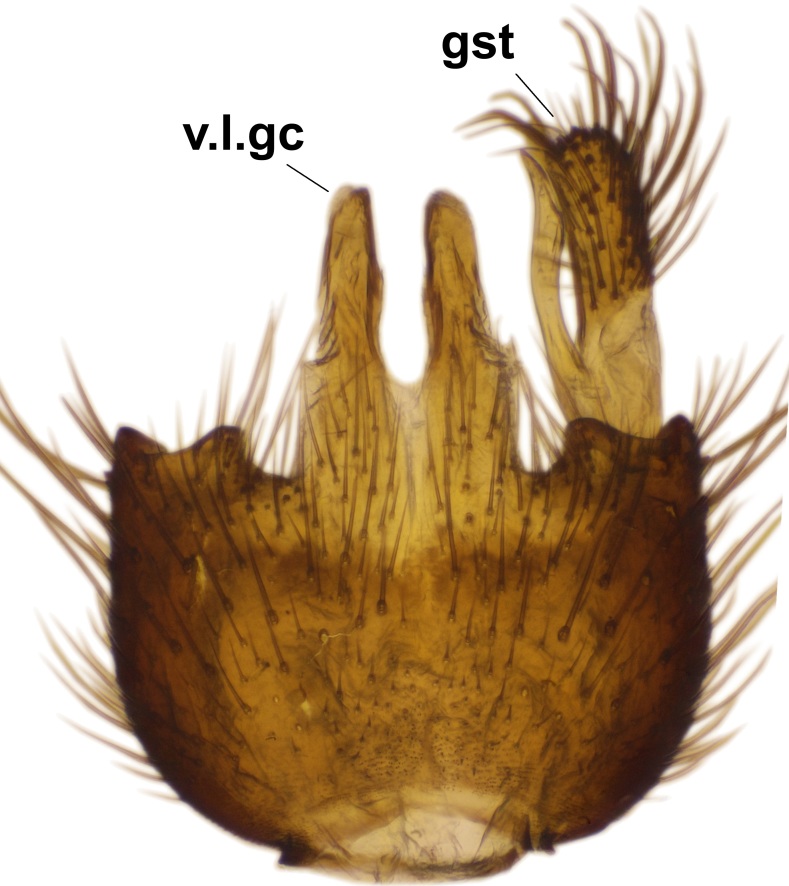
Hypopygium, ventral view. v.l.gc=ventral lobes of gonocoxites, gst=gonostylus.

**Figure 8b.**
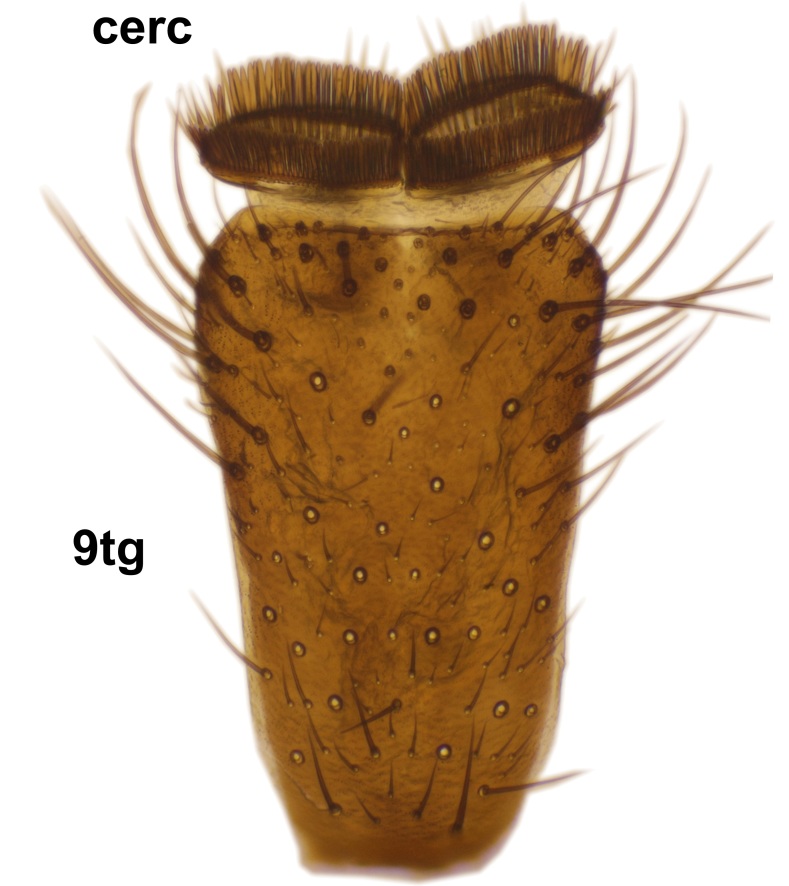
Cerci (cerc) and 9th tergite (9tg), dorsal view.

**Figure 8c.**
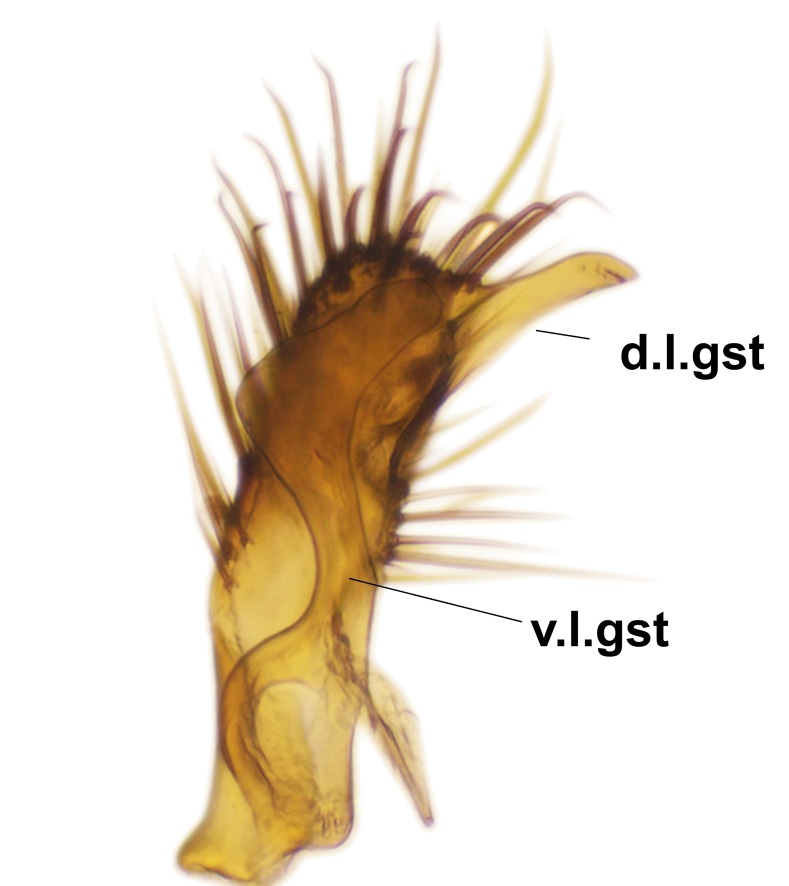
Gonostlys, lateral view. d.l.gst=dorsal lobe of gonostylus, v.l.gst=ventral lobe of gonostylus.

**Figure 8d.**
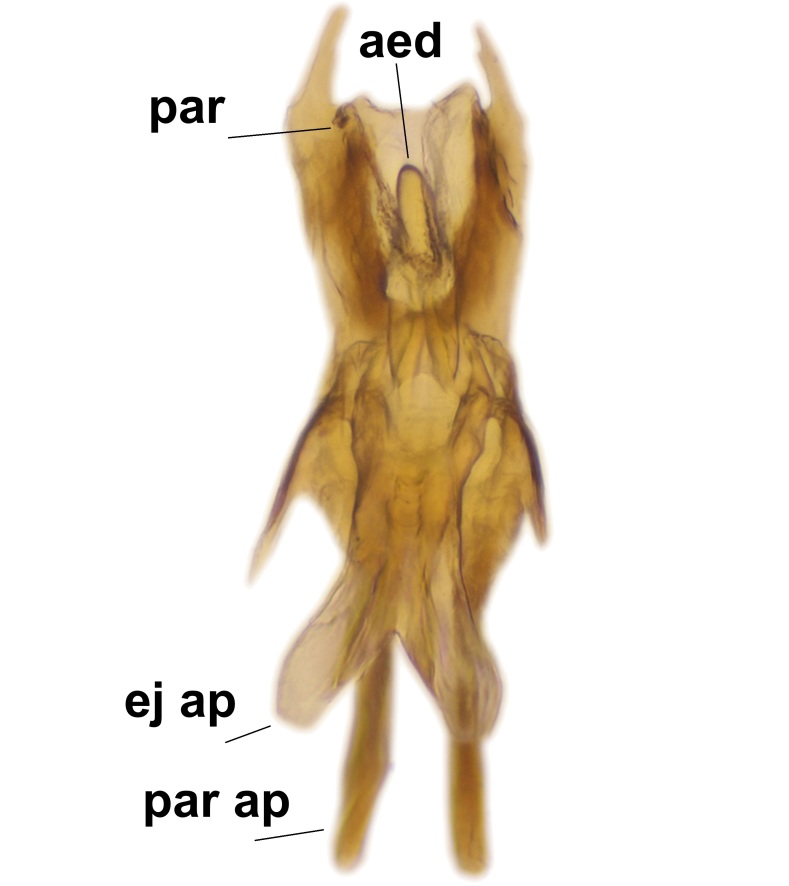
Aedeagal complex, dorsal view. par ap=parameral apodemes, ej ap=ejaculatory apodemes, par=parameres, aed=aedeagus.

**Figure 8e.**
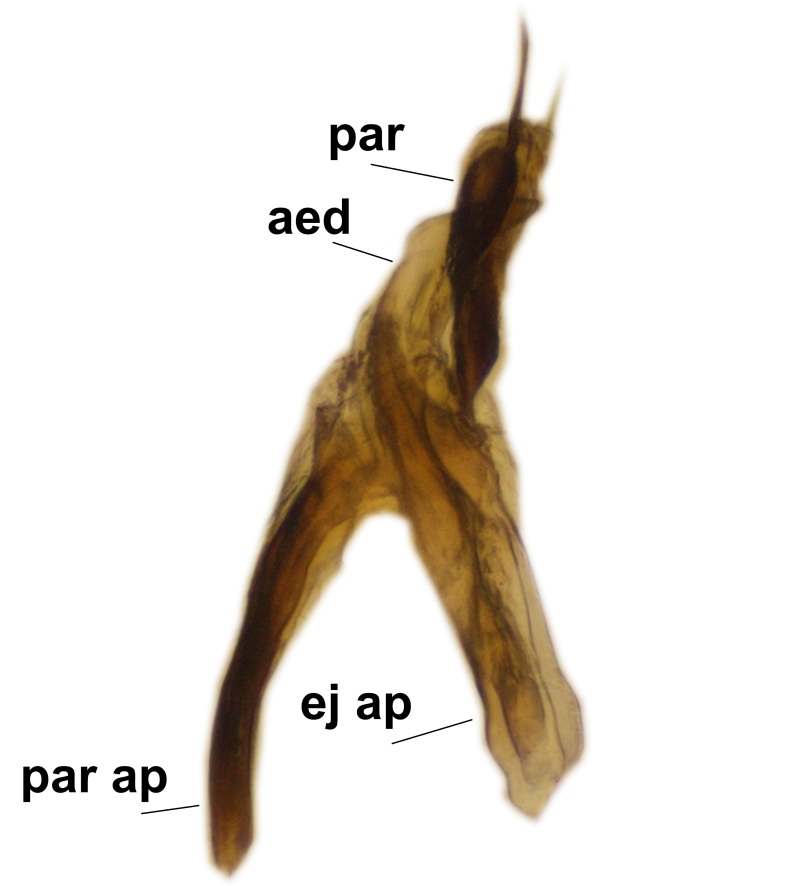
Aedeagal complex, lateral view.

**Figure 9a.**
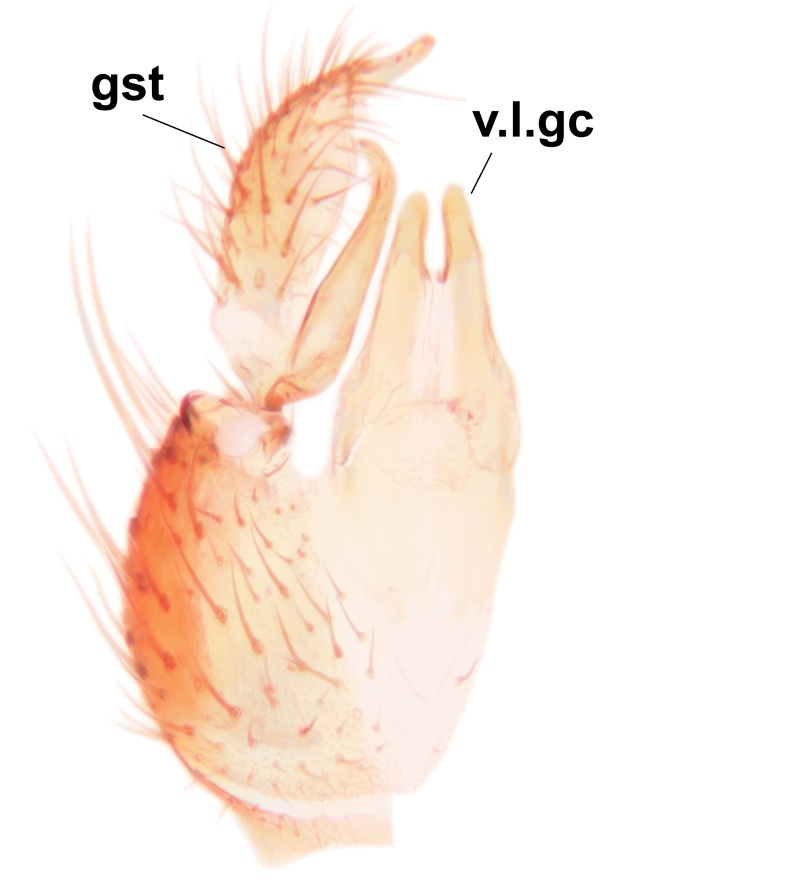
Hypopygium, ventral view. vl gc=ventral lobe of gonocoxites, gst=gonostylus.

**Figure 9b.**
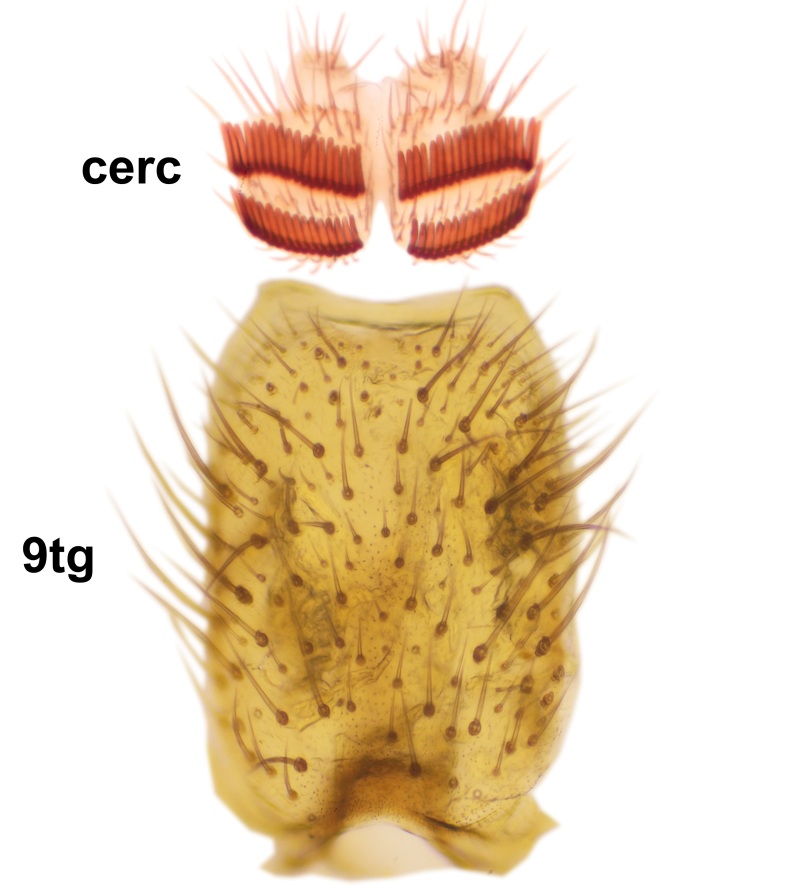
Cerci (cerc) and 9th tergite (9tg), dorsal view.

**Figure 9c.**
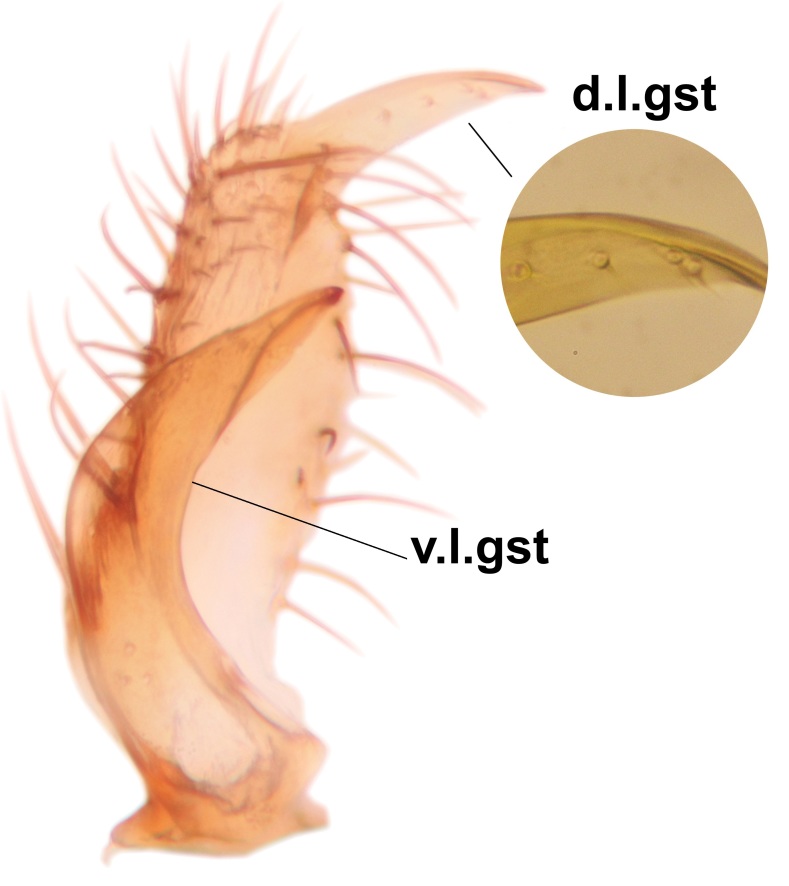
Gonostylus, lateral view. d.l.gst=dorsal lobe of gonostylus, v.l.gst=ventral lobe of gonostylus.

**Figure 9d.**
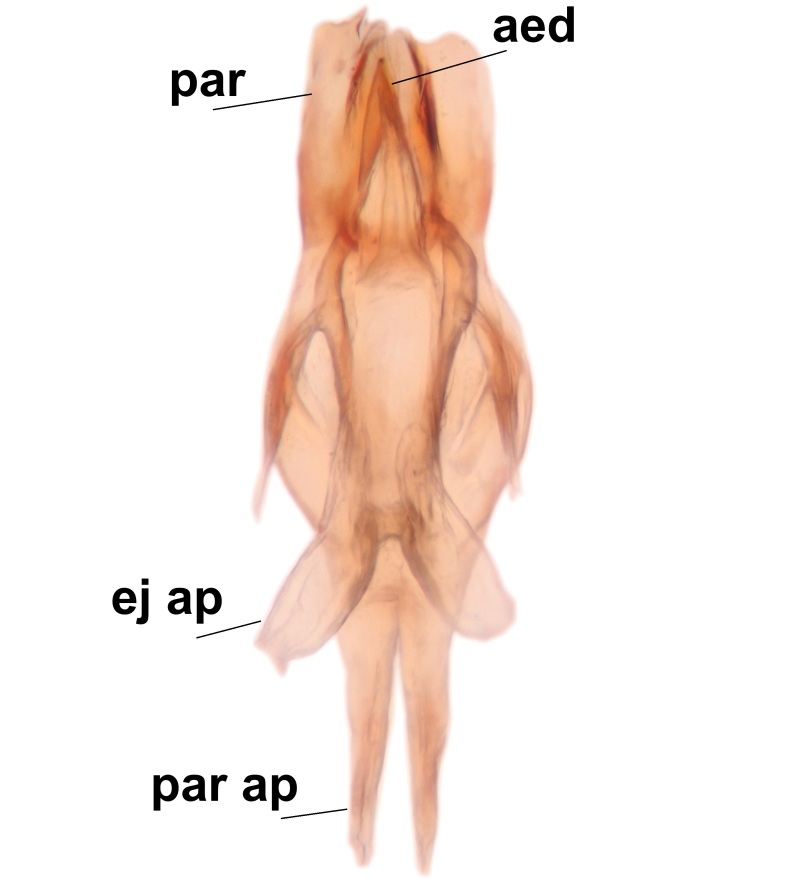
Aedeagal complex, dorsal view. aed=aedeagus, par=parameres, par ap=parameral apodemes, ej ap=ejaculatory apodemes.

**Figure 9e.**
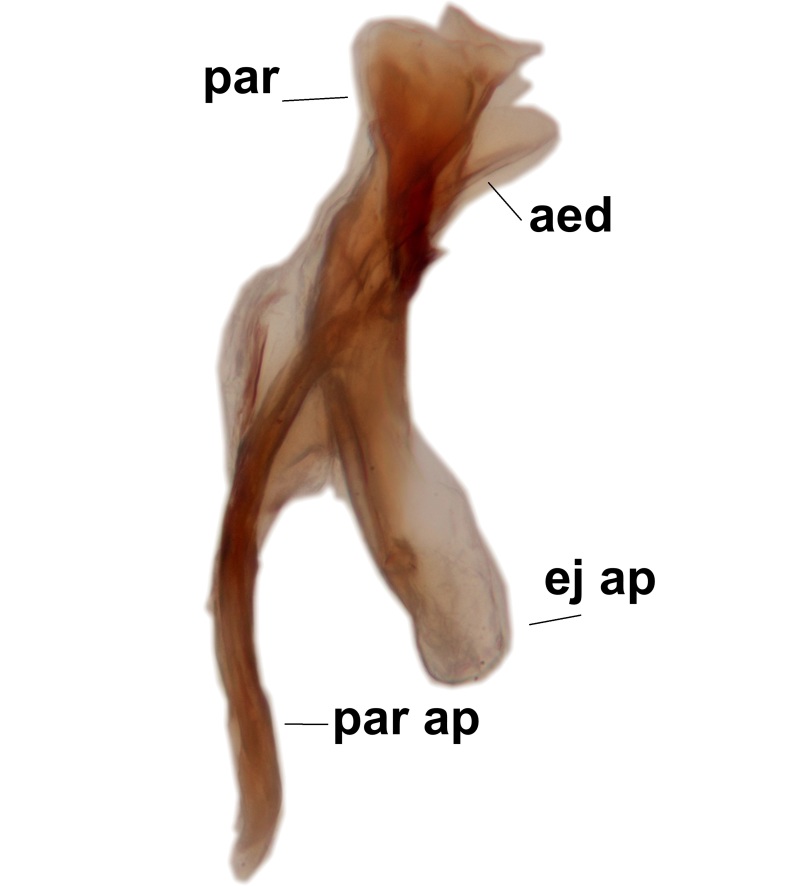
Aedeagal complex, lateral view.

**Figure 10a.**
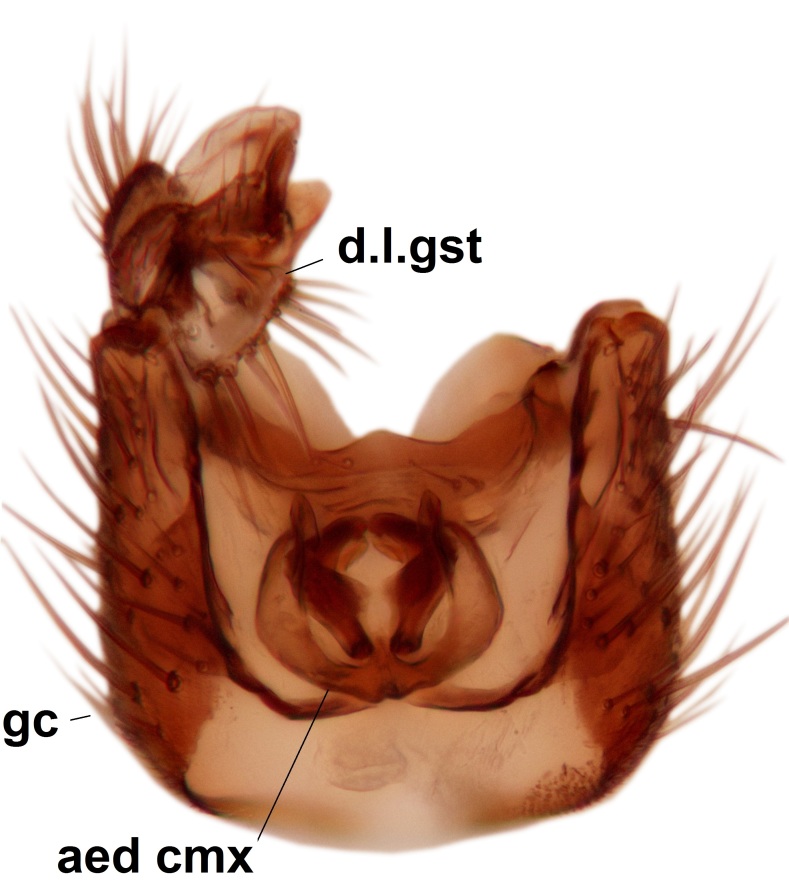
Hypopygium, dorsal view. gst=gonostylus, d.l.gst=dorsal lobe of gst, gc=gonocoxites, aed cmx=aedeagal complex.

**Figure 10b.**
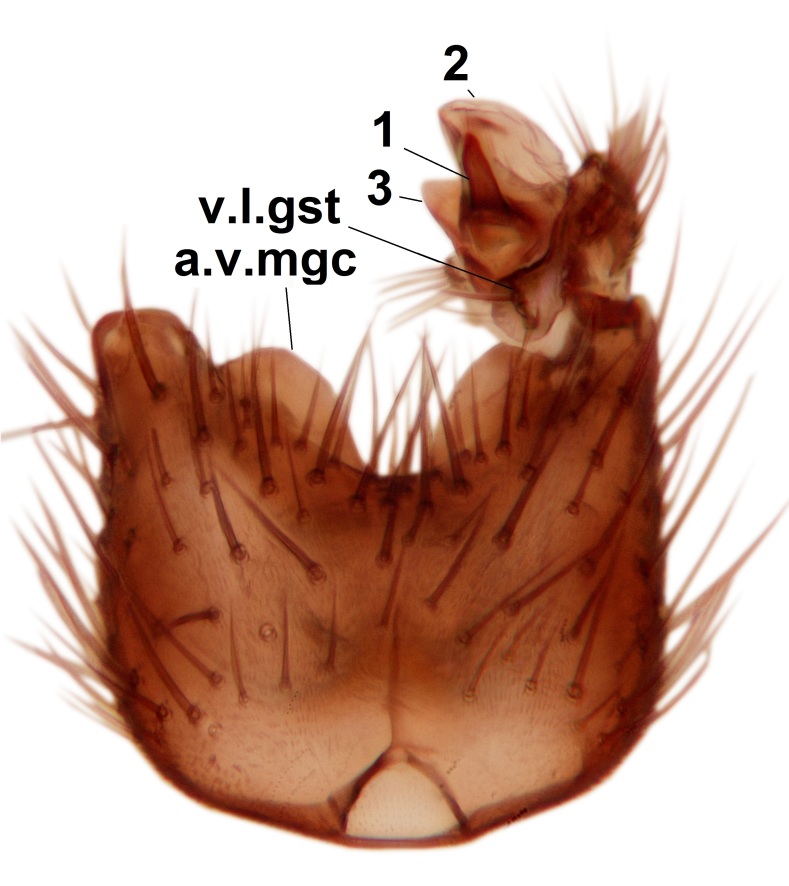
Hypopygium, ventral view. v.l.gst=ventral lobe of gonostylus, a.v.m.gc=ventroapical margin of gonocoxites, 1–3=projections of the mesial portion of gonostylus (see text).

**Figure 10c.**
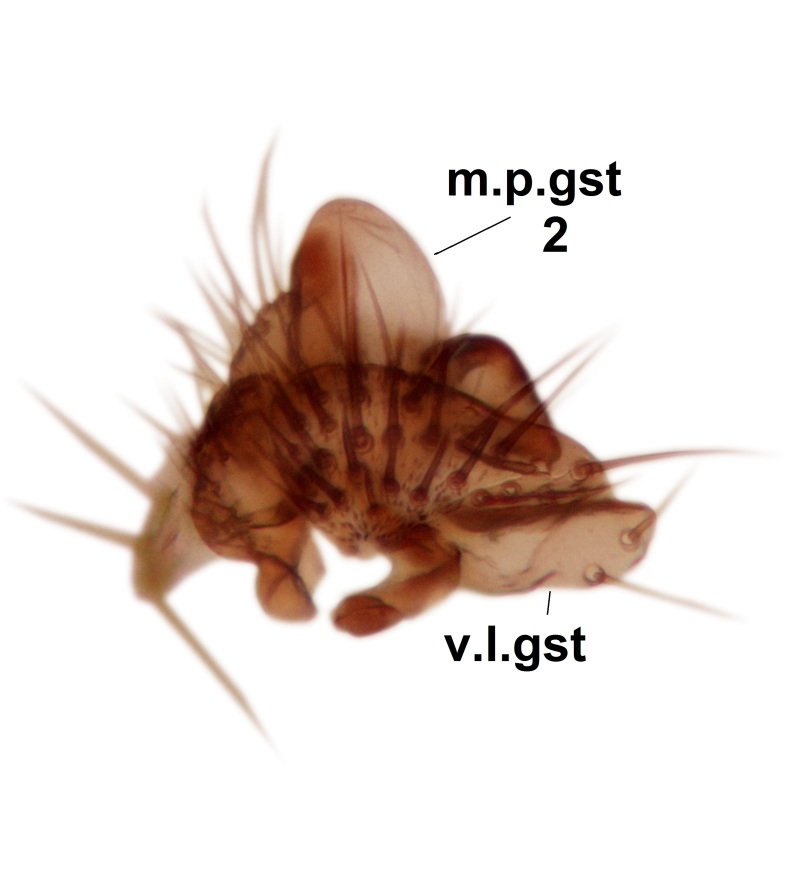
Gonostylus, outer lateral view. m.p.gst=mesial portion of gonostylus.

**Figure 10d.**
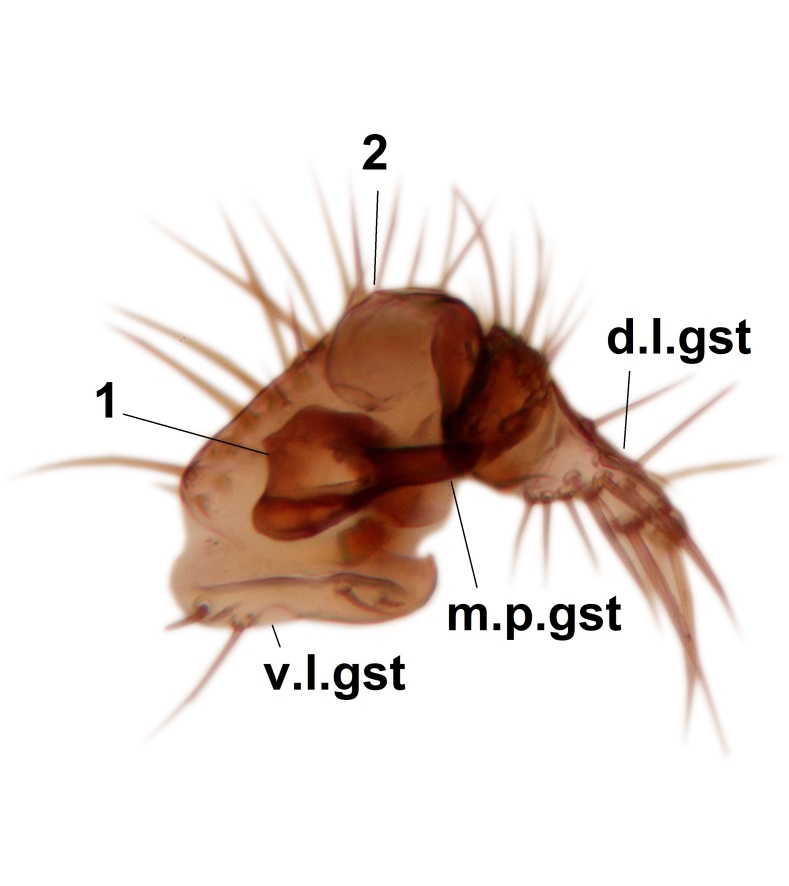
Gonostylus, inner lateral view.

**Figure 10e.**
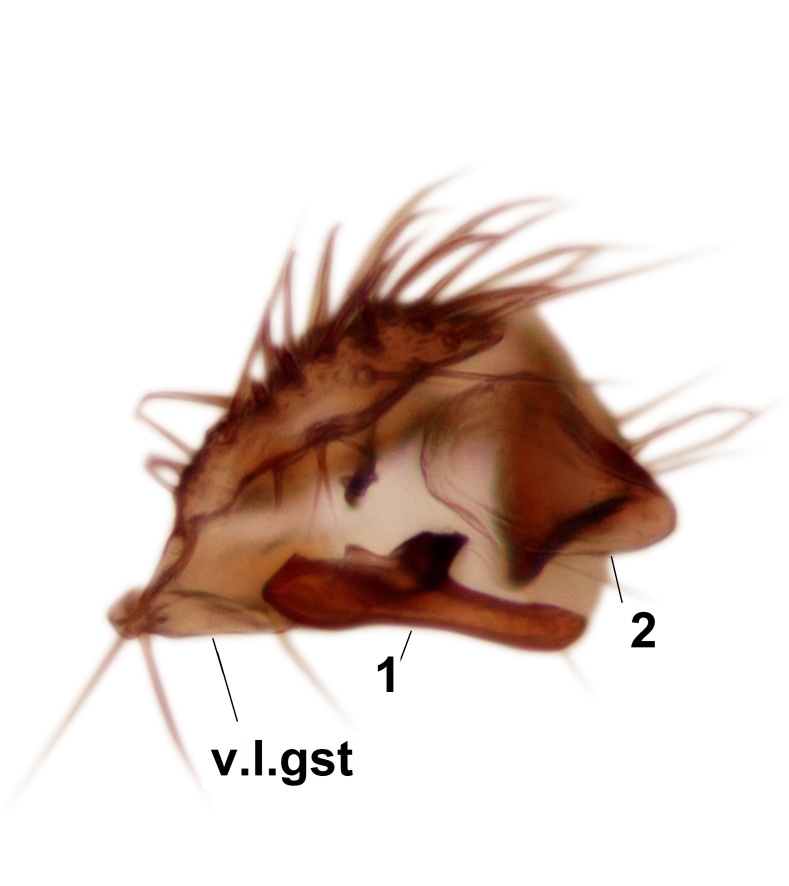
Gonostylus, dorsal view.

**Figure 10f.**
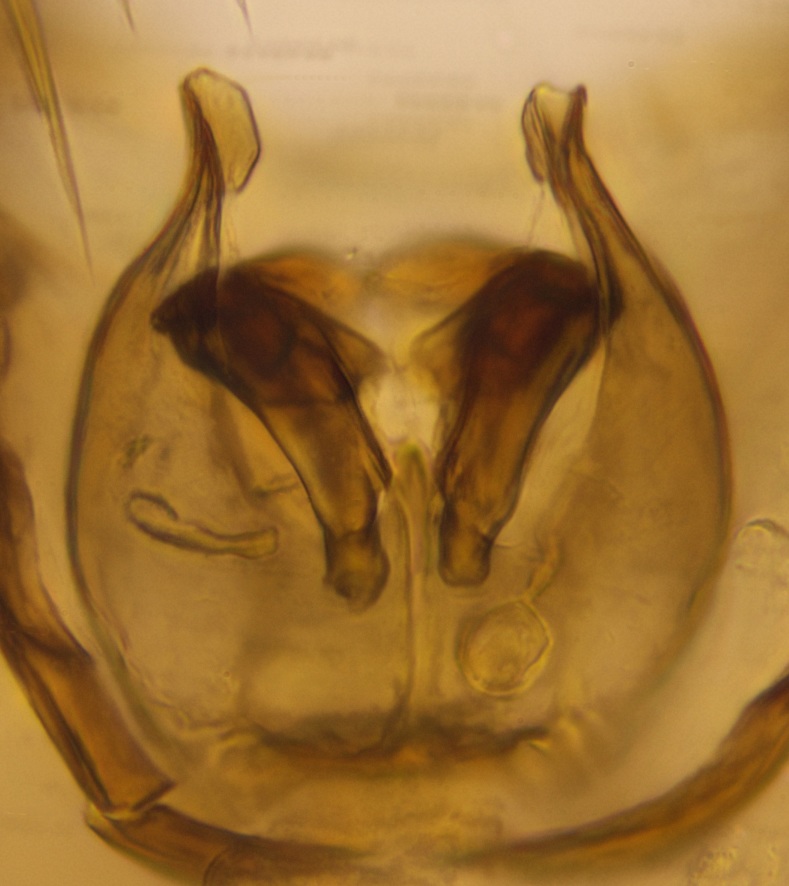
Aedeagal complex, dorsal view.

**Figure 11a.**
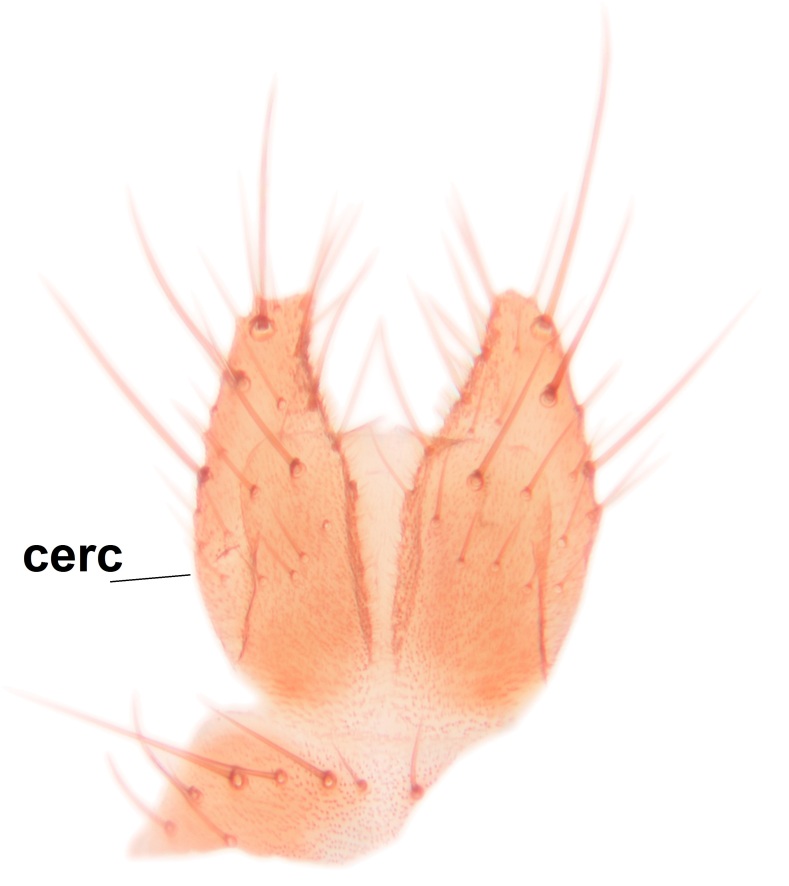
Cerci (cerc), dorsal view.

**Figure 11b.**
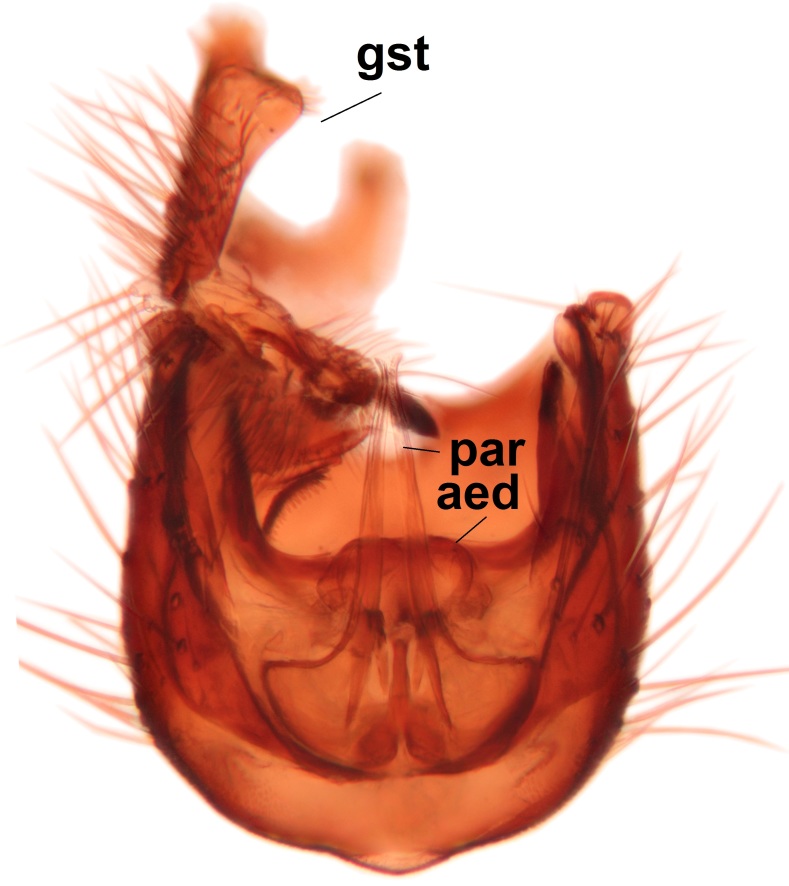
Hypopygium, dorsal view. gst=gonostylus, par=parameres, aed=aedeagus.

**Figure 11c.**
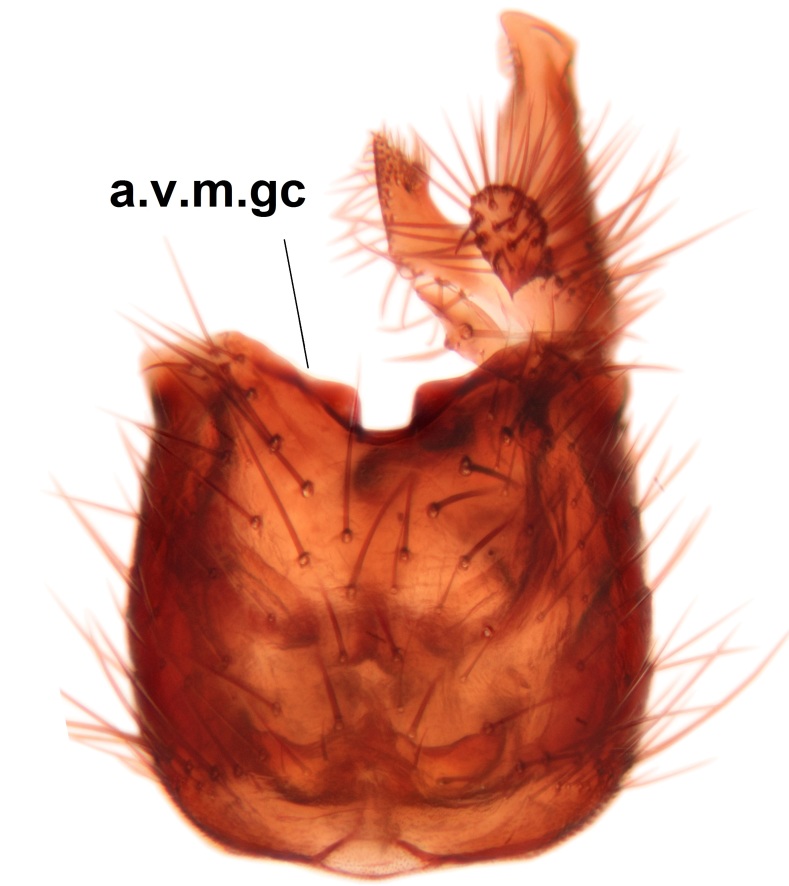
Hypopygium, ventral view. a.v.m.gc=ventroapical margin of gonocoxites.

**Figure 11d.**
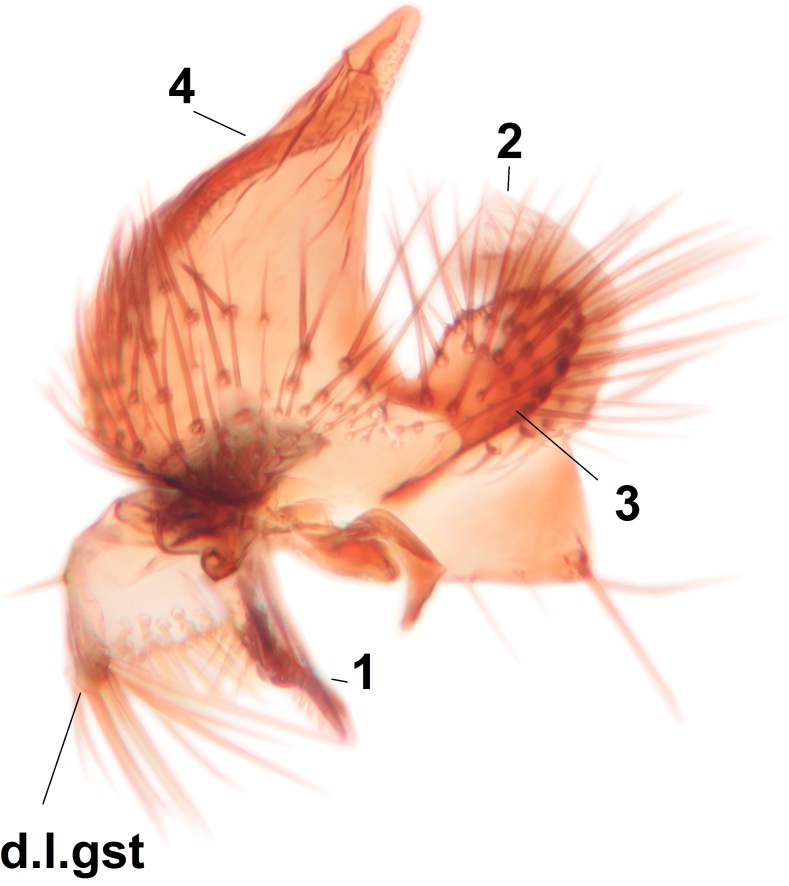
Gonostylus, outer lateral view. d.l.gst=dorsal lobe of gonostylus. 1-4, outgrowths of the gonostylus, see text.

**Figure 11e.**
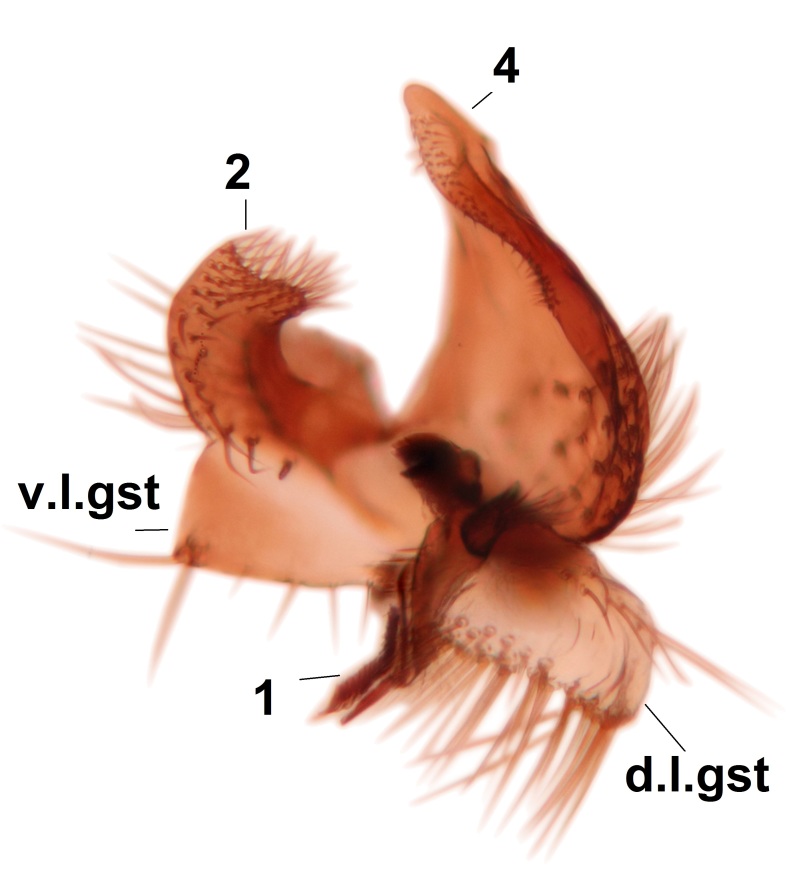
Gonostylus, inner lateral view. v.l.gst=ventral lobe of gonostylus.

**Figure 11f.**
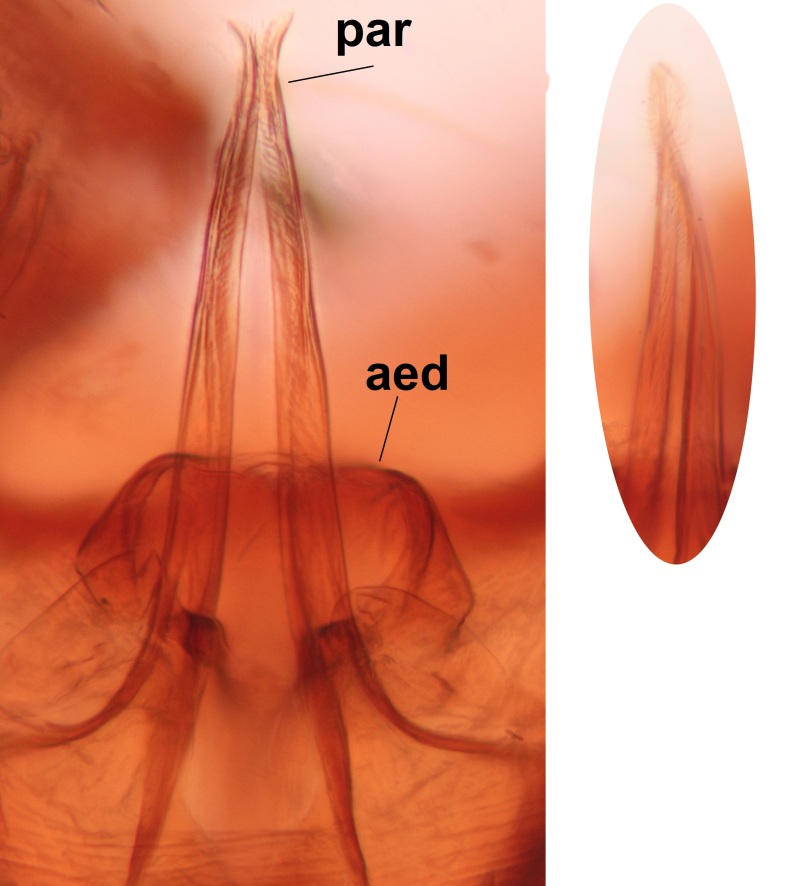
Aedeagus and parameres, dorsal view. Insert shows apices of parameres in lateral view.

**Figure 13a.**
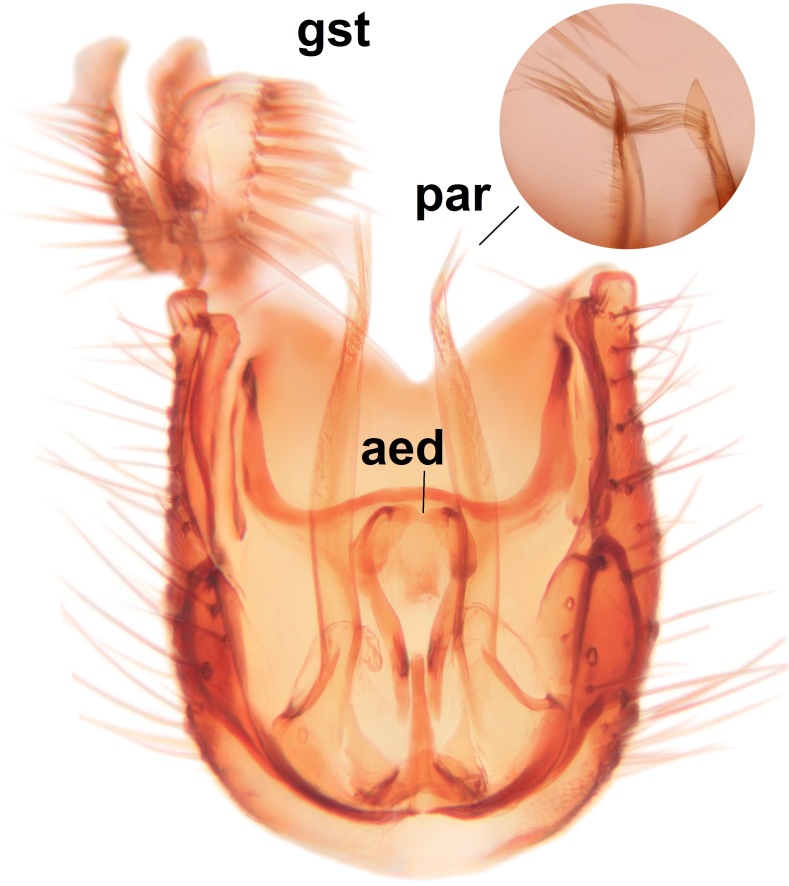
Hypopygium, dorsal view. Insert shows apices of parameres in lateral view.

**Figure 13b.**
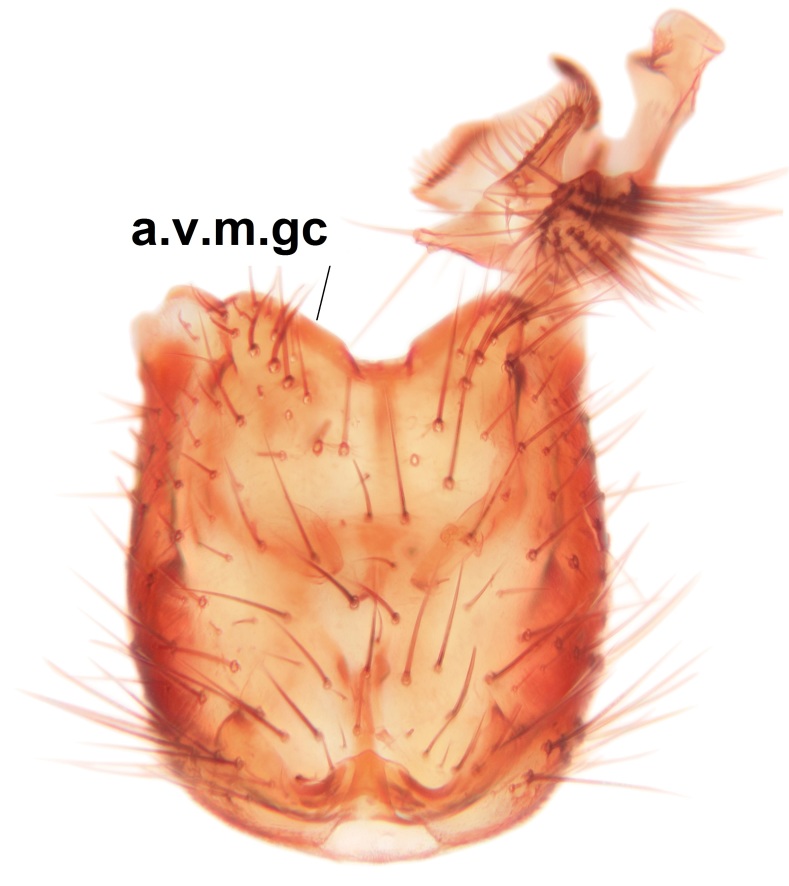
Hypopygium, ventral view.

**Figure 13c.**
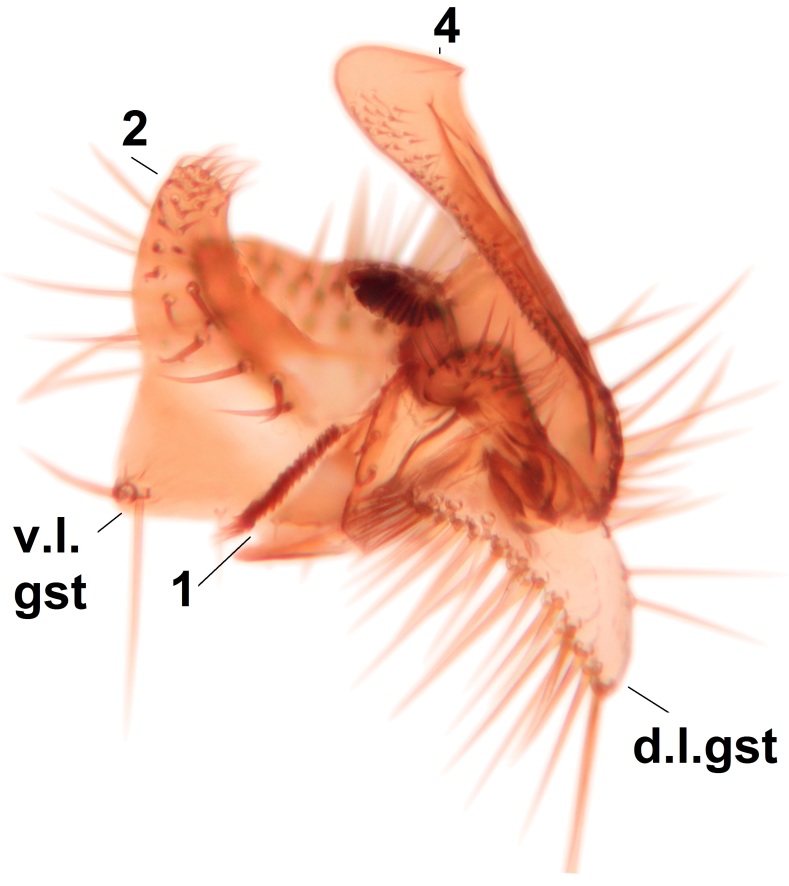
Gonostylus, inner lateral view.

**Figure 13d.**
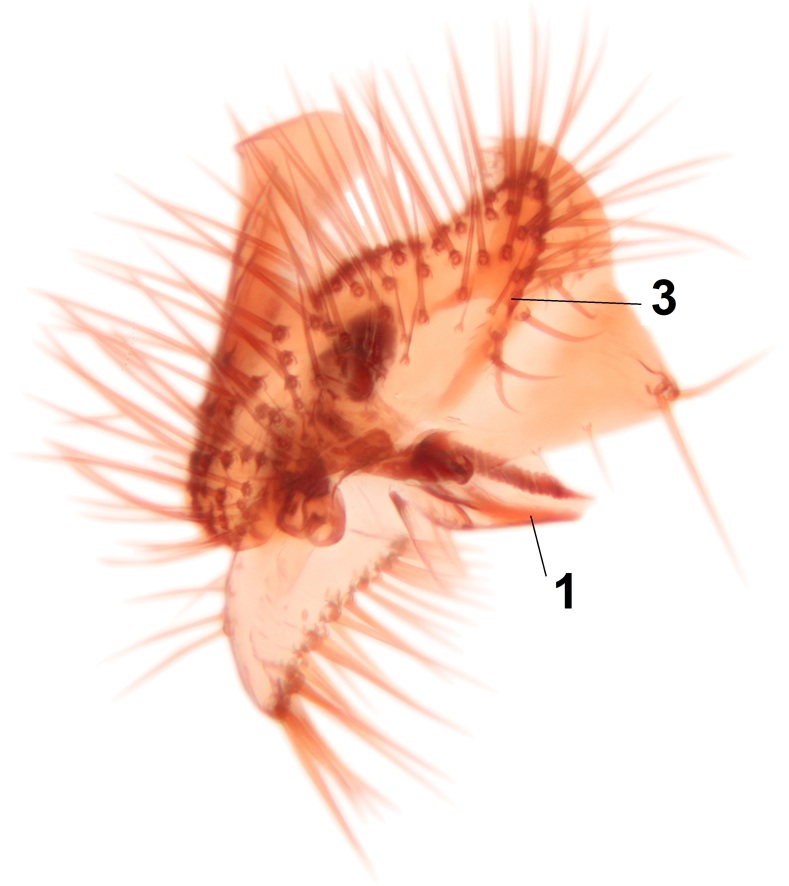
Gonostylus, outer lateral view.

**Figure 14a.**
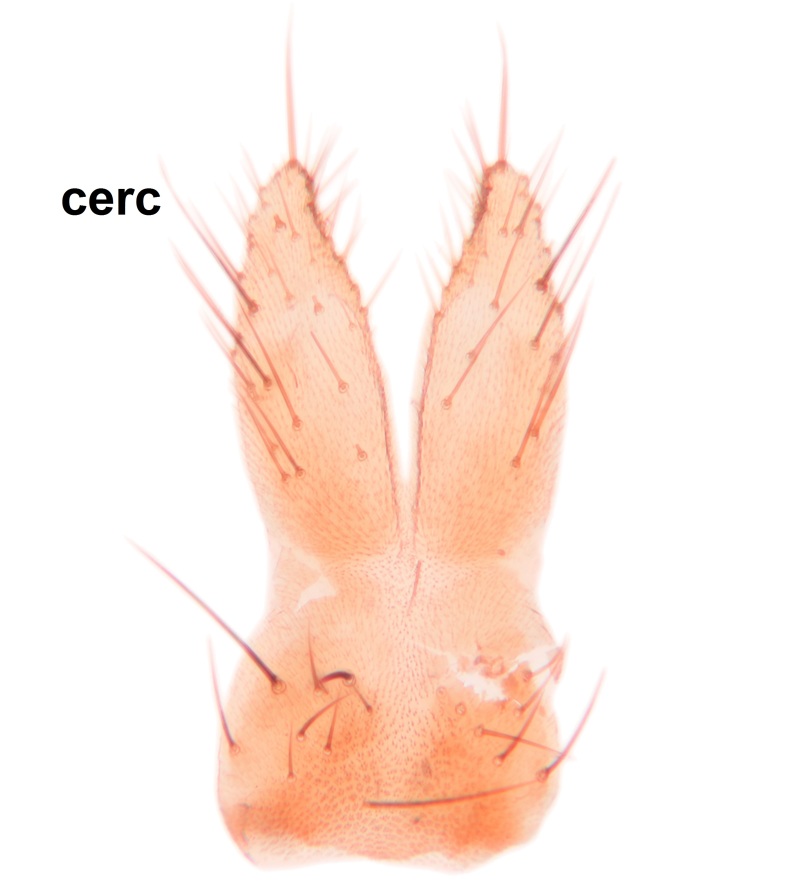
Cerci, dorsal view.

**Figure 14b.**
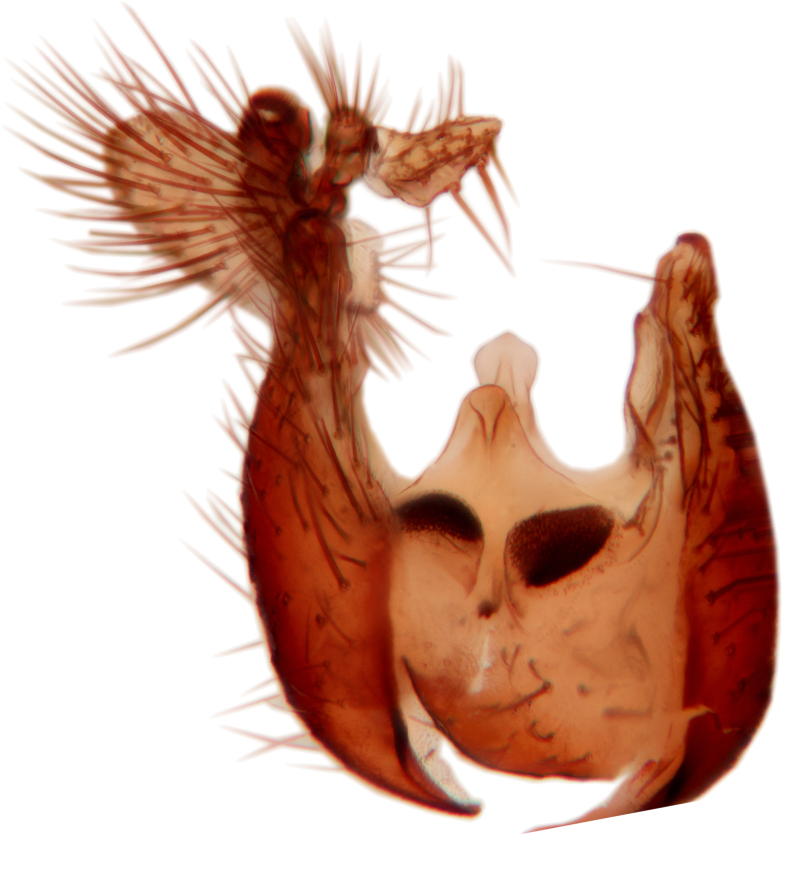
Hypopygium, dorsal view.

**Figure 14c.**
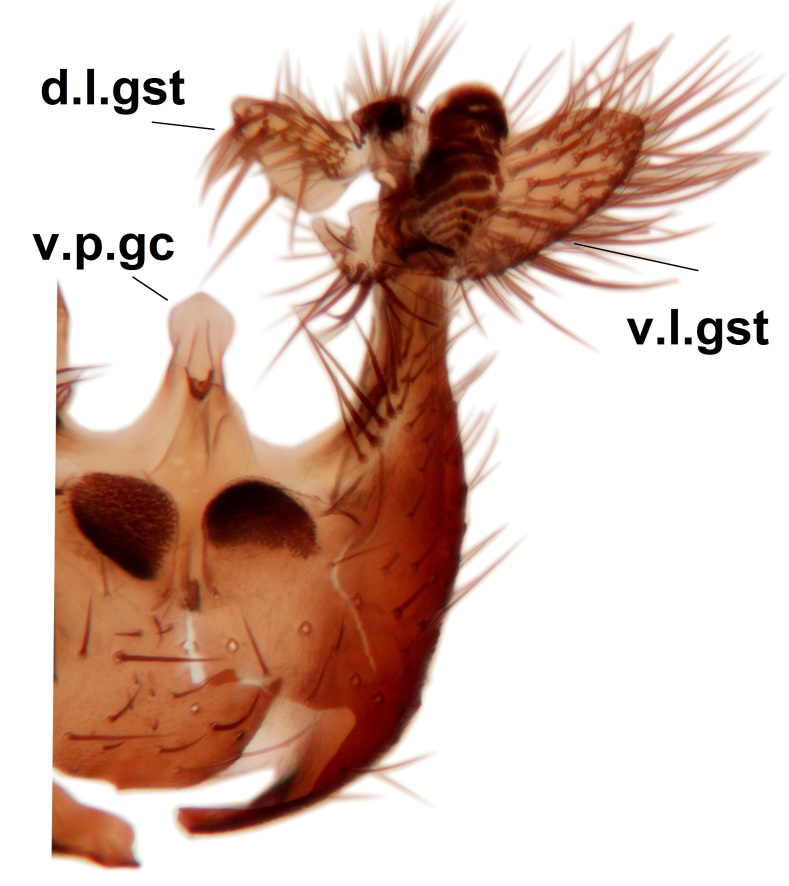
Hypopygium, ventral view. d.l.gst=dorsal lobe of gonostylus, v.l.gst=ventral lobe of gonostylus, v.p.gc=ventrocaudal projection of gonocoxites.

**Figure 14d.**
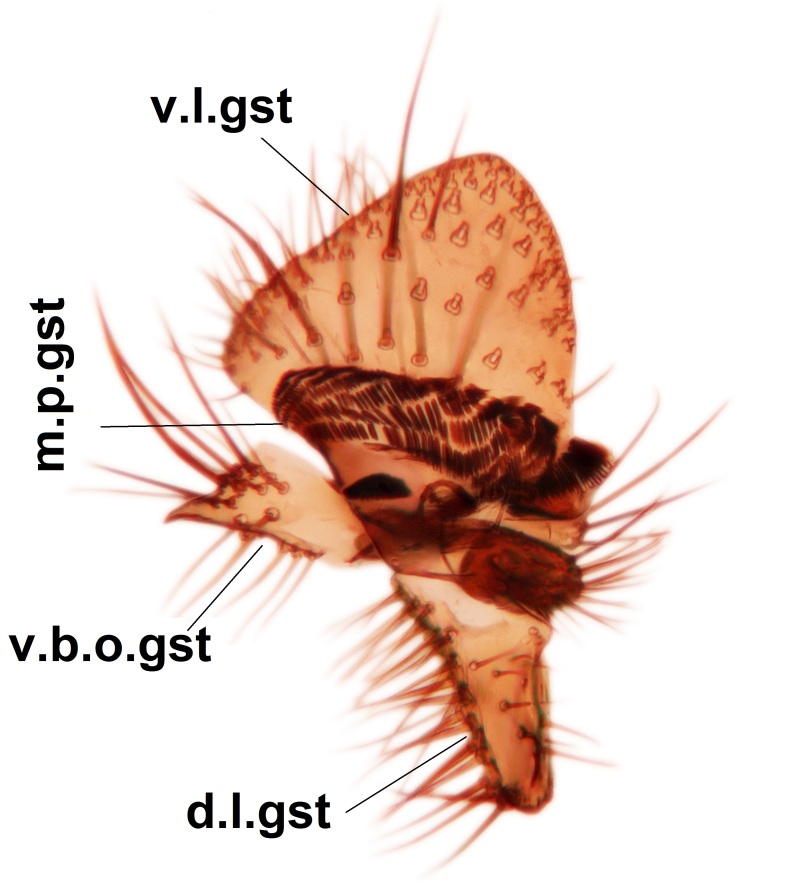
Gonostylus, inner lateral view. m.p.gst=mesial portion of gonostylus, v.b.o.gst=ventrobasal outgrowth of v.l.gst.

**Figure 14e.**
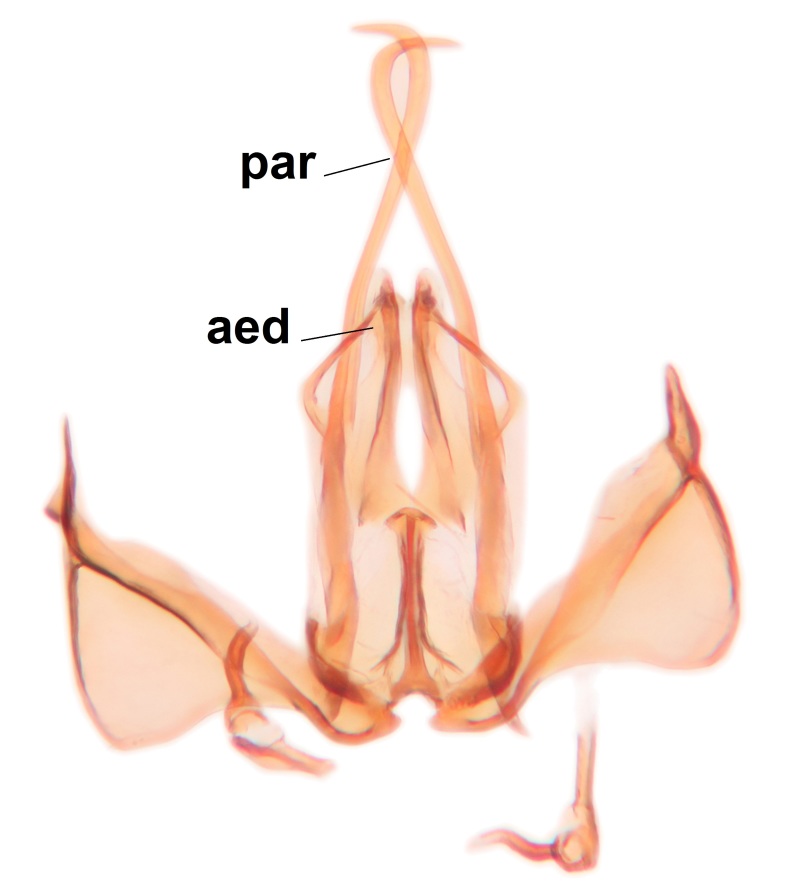
Aedeagal complex, dorsal view. par=parameres, aed=aedeagus.

**Figure 14f.**
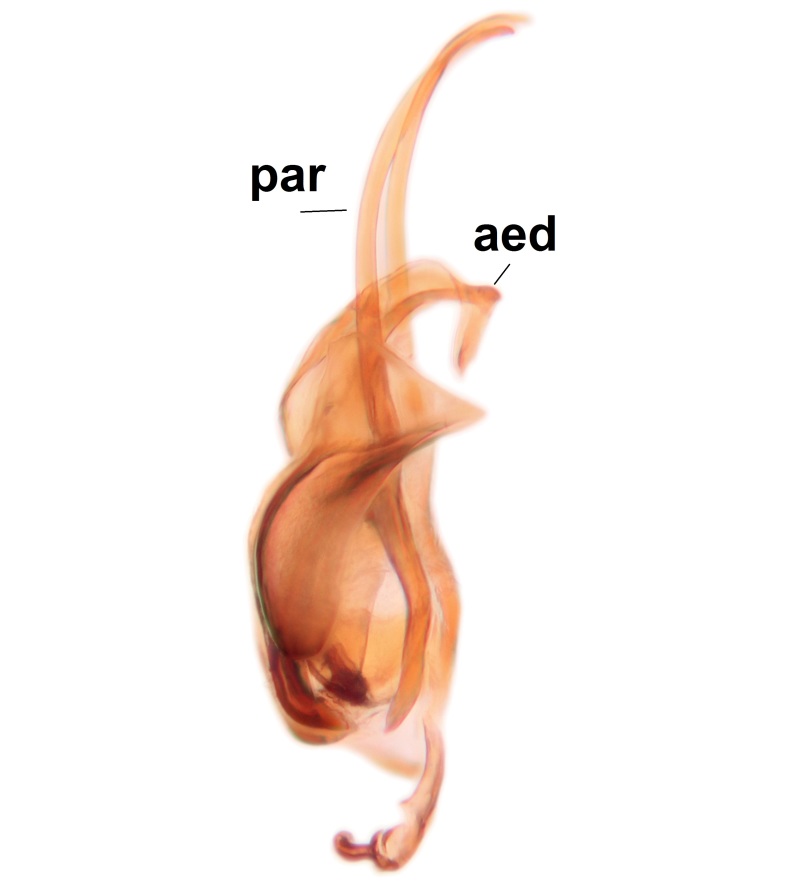
Aedeagal complex, lateral view.

**Figure 15a.**
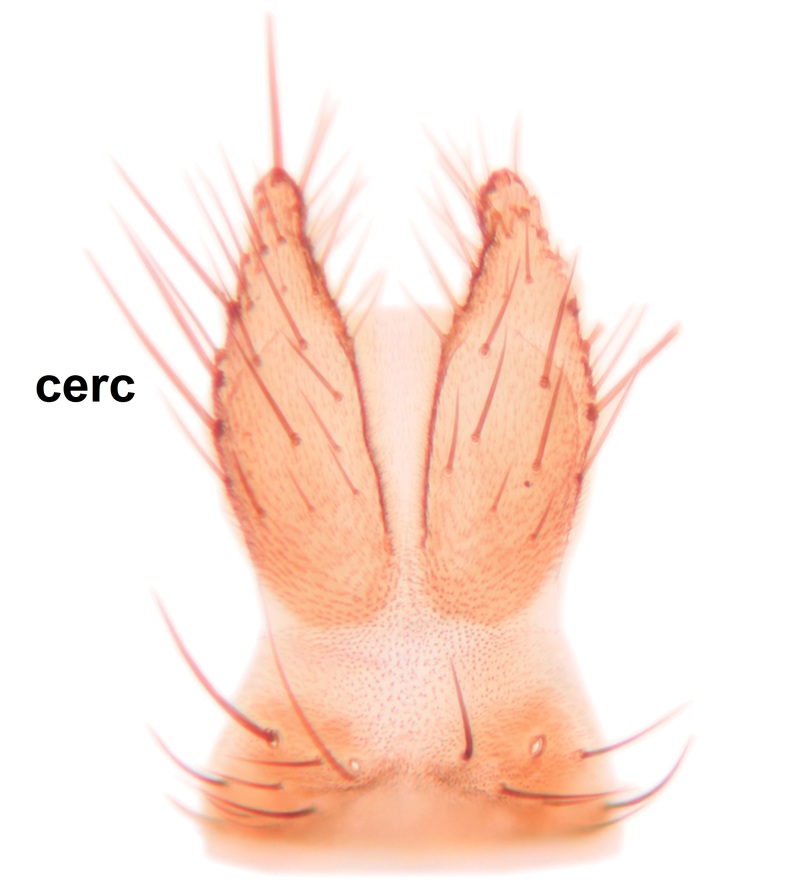
Cerci, dorsal view.

**Figure 15b.**
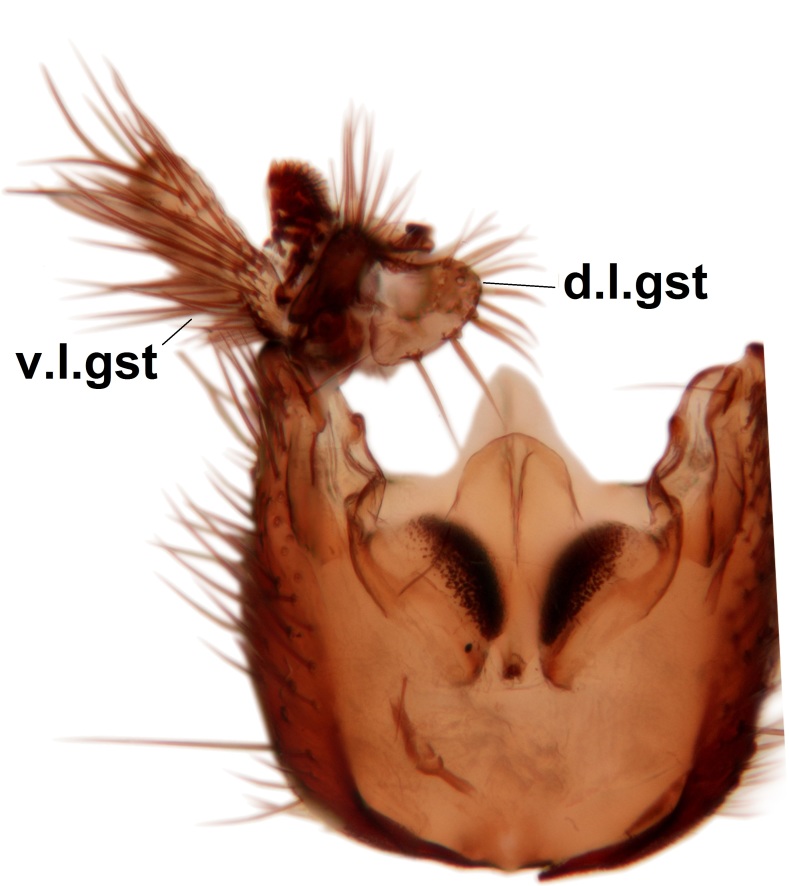
Hypopygium, dorsal view.

**Figure 15c.**
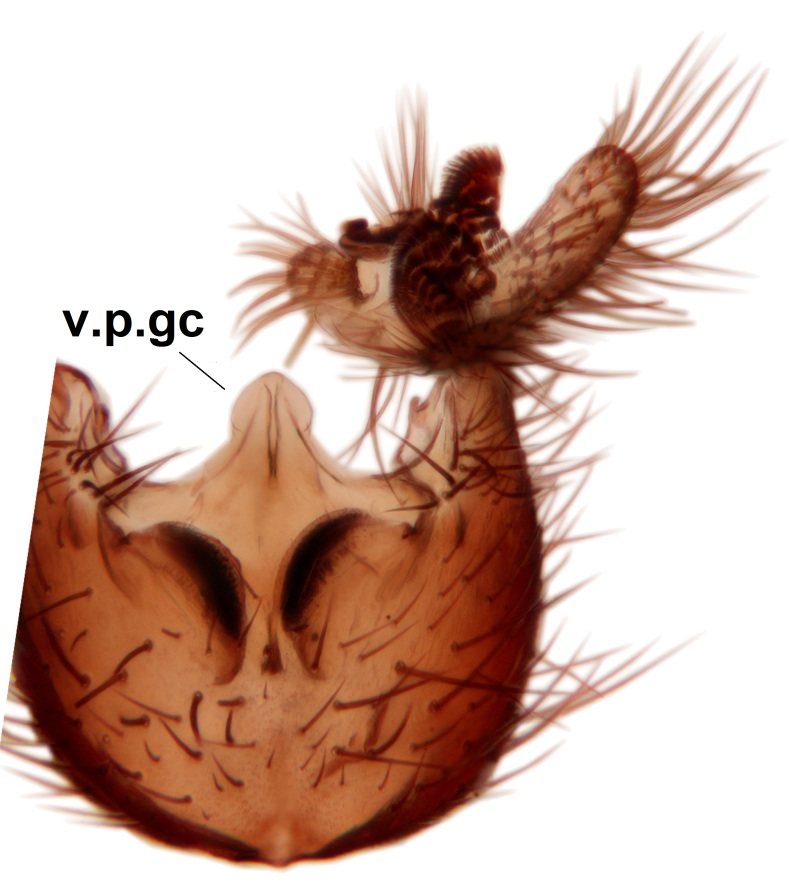
Hypopygium, ventral view.

**Figure 15d.**
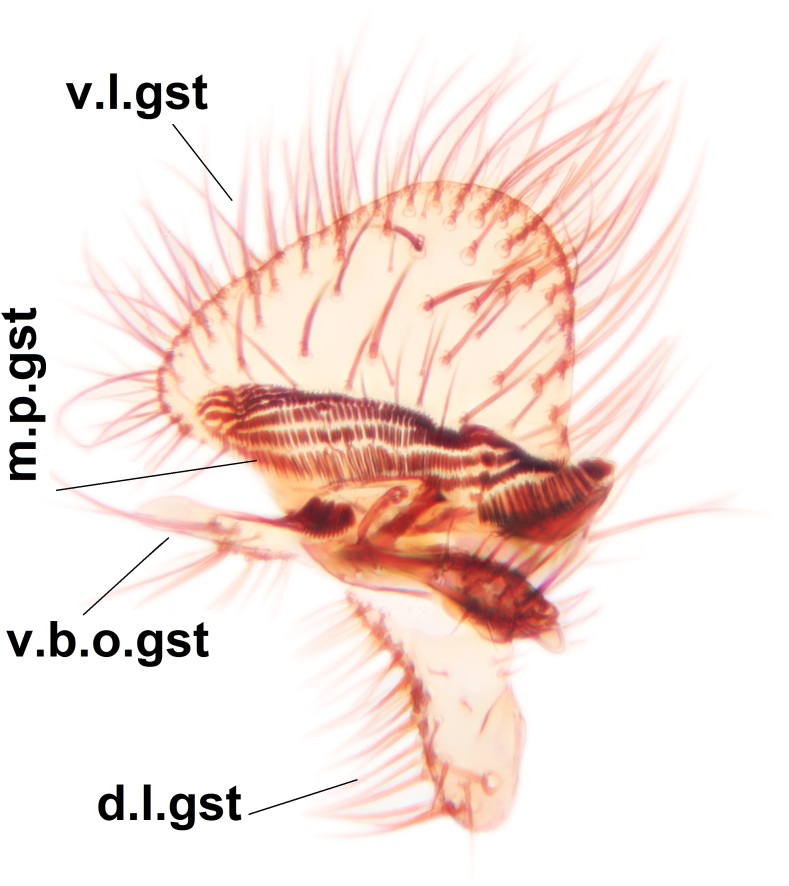
Gonostylus, inner lateral view.

**Figure 15e.**
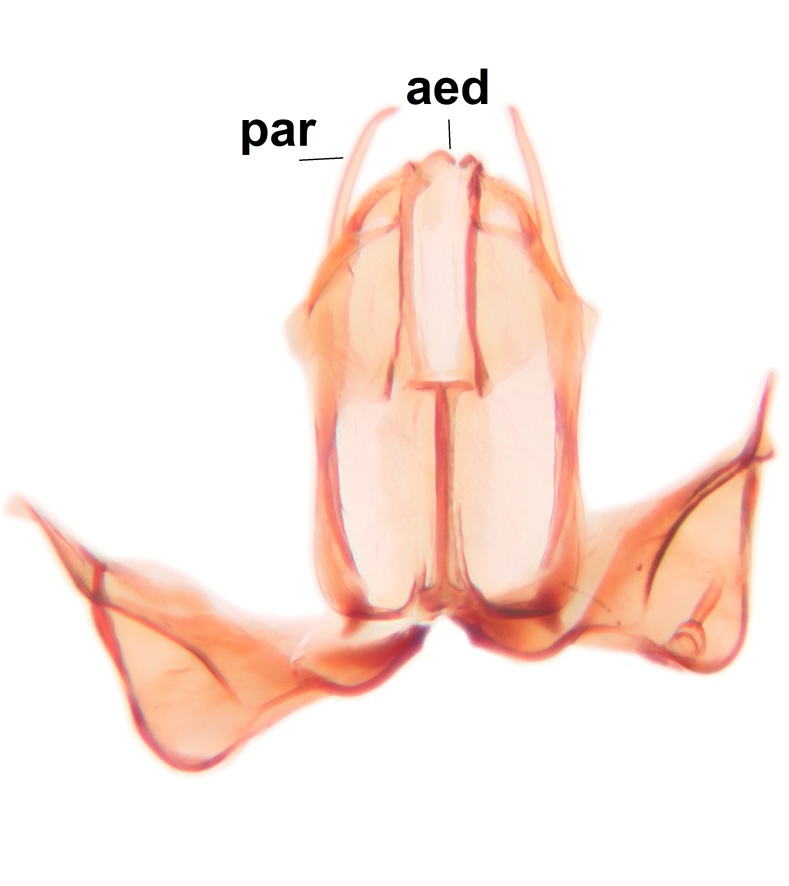
Aedeagal complex, dorsal view.

**Figure 15f.**
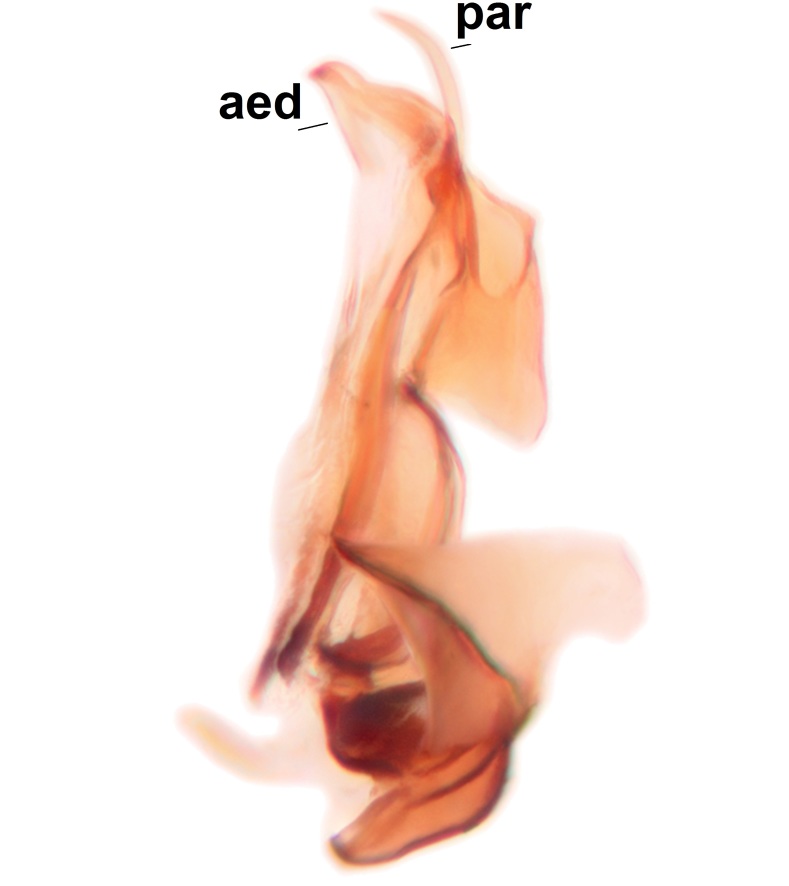
Aedeagal complex, lateral view.

**Figure 17a.**
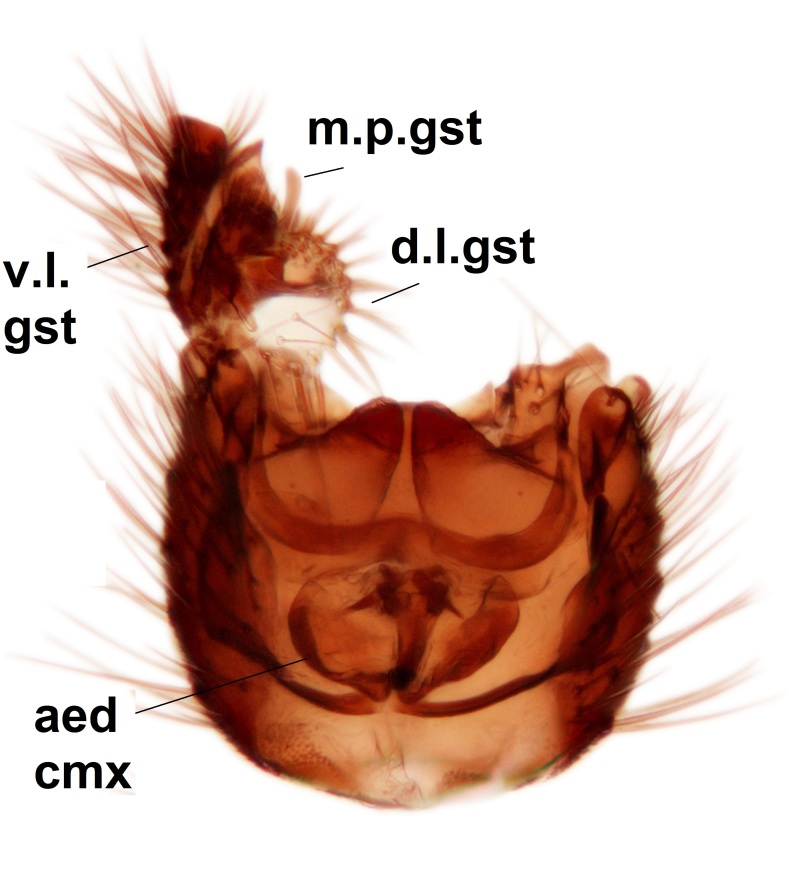
Hypopygium, dorsal view. d.l.gst=dorsal lobe of gonostylus, v.l.gst=ventral lobe of gonostylus, m.p.gst=mesial portion of gonostylus, aed cmx=aedeagal complex.

**Figure 17b.**
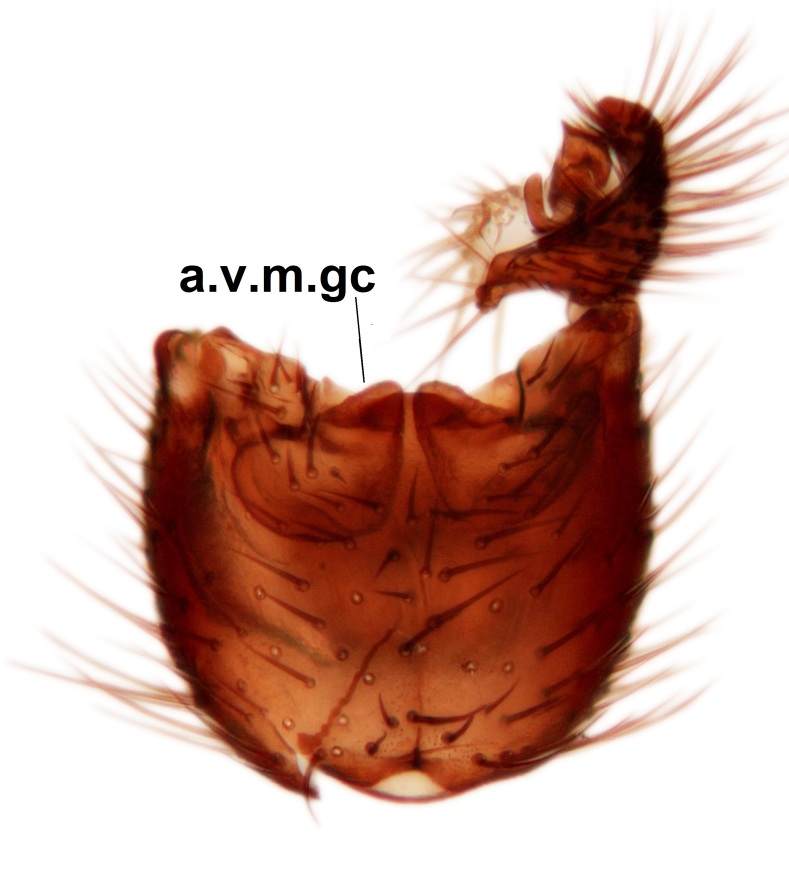
Hypopygium, ventral view. a.v.m.gc=ventroapical margin of gonocoxites.

**Figure 17c.**
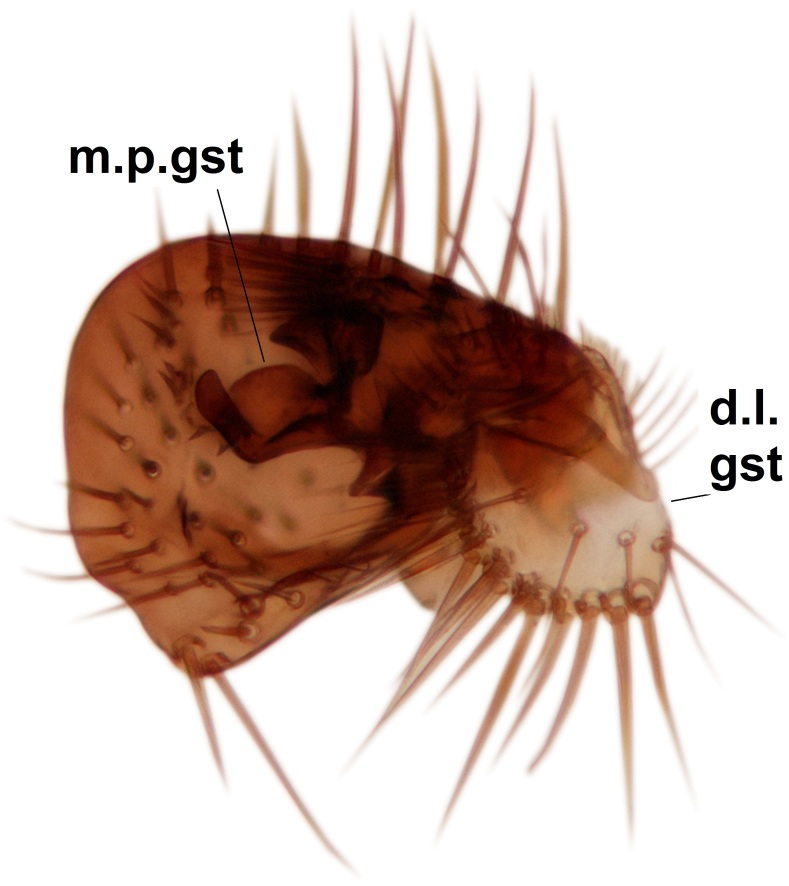
Gonostylus, inner lateral view.

**Figure 17d.**
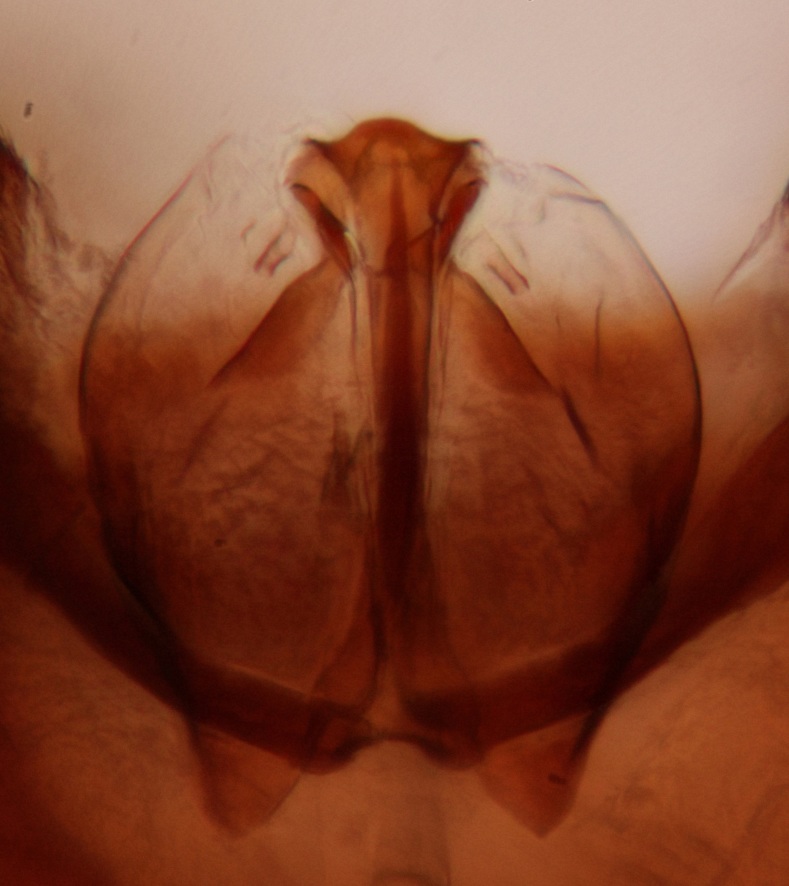
Aedeagal complex, dorsal view.

## References

[B3529762] Amorim DS, Yeates D. (2006). Pesky gnats: Ridding dipteran classification of the “Nematocera”. Studia dipterologica.

[B3530831] Amorim D. S., Rindal E. (2007). Phylogeny of the Mycetophiliformia, with proposal of the subfamilies Heterotrichinae, Ohakuneinae, and Chiletrichinae for the Rangomaramidae (Diptera, Bibionomorpha). Zootaxa.

[B3530335] Borkent C. J., Wheeler T. A. (2012). Systematics and Phylogeny of Leptomorphus Curtis (Diptera: Mycetophilidae). Zootaxa.

[B3530797] Chandler P. J. (1992). Areview of the British Phronia Winnertz and Trichonta Winnertz (Dipt., Mycetophilidae). Entomologist’s Monthly Magazine.

[B3530860] deWaard Jeremy R., Ivanova Natalia V., Hajibabaei Mehrdad, Hebert Paul D. N. (2008). Assembling DNA Barcodes. Methods in Molecular Biology.

[B3530748] Dziedzicki H. (1889). Revue des espèces européennes du genre Phronia Winnertz avec la description des deux genres nouveaux: Macrobrachius et Megophthalmidia. Horae Soc. Ent. Ross..

[B3531591] Evenhuis N. (1997). Literatura Taxonomica Dipterorum (1758–1930).

[B3530495] Evenhuis N. (2006). Catalog of the Keroplatidae of the World (Insecta: Diptera). Bishop Museum Bulletin in Entomology.

[B3530656] Gagné R. J. (1975). A revision of the Nearctic species of the genus Phronia (Diptera: Mycetophiilidae). Transactions of the American Entomological Society.

[B3531344] Geiger Matthias, Moriniere Jerome, Hausmann Axel, Haszprunar Gerhard, Wägele Wolfgang, Hebert Paul, Rulik Björn (2016). Testing the Global Malaise Trap Program – How well does the current barcode reference library identify flying insects in Germany?. Biodiversity Data Journal.

[B3531377] Giglio-Tos E. (1890). Nuove specie di Ditteri del Muzeo Zoologico di Torino. Bollettino dei Musei di Zoologia ed Anatomia Comparata della Regia Universita di Torino.

[B3530758] Hackman W. (1970). New species of the genus Phronia Winnertz (Diptera, Mycetophilidae) from Eastern Fennoscandia and notes on the synonymies in this genus. Notulae entomologicae.

[B3531577] Hackman W., Laštovka P., Matile L., Väisänen R., Soós A., Papp L. (1988). Family Mycetophilidae (Fungivoridae). Catalogue of Palaearctic Diptera. Volume 3. Ceratopogonidae - Mycetophilidae.

[B3530445] Hebert P. D. N., Cywinska A., Ball S. L., deWaard J. R. (2003). Biological identifications through DNA barcodes. Proceedings of the Royal Society B: Biological Sciences.

[B3530430] Hebert Paul D. N., Ratnasingham Sujeevan, Zakharov Evgeny V., Telfer Angela C., Levesque-Beaudin Valerie, Milton Megan A., Pedersen Stephanie, Jannetta Paul, deWaard Jeremy R. (2016). Counting animal species with DNA barcodes: Canadian insects. Philosophical Transactions of the Royal Society B: Biological Sciences.

[B3530529] Hutson A. M., Ackland D. M., Kidd L. (1980). Mycetophilidae (Bolitophilinae, Ditiomyiinae, Diadocidiinae, Keroplatinae, Sciophilinae and Manotinae). Diptera, Nematocera. Handbooks for Identification of British Insects.

[B3531397] Ihalainen E., Lindstedt C. (2012). Do avian predators select for seasonal polyphenism in the European map butterfly Araschnia
levana (Lepidoptera: Nymphalidae)?. Biological Journal of the Linnean Society.

[B3530646] Jakovlev J. (2011). Fungus gnats (Diptera: Sciaroidea) associated with dead wood and wood growing fungi: new rearing data from Finland and Russian Karelia and general analysis of known larval microhabitats in Europe. Entomologica Fennica.

[B3530666] Jakovlev J., Polevoi A. V. (2009). Two new species of the genus Phronia Winnertz (Diptera: Mycetophilidae) from Finland and Russian Karelia. Entomologica Fennica.

[B3529842] Jakovlev Jevgeni, Salmela Jukka, Polevoi Alexei, Penttinen Jouni, Vartija Noora-Annukka (2014). Recent noteworthy findings of fungus gnats from Finland and northwestern Russia (Diptera: Ditomyiidae, Keroplatidae, Bolitophilidae and Mycetophilidae). Biodiversity Data Journal.

[B3530465] Jürgenstein Siiri, Kurina Olavi, Põldmaa Kadri (2015). The Mycetophila ruficollis Meigen (Diptera, Mycetophilidae) group in Europe: elucidating species delimitation with COI and ITS2 sequence data. ZooKeys.

[B3530401] Kjærandsen J. Checklist of Nordic fungus gnats (Diptera: Bolitophilidae, Diadocidiidae, Ditomyiidae, Keroplatidae, Mycetophilidae and Sciarosoma). Latest update 13.5.2016.. http://sciaroidea.info/node/48341#.

[B3530389] Kjærandsen J., Hedmark K., Kurina O., Polevoi A. V., Økland B., Götmark F. (2007). Annotated checklist of fungus gnats from Sweden (Diptera: Bolitophilidae, Diadocidiidae, Ditomyiidae, Keroplatidae and Mycetophilidae). Insect Systematics & Evolution, Supplement.

[B3530841] Kurina O. (2004). Redescription of Sciophila nitens Winnertz (Diptera: Mycetophilidae) with a new synonymization. Entomologica Fennica.

[B3530807] Kurina O., Ziegler J. (2008). Sciaroidea excl. Sciaridae. Diptera Stelviana - A dipterological perspective on a changing alpine landscape - Results from a survey of the biodiversity of Diptera (Insecta) in the Stilfserjoch National Park (Italy). Studia Dipterologica Supplement. 16.

[B3530362] Kurina O., Hippa H. (2015). A review of the South Pacific Manota Williston (Diptera, Mycetophilidae), with the description of thirteen new species. Zootaxa.

[B3530519] Kurina O., Jürgenstein S. (2013). Two peculiar new Orfelia Costa species from Georgia (Diptera: Keroplatidae). Entomologica Fennica.

[B3530475] Kurina Olavi, Õunap Erki, Põldmaa Kadri (2015). Two new Neuratelia Rondani (Diptera, Mycetophilidae) species from Western Palaearctic: a case of limited congruence between morphology and DNA sequence data. ZooKeys.

[B3530455] Kurina Olavi, Õunap Erki, Ramel Gordon (2011). Baeopterogyna mihalyii Matile (Diptera, Mycetophilidae): association of sexes using morphological and molecular approaches with the first description of females. ZooKeys.

[B3530420] Nielsen Søren, Kristensen Michael, Pape Thomas (2015). Three new Scandinavian species of Culicoides (Culicoides): C. boyi sp. nov., C. selandicus sp. nov. and C. kalix sp. nov. (Diptera: Ceratopogonidae). Biodiversity Data Journal.

[B3531367] Okada I. (1938). Beitrag zur Kenntnis der Ceroplatinen-Fauna Japans (Diptera, Fungivoridae). Insecta Matsumurana.

[B3529818] Økland B. (1999). New rearing records of forest-dwelling Diptera. International Journal of Dipterological Research.

[B3531407] Ostroverkhova G. P. (1979). Fungus-gnats (Diptera, Mycetophiloidea) of Siberia.

[B3530583] Pape T., Thompson F. C. Systema Dipterorum, Version 1.5. Last updated: 13 June 2013. http://www.diptera.org.

[B3530326] Pape T., Bickel D., Meier R. (2009). Diptera diversity. Status, challenges and tools..

[B3530768] Plassmann E. (1977). Revision der europäischen Arten der Pilzmückengattung Phronia (Diptera: Mycetophilidae). Deutsch. Entomol. Zeitschr. N.F..

[B3531632] Plassmann E. (1980). Neue Pilzmückenfänge aus dem Allgäu (Diptera, Mycetophilidae). Mitteilungen der Münchner Entomologischen Gesellschaft.

[B3531613] Polevoi A. V. (2000). Fungus gnats (Diptera: Bolitophilidae, Ditomyiidae, Keroplatidae, Diadocidiidae, Mycetophilidae) in Karelia.

[B3530626] Polevoi A. V. (2001). New and little known species of the fungus gnat subfamilies Mycomyinae and Sciophilinae (Diptera, Mycetophilidae) from Eastern Fennoscandia. Entomological Review.

[B3531622] Polevoi A. V. (2010). Fungus gnats (Diptera: Bolitophilidae, Keroplatidae, Mycetophilidae) of Pasvik strict nature reserve. Trudy Karel'skogo NC RAN. Seriya biogeografiya.

[B3531334] Ratnasingham S., Hebert P. D.N. (2007). BARCODING: bold: The Barcode of Life Data System (http://www.barcodinglife.org). Molecular Ecology Notes.

[B3529863] Salmela Jukka, Suuronen Anna (2014). A new Neoplatyura Malloch from Finland (Diptera, Keroplatidae). Biodiversity Data Journal.

[B3529853] Salmela Jukka, Suuronen Anna, Kaunisto Kari M (2016). New and poorly known Holarctic species of Boletina Staeger, 1840 (Diptera, Mycetophilidae).. Biodiversity data journal.

[B3531387] Sasakawa M., Kimura T. (1974). Japanese Mycetophilidae (Diptera). 7. Genus *Boletina* Staeger. The Scientific reports of Kyoto Prefectural University (Agric.).

[B3530676] Ševčík J. (2009). Two new species and other new records of fungus gnats (Diptera: Mycetophilidae and Keroplatidae) from Slovakia and the Czech Republic. Čas. Slez. Muz. Opava (A).

[B3530485] Ševčík Jan, Kaspřák David, Rulik Björn (2016). A new species of Docosia Winnertz from Central Europe, with DNA barcoding based on four gene markers (Diptera, Mycetophilidae). ZooKeys.

[B3529805] Ševčík Jan, Kaspřák David, Mantič Michal, Fitzgerald Scott, Ševčíková Tereza, Tóthová Andrea, Jaschhof Mathias (2016). Molecular phylogeny of the megadiverse insect infraorder Bibionomorpha sensu lato (Diptera). PeerJ.

[B3530821] Søli G. E.E. (1997). The adult morphology of Mycetophilidae, with a tentative phylogeny of the family (Diptera, Sciaroidea). Entomologica scandinavica Supplement.

[B3529828] Søli G. E., Vockeroth J. R., Matile L., Papp L., Darvas B. (2000). Families of Sciaroidea. Contributions to a Manual of Palaearctic Diptera (with special reference to flies of economic importance). Appendix.

[B3530410] Stur Elisabeth, Borkent Art (2014). When DNA barcoding and morphology mesh: Ceratopogonidae diversity in Finnmark, Norway. ZooKeys.

[B3529772] Wiegmann Brian M., Trautwein Michelle D., Winkler Isaac S., Barr Norman B., Kim Jung-Wook, Lambkin Christine, Bertone Matthew A., Cassel Brian K., Bayless Keith M., Heimberg Alysha M., Wheeler Benjamin M., Peterson Kevin J., Pape Thomas, Sinclair Bradley J., Skevington Jeffrey H., Blagoderov Vladimir, Caravas Jason, Kutty Sujatha Narayanan, Schmidt-Ott Urs, Kampmeier Gail E., Thompson F. Christian, Grimaldi David A., Beckenbach Andrew T., Courtney Gregory W., Friedrich Markus, Meier Rudolf, Yeates David K. (2011). Episodic radiations in the fly tree of life.. Proceedings of the National Academy of Sciences of the United States of America.

[B3531567] Winnertz J. (1863). Beitrag zu einer Monographie der Pilzmücken. Verh. Zool.-Bot. Ges. Wien.

[B3530574] Zaitzev A. I. (1982). Holarctic fungus gnats of the genus Sciophila Meig..

[B3530548] Zaitzev A. I. (1994). Fungus gnats of the fauna of Russia and adjacent regions. Part 1.

[B3530787] Zaitzev A. I. (2003). Fungus gnats (Diptera, Sciaroidea) of the fauna of Russia and adjacent regions. Part 2. International Journal of Dipterological Research.

[B3531357] Zaitzev A. I., Menzel F. (1996). New data on the fungus gnats from the Russian Far East (Diptera: Sciaroidea). Beiträge zur Entomologie.

